# Supplementary material for: Genetic effects on migration behavior contribute to increasing spatial differentiation at trait-associated loci in Estonia
Source: iScience. 2025 Nov 12;28(12):114013. doi: 10.1016/j.isci.2025.114013 (PMC12702256; doi:10.1016/j.isci.2025.114013)
Supplement: Document S1. Figures S1–S84, Table S3 [file mmc1.pdf]

iScience, Volume *28*

Supplemental information

Genetic effects on migration behavior contribute to increasing spatial differentiation at trait-associated loci in Estonia

Ivan A. Kuznetsov, Estonian Biobank Research Team, Mait Metspalu, Uku Vainik, Luca Pagani, Francesco Montinaro, and Vasili Pankratov

Content

Supplemental Figures **2**
Estonian Biobank cohort overview (Supplemental Note 1) 2
Intensity of the internal migration in Estonia (Supplemental Note 2) 5
Robustness of Var_{county} estimates (Supplemental Note 4) 7
Replication of the main results in unrelated Estonian individuals (Supplementary Note 5) ... 17
Replication of the main results with PGS_{EA4} (Supplemental Note 6) 21
Analysis of psychiatric trait PGSs (Supplemental Note 7) 26
Selective migration and correlations between mate-pair PGSs (Supplemental Note 8) 28
How large are the regional differences in PGS_{EA}? (Supplemental Note 9) 29
Genetic predictors of ORE-to-cities migration 30
Geographical distribution of (s)PGS_{EA} 38
Geographical distribution of educational attainment phenotype 42
(s)PGS_{EA} values in groups with different migration profiles 47
Migration direction and (s)PGS_{EA} 52
Educational attainment phenotype in groups with different migration profiles 58
(s)PGS_{EA} with EA regressed out in groups with different migration profiles 68
Supplemental Tables **81**

Supplemental Figures

Estonian Biobank cohort overview (Supplemental Note 1)

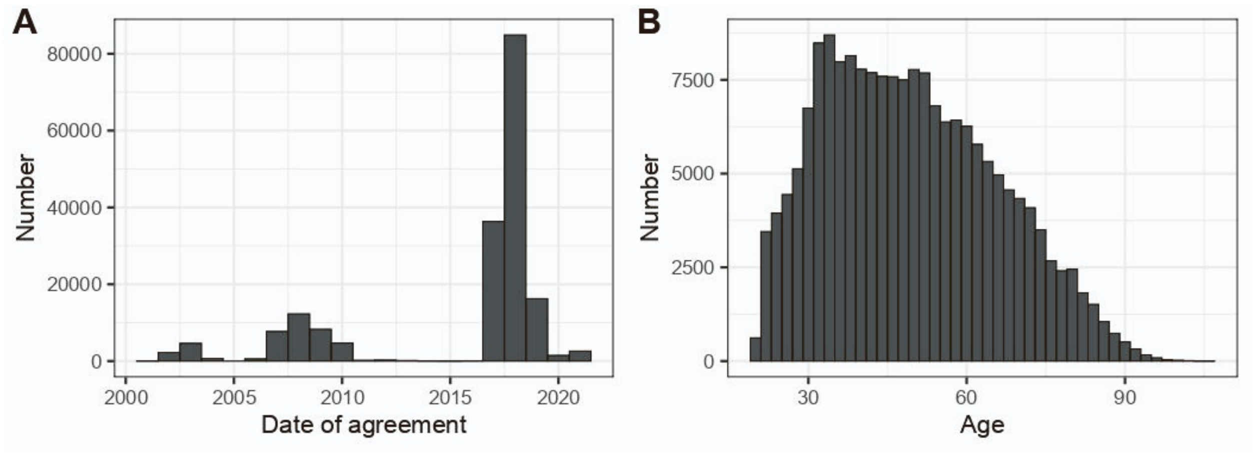

Figure S1. The distribution of EstBB participants after filtering by (A) date of agreement (year of recruitment) and (B) age.

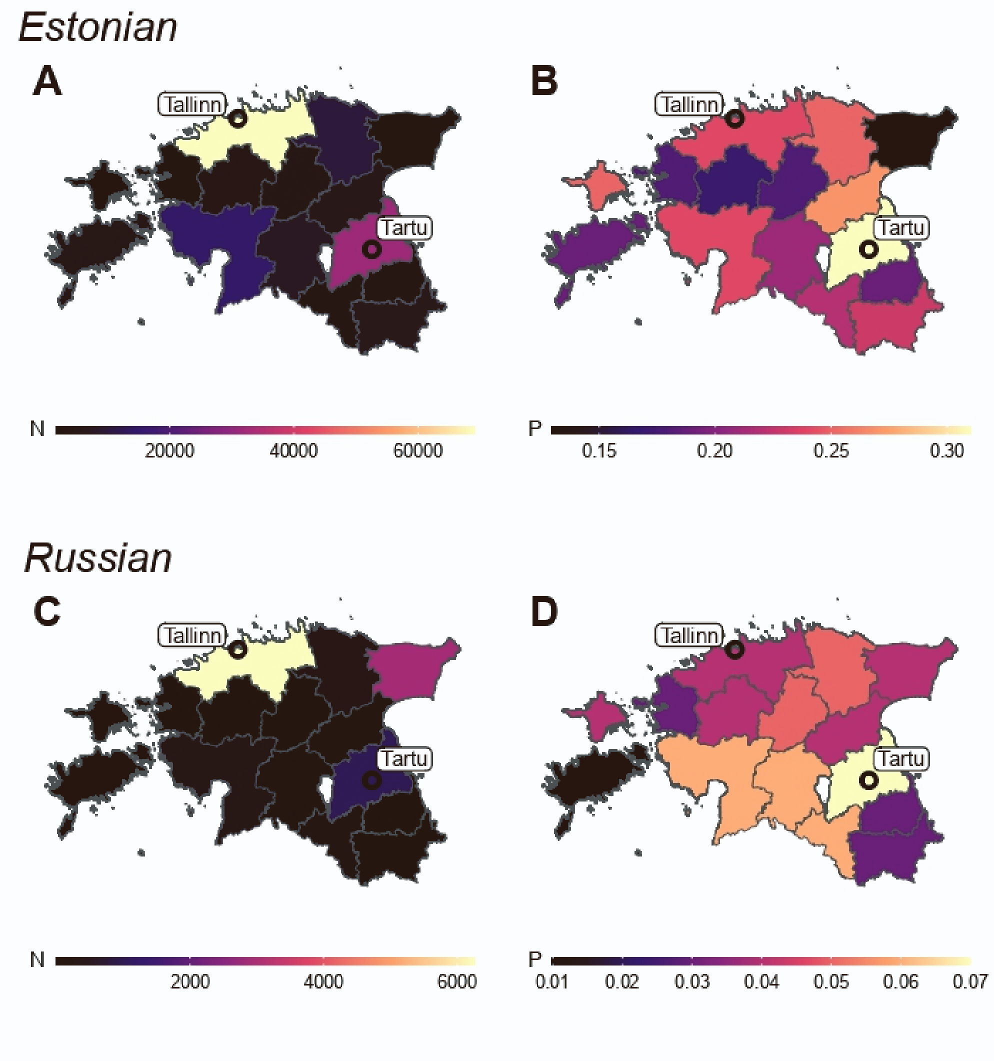

Figure S2. Geographic distribution of EstBB participants by county of residence. (A) The number of EstBB participants of self-reported Estonian ethnicity ; (B) the fraction of EstBB participants among residents of self-reported Estonian ethnicity; (C) the number of EstBB participants of self-reported Russian ethnicity; (D) the fraction of EstBB participants among residents of self-reported Russian ethnicity. Data on the number of current residents per county was taken from the 2021 Population census[4].

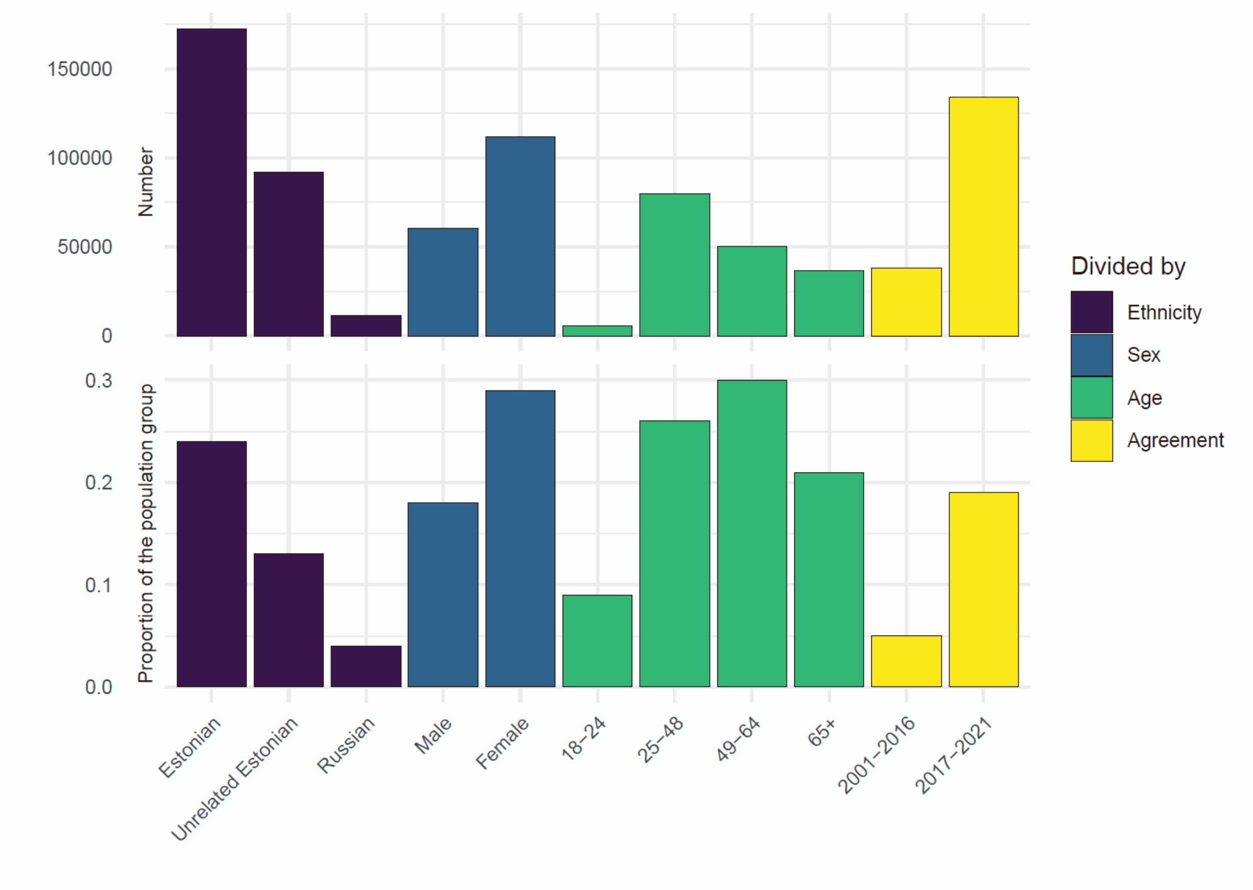

Figure S3. Absolute number of participants in each group (top) and the same, normalized by the size of the corresponding group in the general population (bottom). In the bottom panel, the proportions of "Estonians", "Unrelated Estonians" and the groups by the year of agreement (yellow) are shown relative to number of adult self-identified Estonians in the population. Census population data was taken from the 2021 Population census[4].

Intensity of the internal migration in Estonia (Supplemental Note 2)

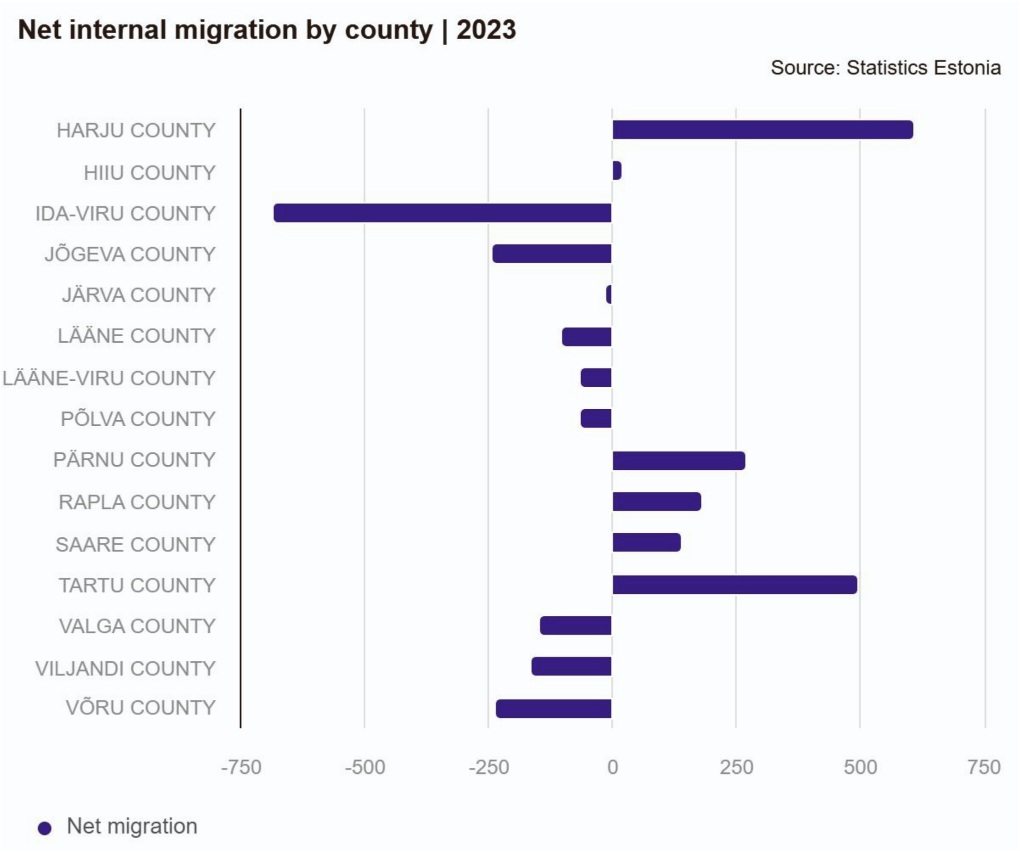

Figure S4. Net internal migration in Estonia by county in 2023. Source: Statistics Estonia[6].

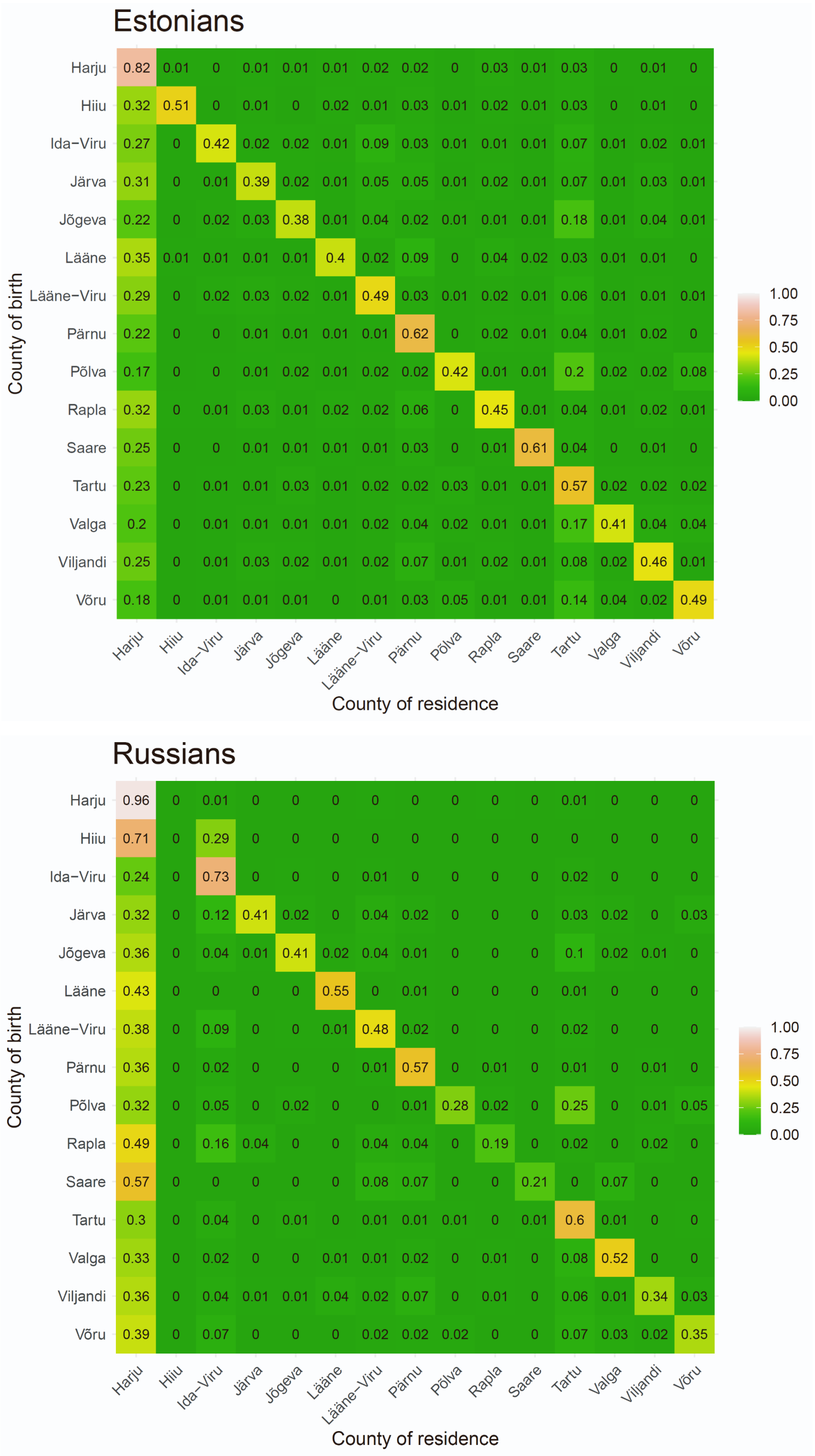

Figure S5. Internal migration in Estonia. The fractions of individuals of Estonian (top) and Russian (bottom) self-reported ethnicity born in each of the counties who stayed in the county of birth or moved to another county.

Robustness of Var_{county} estimates (Supplemental Note 4)

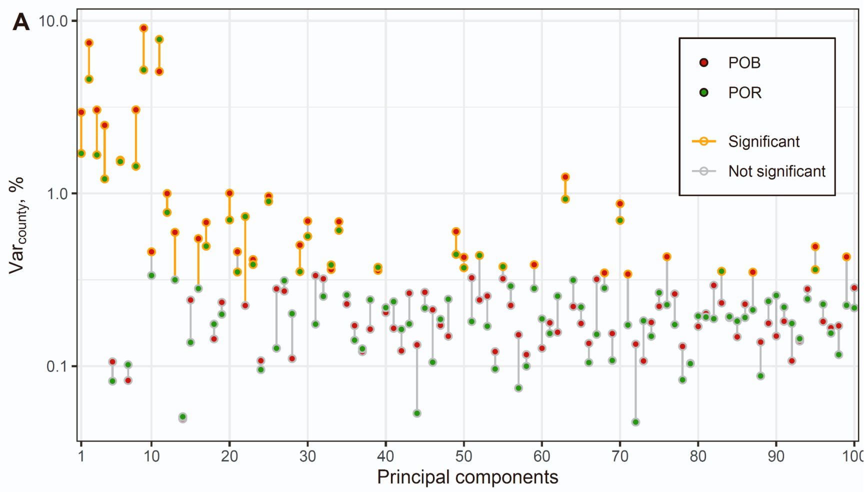

Figure S6. Inter-individual variance of PCs among Russian participants, explained by county of birth (POB) and county of residence (POR). The PCs are derived from the Russian subcohort. Red and green dots refer to the POB and POR, correspondingly. Estimates significantly different from zero are outlined in yellow. The line connecting the two points is yellow when the variance explained by POB and POR together is significantly larger than the variance explained by only the weaker predictor. The significance level is 0.05, adjusted for 100 tests with Bonferroni correction.

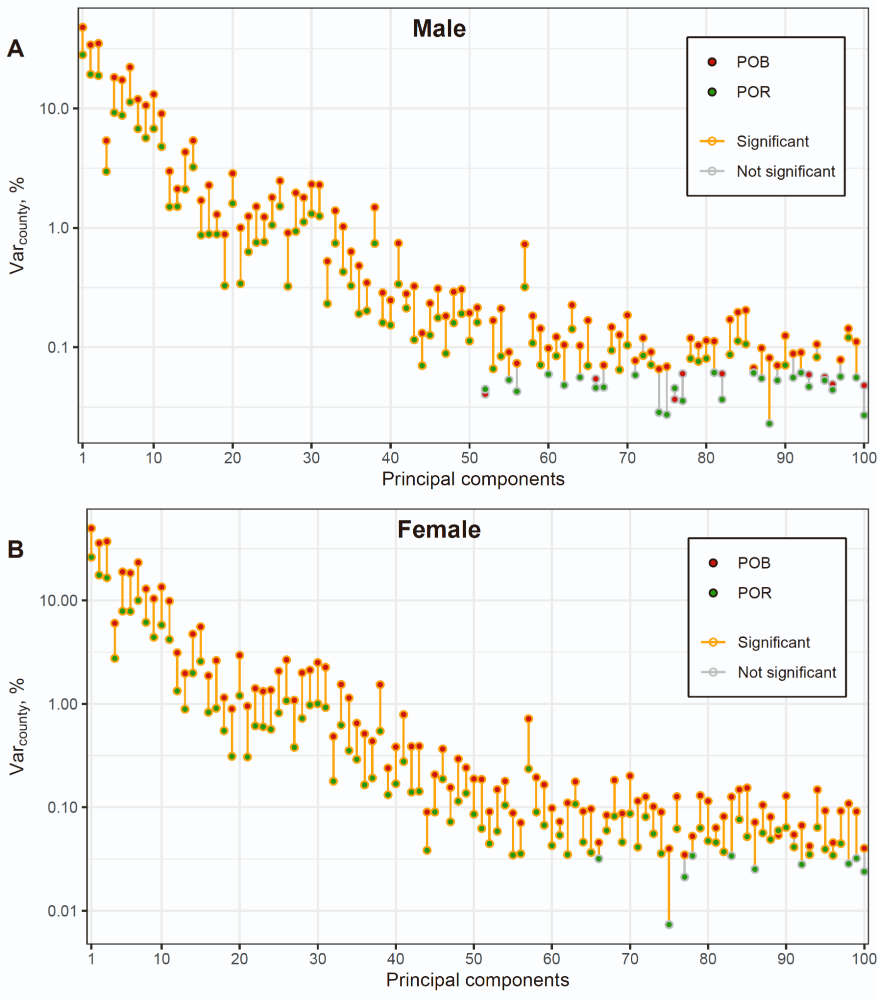

Figure S7. Inter-individual variance of PCs among (A) male and (B) female Estonian participants explained by county of birth (POB) and county of residence (POR). Red and green dots refer to the POB and POR, correspondingly. Estimates significantly different from zero are outlined in yellow. The line connecting the two points is yellow when the variance explained by POB and POR together is significantly larger than the variance explained by only the weaker predictor. The significance level is 0.05, adjusted for 100 tests with Bonferroni correction.

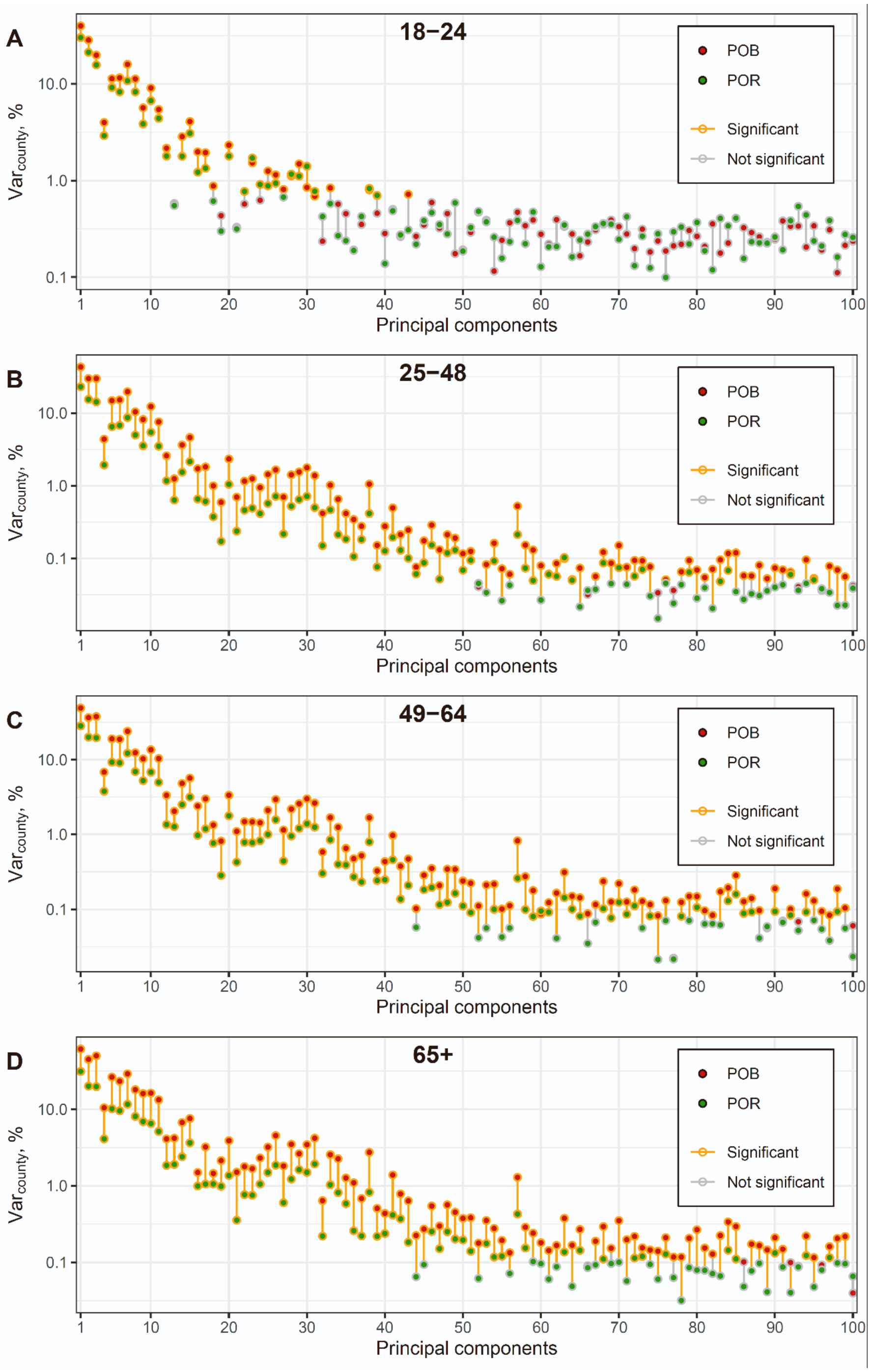

Figure S8. Inter-individual variance of PCs among Estonian participants stratified by age explained by county of birth (POB) and county of residence (POR). Age groups were defined as (A) 18-24, (B) 25-48, (C) 49-64, (D) 65+. Red and green dots refer to the POB and POR, correspondingly. Estimates significantly different from zero are outlined in yellow. The line connecting the two points is yellow when the variance explained by POB and POR together is significantly larger than the variance explained by only the weaker predictor. The significance level is 0.05, adjusted for 100 tests with Bonferroni correction.

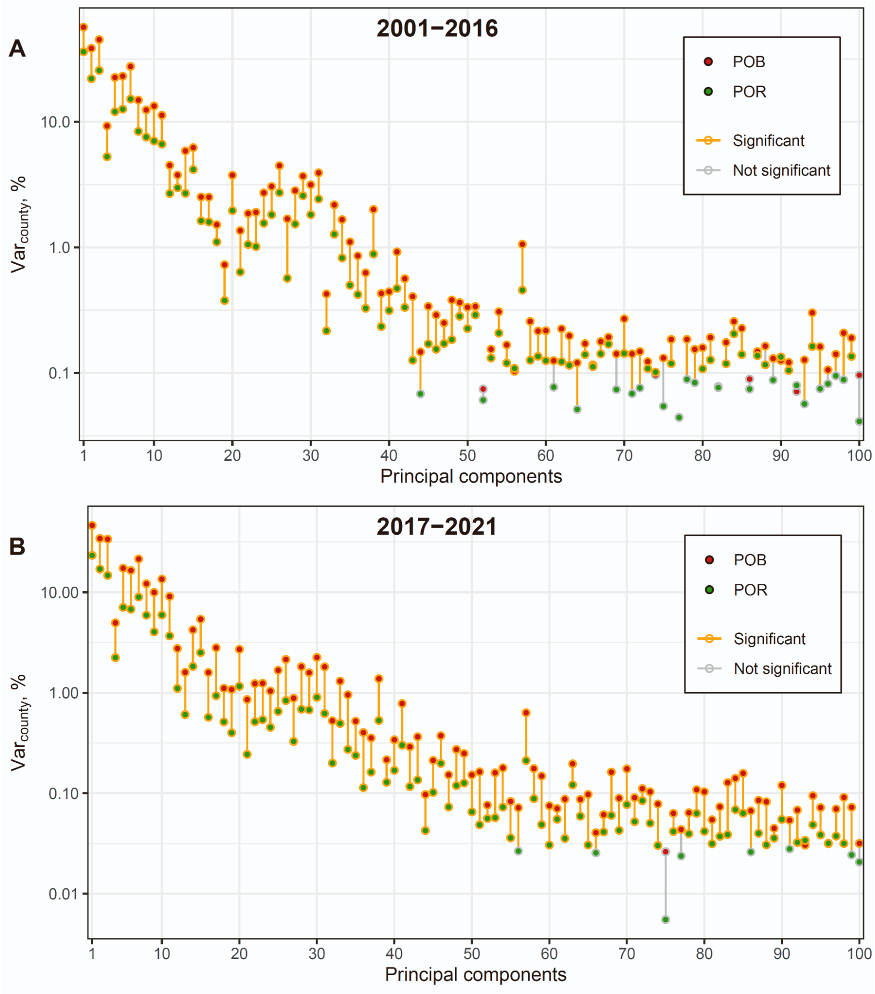

Figure S9. Inter-individual variance of PCs among Estonian participants stratified by year of joining the biobank explained by county of birth (POB) and county of residence (POR). The periods of joining are (A) 2001-2016 and (B) 2017-2021. Red and green dots refer to the POB and POR, correspondingly. Estimates significantly different from zero are outlined in yellow. The line connecting the two points is yellow when the variance explained by POB and POR together is significantly larger than the variance explained by only the weaker predictor. The significance level is 0.05, adjusted for 100 tests with Bonferroni correction.

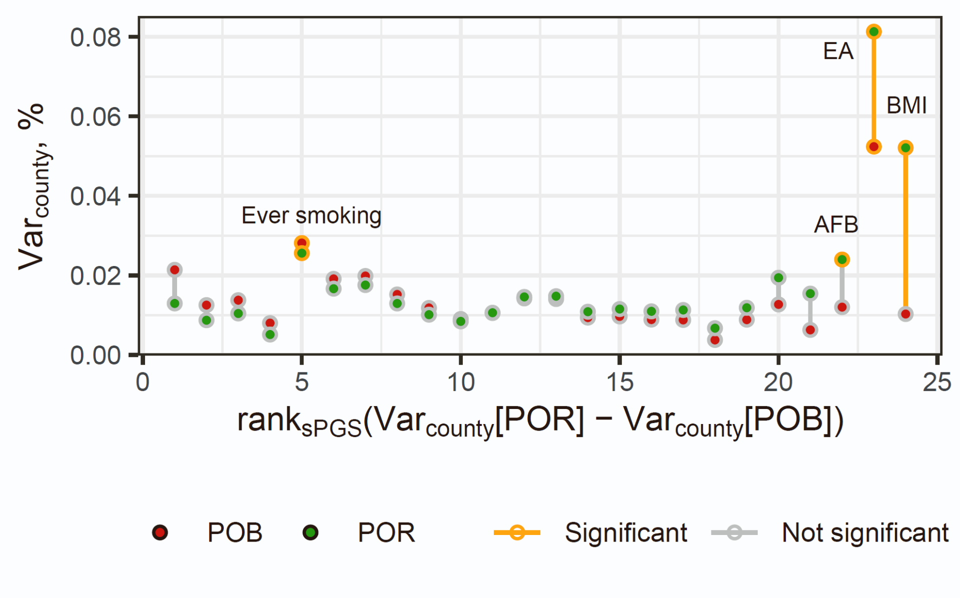

Figure S10. Estimates of the inter-individual variance of sPGSs among Estonian participants explained by POB and POR. sPGSs are adjusted for demographic and genetic ancestry covariates. sPGSs are ordered according to the rank of difference between Var_{county} for POR and POB. Estimates significantly different from zero are outlined in yellow and labeled (*BMI* - Body Mass Index; *EA* - educational attainment; *AFB* - age at first birth). The line connecting the two points is yellow when the variance explained by POB and POR together is significantly larger than the variance explained by only the weaker predictor. The significance level is 0.05, adjusted for the number of sPGS tested with Bonferroni correction.

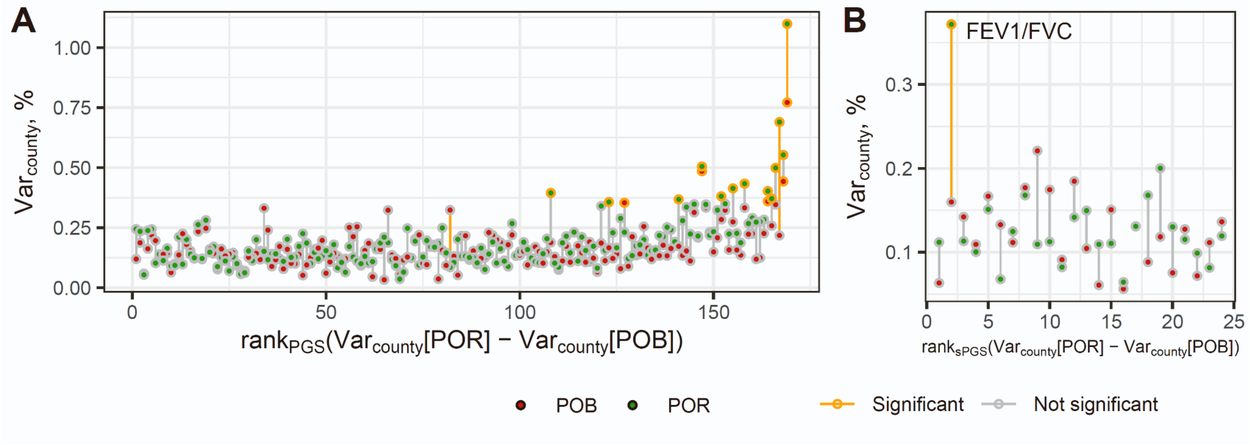

Figure S11. Estimates of the inter-individual variance of (A) PGSs and (B) sPGSs among Russian participants explained by POB and POR. (s)PGSs are adjusted for demographic and genetic ancestry covariates. PGSs and sPGSs are ordered according to the rank of difference between Var_{county} for POR and POB in the full Estonian subsample (as in Figure 1B and Figure S10 accordingly). Estimates significantly different from zero are outlined in yellow. The line connecting the two points is yellow when the variance

explained by POB and POR together is significantly larger than the variance explained by only the weaker predictor. The significance level is 0.05, adjusted for the number of (s)PGS tested with Bonferroni correction.

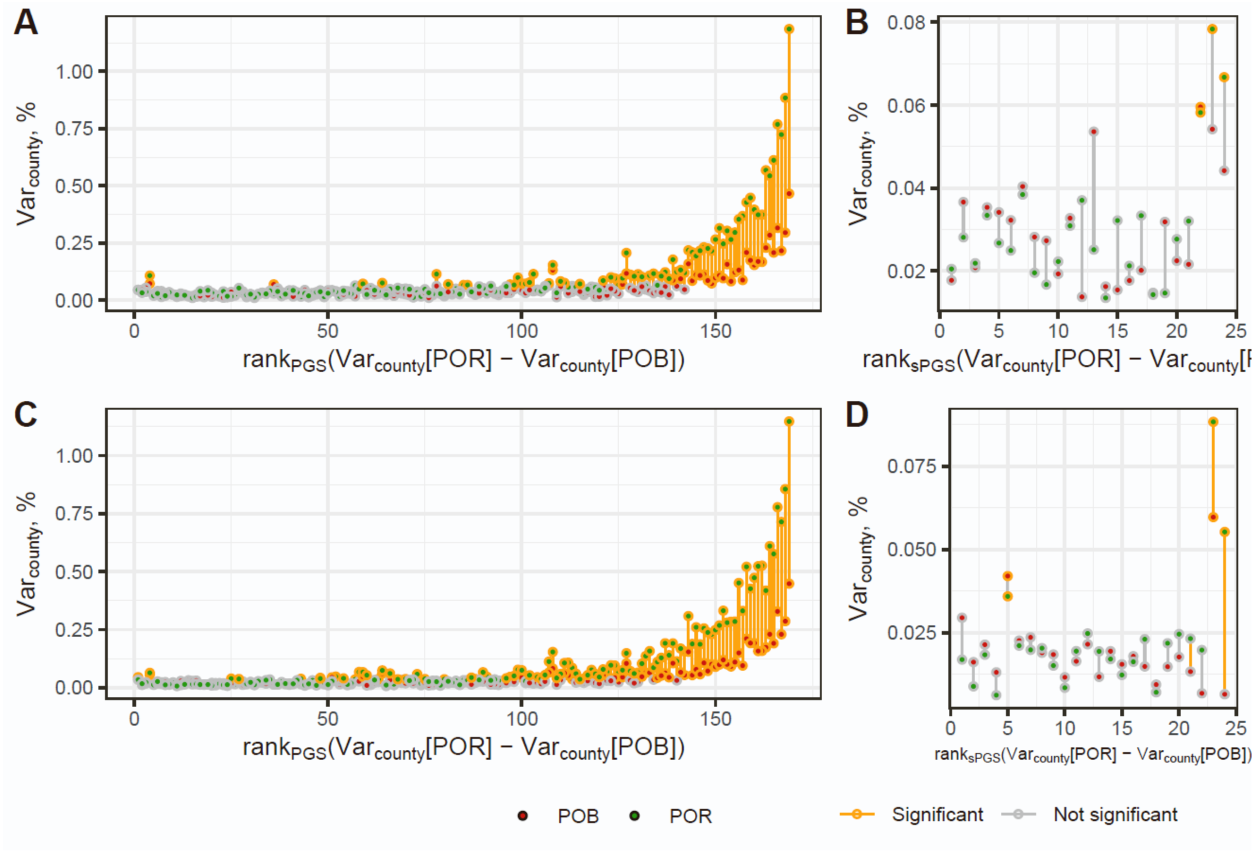

Figure S12. Estimates of the inter-individual variance of (A, C) PGSs and (B, D) sPGSs among (A-B) male and (C-D) female Estonian participants explained by POB and POR. (s)PGSs are adjusted for demographic and genetic ancestry covariates. PGSs and sPGSs are ordered according to the rank of difference between Var_{county} for POR and POB in the full Estonian subsample. Estimates significantly different from zero are outlined in yellow. The line connecting the two points is yellow when the variance explained by POB and POR together is significantly larger than the variance explained by only the weaker predictor. The significance level is 0.05, adjusted for the number of (s)PGS tested with Bonferroni correction.

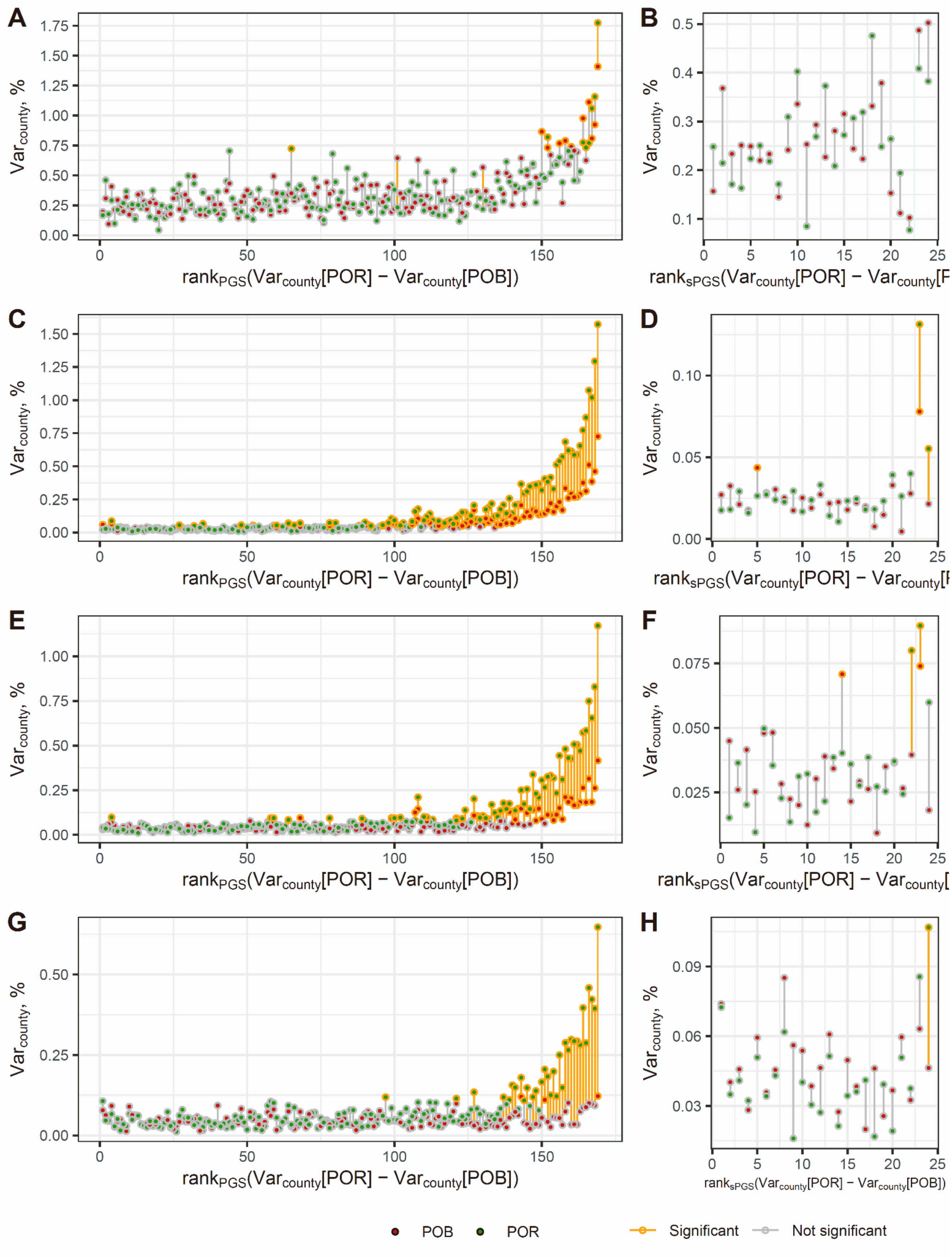
A
B
C
D
E
F
G
H
Var$_{county}$, %
rank$_{PGS}$(Var$_{county}$[POR] − Var$_{county}$[POB])
rank$_{sPGS}$(Var$_{county}$[POR] − Var$_{county}$[POB])
POB
POR
Significant
Not significant

Figure S13. Estimates of the inter-individual variance of (A, C, E, G) PGSs and (B, D, F, H) sPGSs among Estonian participants stratified by age explained by POB and POR. Age groups were defined as (A-B) 18-24, (C-D) 25-48, (E-F) 49-64, (G-H) 65+. (s)PGSs are adjusted for demographic and genetic ancestry covariates. PGSs and sPGSs are ordered according to the rank of difference between Var_{county} for POR and POB in the full Estonian subsample. Estimates significantly different from zero are outlined in yellow. The line connecting the two points is yellow when the variance explained by POB and POR together is significantly larger than the variance explained by only the weaker predictor. The significance level is 0.05, adjusted for the number of (s)PGS tested with Bonferroni correction.

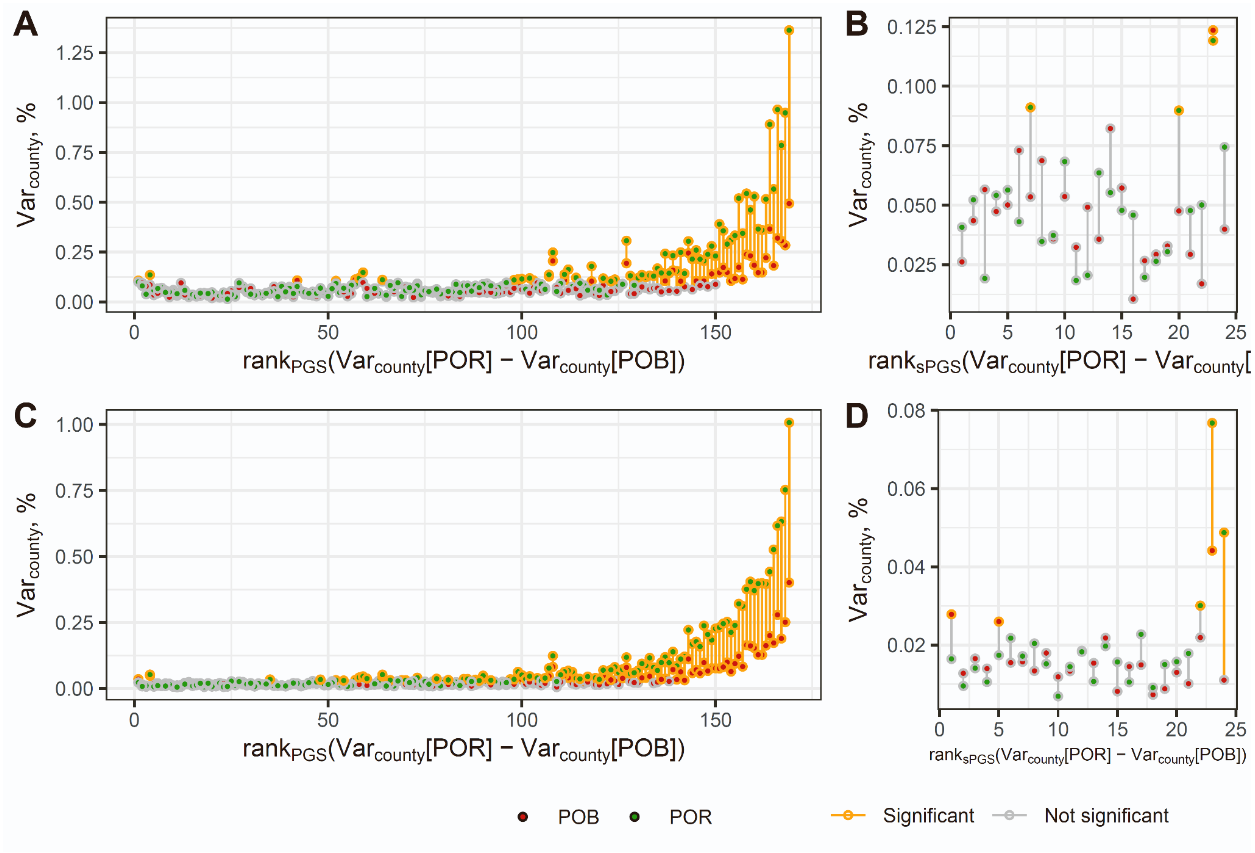

Figure S14. Estimates of the inter-individual variance of (A, C) PGSs and (B, D) sPGSs among Estonian participants stratified by year of joining the biobank explained by POB and POR. The periods of joining are (A-B) 2001-2016 and (C-D) 2017-2021. (s)PGSs are adjusted for demographic and genetic ancestry covariates. PGSs and sPGSs are ordered according to the rank of difference between Var_{county} for POR and POB in the full Estonian subsample. Estimates significantly different from zero are outlined in yellow. The line connecting the two points is yellow when the variance explained by POB and POR together is significantly larger than the variance explained by only the weaker predictor. The significance level is 0.05, adjusted for the number of (s)PGS tested with Bonferroni correction.

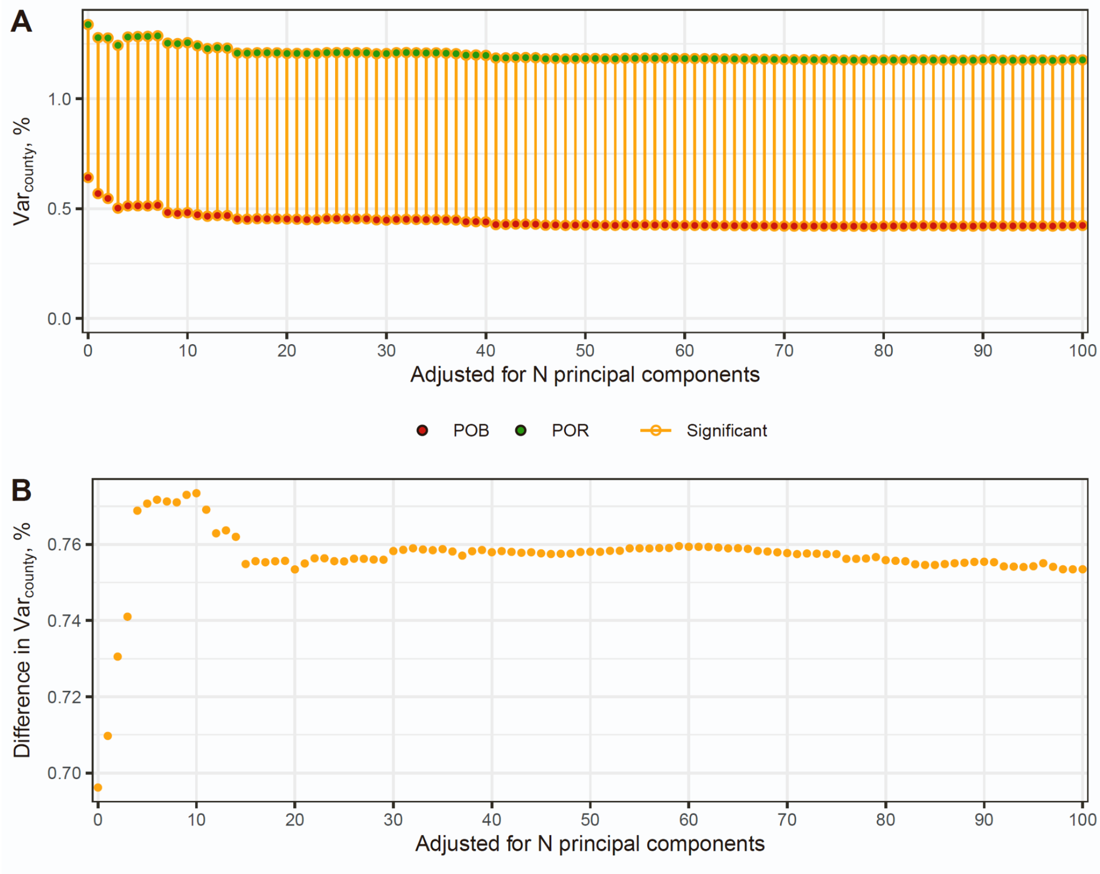

Figure S15. (A) Fraction of the inter-individual variance of PGS_{EA} cumulative adjusted for the top 100 PCs, explained by county of birth (POB) and county of residence (POR) and (B) the difference between Var_{county} for POR and POB. PGS_{EA} is also adjusted for demographic covariates in all the models. Red and green dots refer to the POB and POR, correspondingly. Estimates significantly different from zero are outlined in yellow. The line connecting the two points (in A) and the points (in B) are yellow when the variance explained by POB and POR together is significantly larger than the variance explained by only the weaker predictor (if significant) or when the stronger predictor is significant. The significance level is 0.05, after Bonferroni correction for 169 tests.

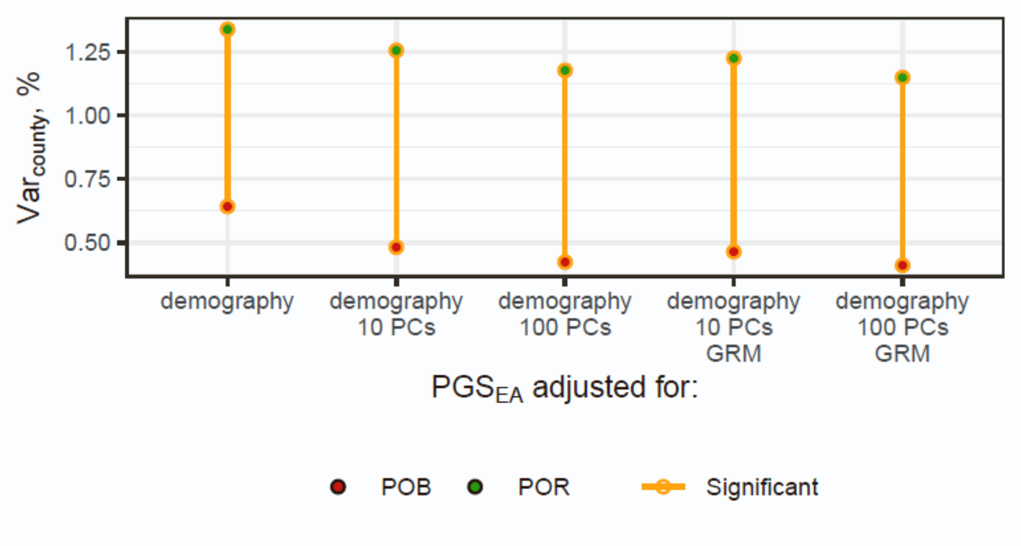

Figure S16. Fraction of the inter-individual variance of PGS_{EA} adjusted for PCs and GRM, explained by county of birth (POB) and county of residence (POR). PGS_{EA} is also adjusted for demographic covariates in all the models. Red and green dots refer to the POB and POR, correspondingly. Estimates significantly different from zero are outlined in yellow. The line connecting the two points are yellow when the variance explained by POB and POR together is significantly larger than the variance explained by only the weaker predictor (if significant) or when the stronger predictor is significant. The significance level is 0.05, after Bonferroni correction for 169 tests.

Replication of the main results in unrelated Estonian individuals (Supplementary Note 5)

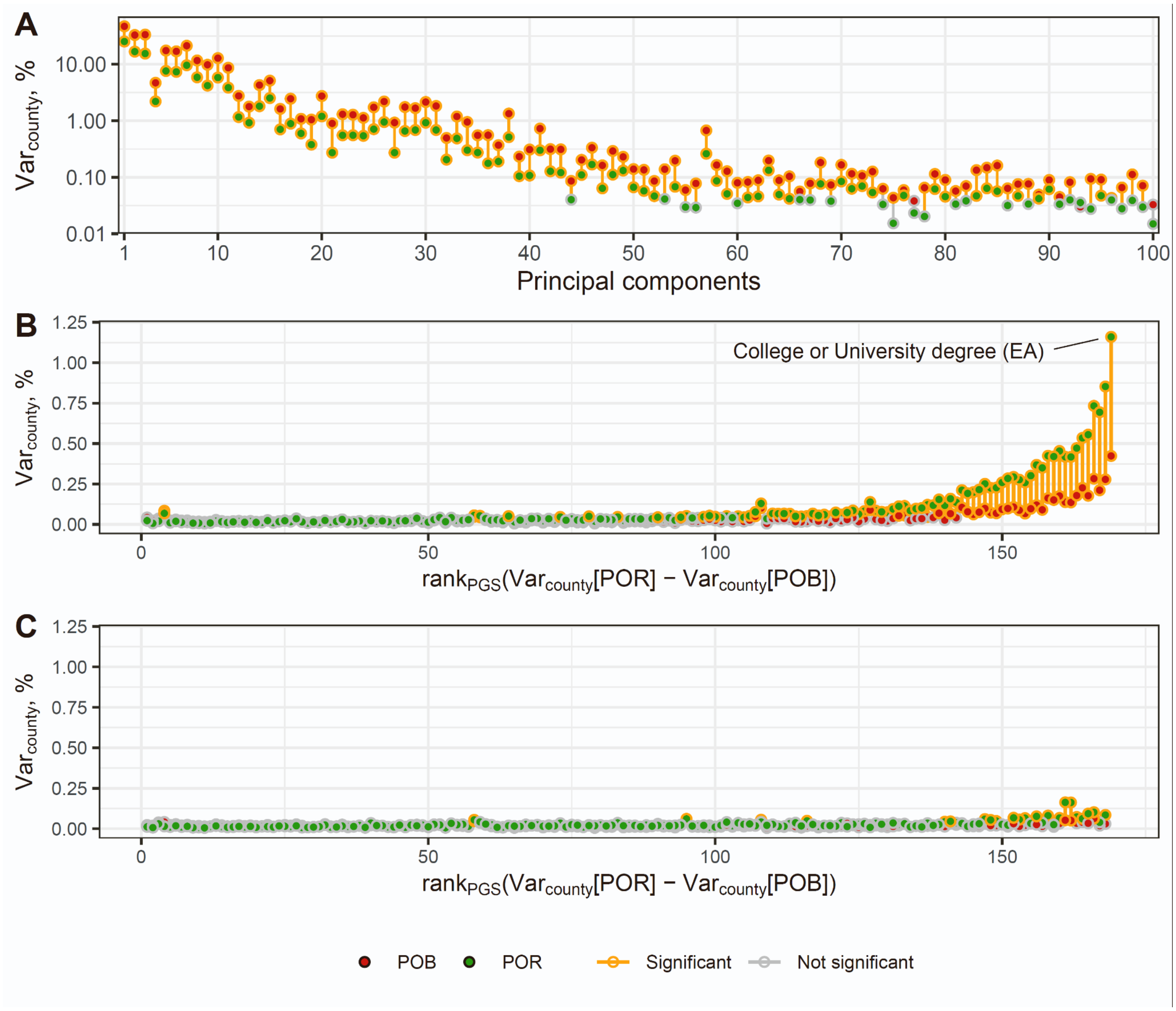

Figure S17. Fraction of the inter-individual variance of (A) PCs, (B) PGSs and (C) PGSs additionally adjusted for PGS$_{EA}$, explained by county of birth (POB) and county of residence (POR) among unrelated Estonian participants. PGSs are preliminary adjusted for the top 100 PCs and demographic covariates. The y-axis in panel A has a logarithmic scale. The PGSs on the x-axis in panels B and C are ordered according to the rank of difference between Var_{county} for POR and POB in the full Estonian subsample (as in Figure 1B). Red and green dots refer to the POB and POR, correspondingly. Estimates significantly different from zero are outlined in yellow. The line connecting the two points is yellow when the variance explained by POB and POR together is significantly larger than the variance

explained by only the weaker predictor (if significant) or when the stronger predictor is significant. The significance level is 0.05, after Bonferroni correction.

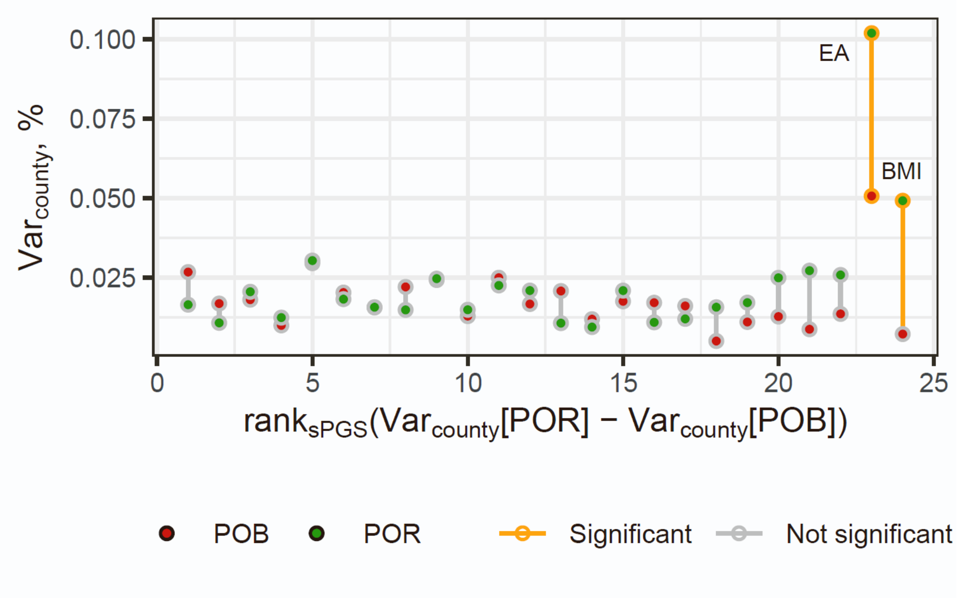

Figure S18. Estimates of the inter-individual variance of sPGSs among unrelated Estonian participants explained by POB and POR. sPGSs are adjusted for demographic and genetic ancestry covariates. PGSs and sPGSs are ordered according to the rank of difference between Var_{county} for POR and POB in the full Estonian subsample. Estimates significantly different from zero are outlined in yellow. The line connecting the two points is yellow when the variance explained by POB and POR together is significantly larger than the variance explained by only the weaker predictor. The significance level is 0.05, adjusted for the number of sPGS tested with Bonferroni correction.

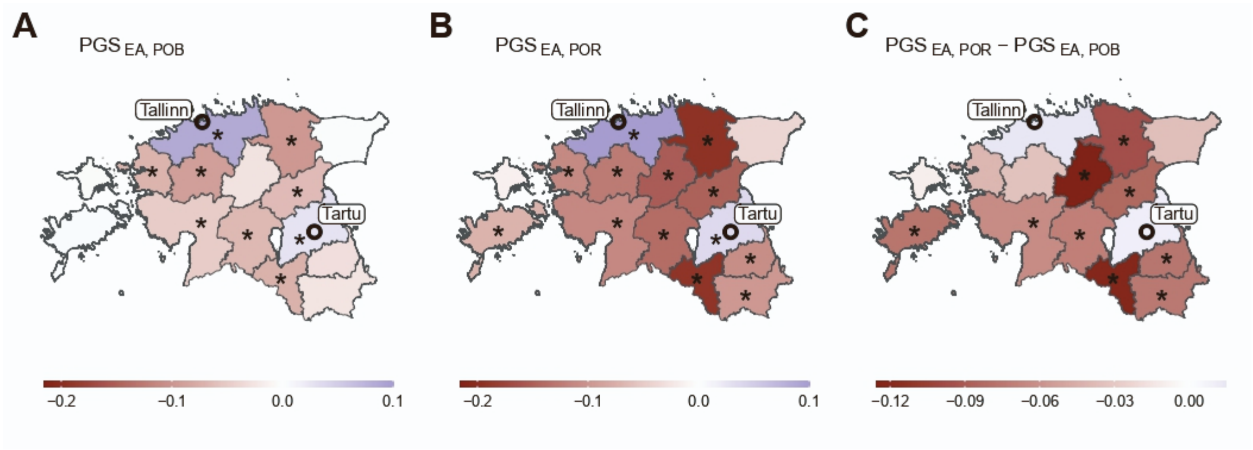

Figure S19. PGS_{EA} landscape in Estonia among unrelated Estonian participants. Mean PGS_{EA} of individuals (A) born or (B) residing in each county. (C) Differences between values in "B" and "A" panels. PGS_{EA} is adjusted for demographic and genetic ancestry covariates. Counties with sample mean values significantly different from zero after FDR correction at the 0.05 level are marked with an asterisk (*).

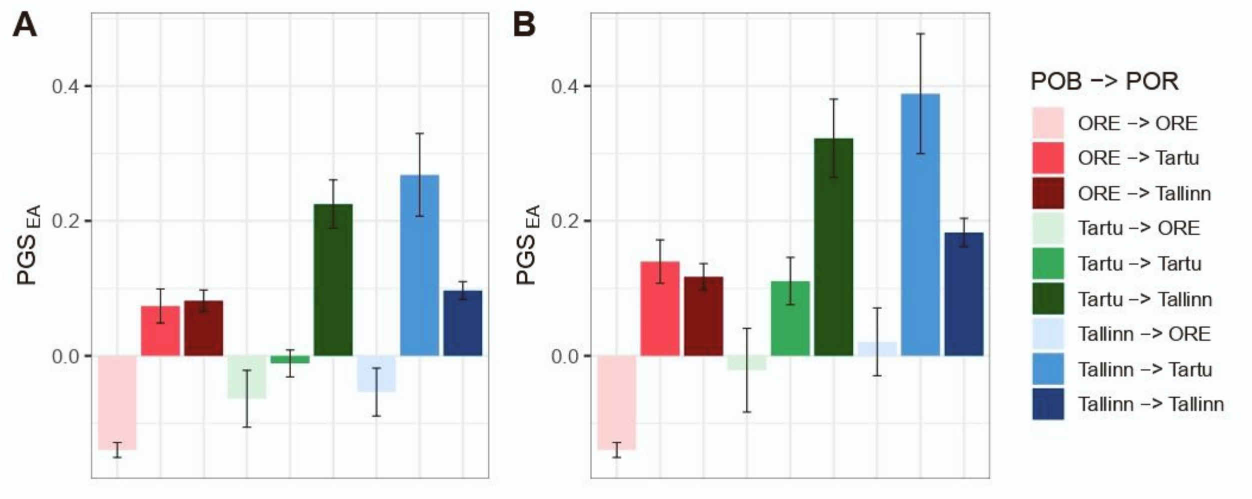

Figure S20. PGS_{EA} in migration groups among unrelated Estonian participants by region of birth (POB) and residence (POR). (A) County-based analysis where POB and POR refer to Tartu County ("Tartu"), Harju County ("Tallinn") and other counties ("ORE"). (B) City-based analysis, where POB and POR refer to Tartu City ("Tartu"), Tallinn ("Tallinn") and other counties ("ORE"). PGS_{EA} is adjusted for demographic and genetic ancestry covariates. Error bars correspond to 95% confidence intervals.

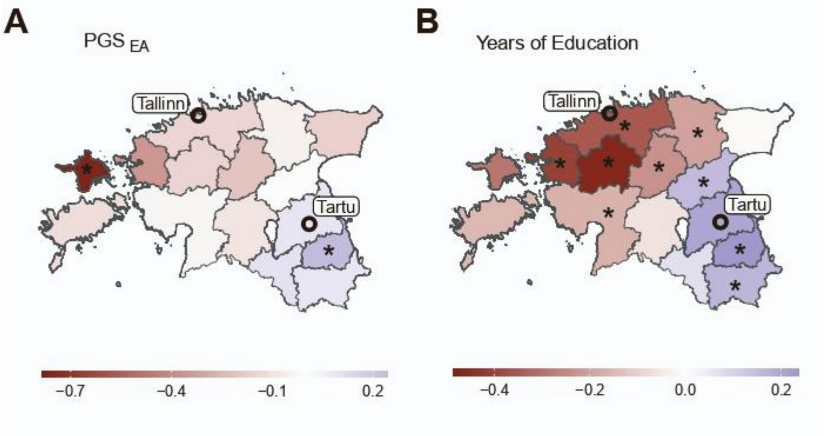

Figure S21. The contrast in mean PGS_{EA} and EA (years of education) between residents of Tallinn and Tartu City among unrelated Estonian participants by county of birth. (A) The value for each county corresponds to the mean PGS_{EA} of individuals born in that county and living in Tartu City

subtracted from the mean PGS_{EA} of individuals born in the same county and living in Tallinn. Individuals born in Tallinn or Tartu City are excluded from the analysis. (B) The same but for the "years of education" phenotype. Counties with significant differences between the migrant groups after FDR correction at level 0.05 are marked with an asterisk (*).

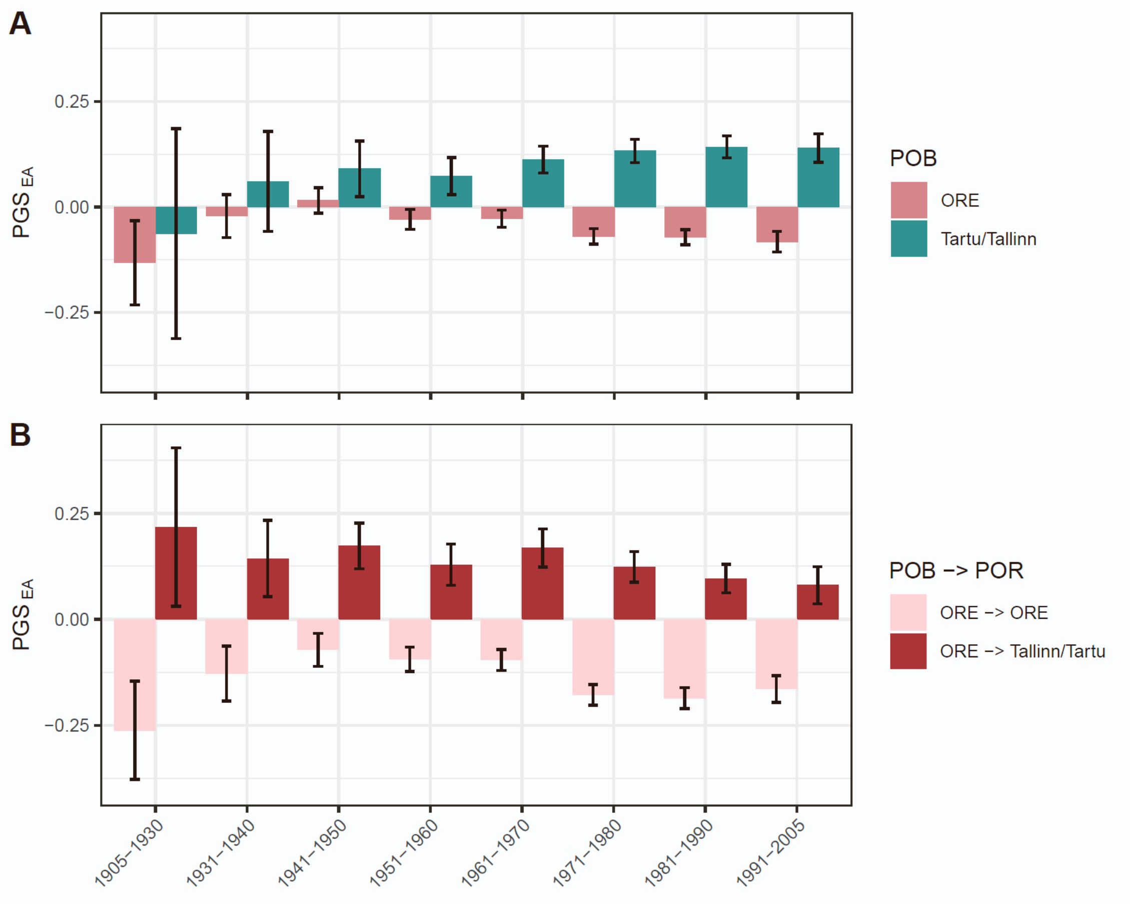

Figure S22. Difference in average PGS_{EA} between cities (Tallinn and Tartu combined) and ORE across birth year bins in the sample of unrelated Estonians. (A) Mean PGS_{EA} of individuals born in either ORE or Tallinn/Tartu; (B) mean PGS_{EA} of individuals born in ORE and residing in either ORE or Tallinn/Tartu. PGS_{EA} is adjusted for the top 100 PCs and demographic covariates. Error bars correspond to 95% confidence intervals.

Replication of the main results with PGS_{EA4} (Supplemental Note 6)

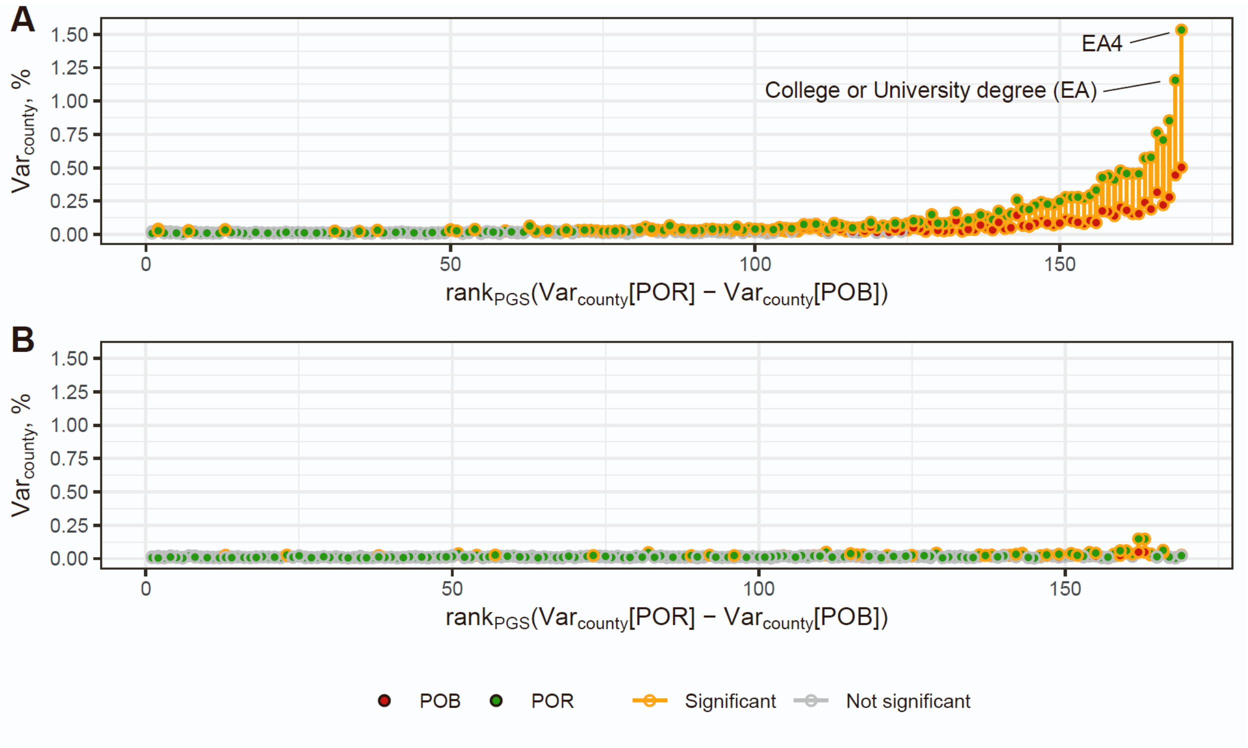

Figure S23. Fraction of the inter-individual variance of (A) PGSs and (B) PGSs additionally adjusted for PGS_{EA4}, explained by county of birth (POB) and county of residence (POR). PGSs are preliminary adjusted for the top 100 PCs and demographic covariates. In panel A the PGSs on the x-axis are ordered according to the difference between POR and POB which is the same order as in Figure 1B with the addition of PGS_{EA4}. In panel B the order is the same as in panel A but without PGS_{EA4}. Var_{county} for PGSs adjusted for the top 100 PCs and demographic covariates. Red and green dots refer to the POB and POR, correspondingly. Estimates significantly different from zero are outlined in yellow. The line connecting the two points is yellow when the variance explained jointly by POB and POR is significantly larger than the variance explained by only the weaker predictor (if significant) or when the stronger predictor is significant. The significance level is 0.05, after Bonferroni correction.

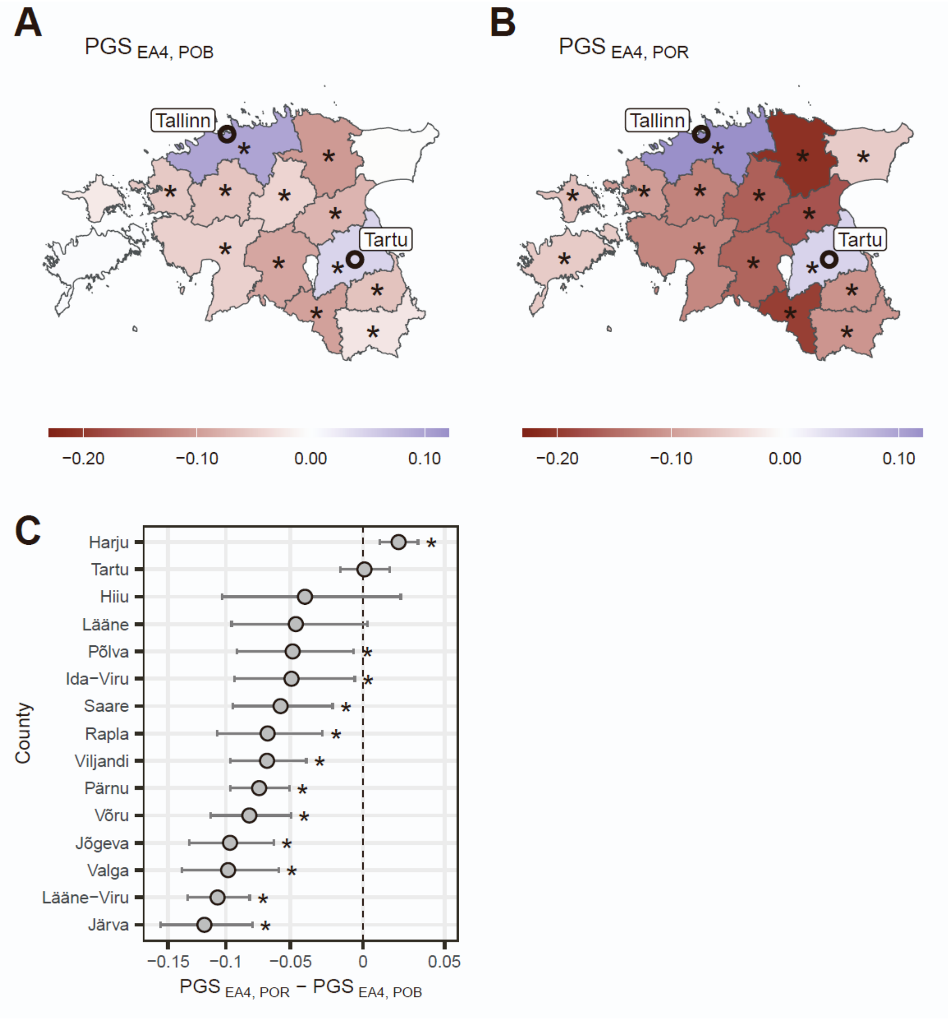

Figure S24. PGS_{EA4} landscape in Estonia. Mean PGS_{EA4} of individuals (A) born or (B) residing in each county. (C) Difference between values in panels B and A. PGS_{EA4} is adjusted for the top 100 PCs and demographic covariates. Counties with the corresponding value being significantly different from zero after FDR correction at the 0.05 significance level are marked with an asterisk (*). Error bars correspond to 95% confidence intervals. PGS_{EA4} is measured in standard deviations.

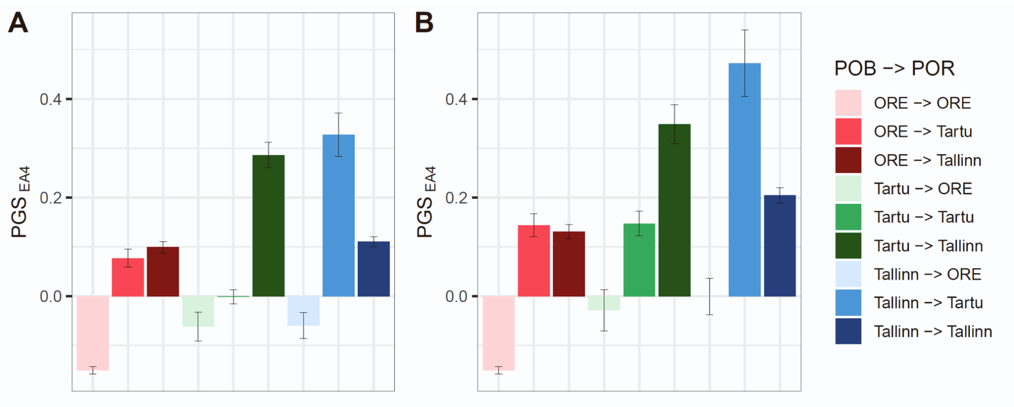

Figure S25. PGS_{EA4} in migration groups defined by combination of place of birth (POB) and residence (POR). (A) County-based analysis where "Tartu" and "Tallinn" refer to Tartu County and Harju County respectively while "ORE" refers to other counties. (B) City-based analysis, where "Tartu" and "Tallinn" refer to the respective cities while "ORE" refers to other counties as in A. PGS_{EA} is adjusted for the top 100 PCs and demographic covariates. In all panels error bars correspond to 95% confidence intervals.

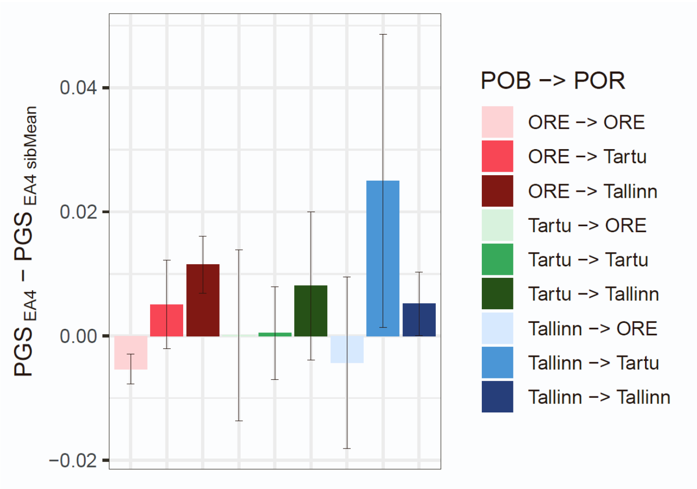

Figure S26. PGS_{EA4} adjusted for sibship-average in migration groups defined by combination of place of birth (POB) and residence (POR). "Tartu" and "Tallinn" refer to Tartu County and Harju County respectively while "ORE" refers to other counties. Error bars correspond to 95% confidence intervals.

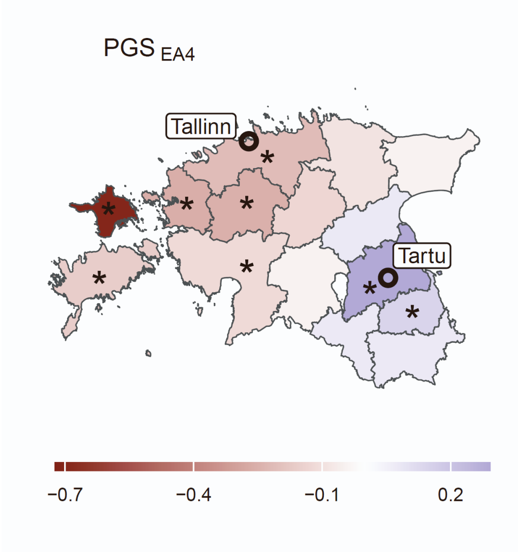

Figure S27. The difference in mean PGS_{EA4} between residents of Tallinn and Tartu City by county of birth. The value for each county corresponds to the mean PGS_{EA4} of individuals born in that county and residing in Tartu City subtracted from the mean PGS_{EA} of individuals born in the same county and residing in Tallinn. Individuals born in Tallinn or Tartu City are excluded from the analysis. PGS_{EA} is adjusted for the top 100 PCs and demographic covariates. Counties with the difference being significant after FDR correction at level 0.05 are marked with an asterisk (*).

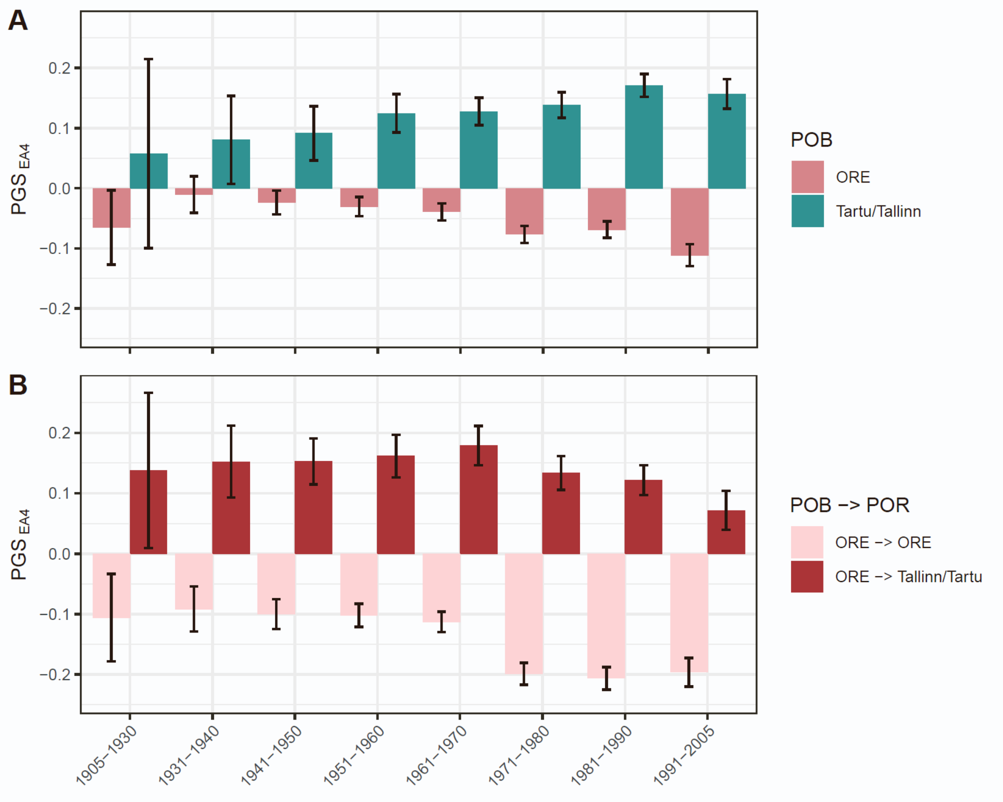

Figure S28. Difference in average PGS_{EA4} between cities (Tallinn and Tartu combined) and ORE across birth year bins. (A) Mean PGS_{EA} of individuals born in either ORE or Tallinn/Tartu; (B) mean PGS_{EA4} of individuals born in ORE and residing in either ORE or Tallinn/Tartu. PGS_{EA4} is adjusted for the top 100 PCs and demographic covariates. Error bars correspond to 95% confidence intervals.

Analysis of psychiatric trait PGSs (Supplemental Note 7)

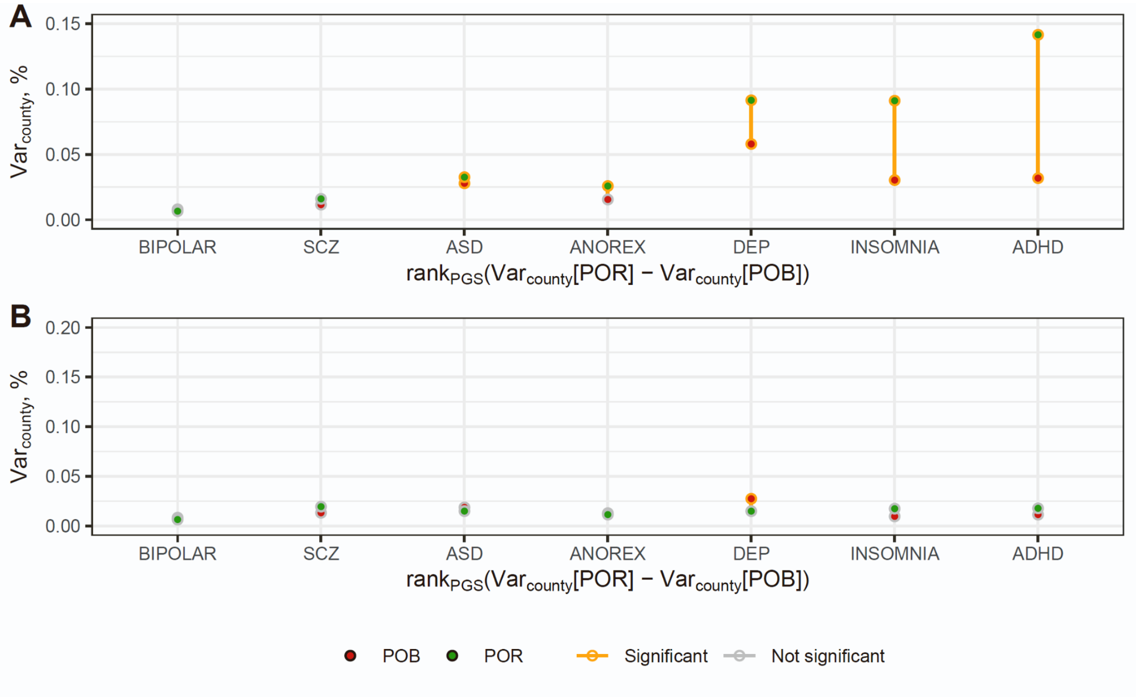

Figure S29. Fraction of the inter-individual variance of (A) PGSs for psychiatric disorders and (B) those PGSs additionally adjusted for PGS_{EA4}, explained by county of birth (POB) and county of residence (POR) among self-reported Estonian participants. "ANOREX" - anorexia nervosa, "ADHD" - attention deficit hyperactivity disorder, "ASD" - autism spectrum disorder, "BIPOLAR" - bipolar disorder, "DEP" - depressive symptoms, "SCZ" - schizophrenia. PGSs are preliminary adjusted for the top 100 PCs and demographic covariates. In panel A the PGSs on the x-axis are ordered according to the difference between POR and POB. In panel B the order is the same as in panel A but without PGS_{EA4}.Var_{county} for PGSs adjusted for the top 100 PCs and demographic covariates. Red and green dots refer to the POB and POR, correspondingly. Estimates significantly different from zero are outlined in yellow. The line connecting the two points is yellow when the variance explained jointly by POB and POR is significantly larger than the variance explained by only the weaker predictor (if significant) or when the stronger predictor is significant. The significance level is 0.05, after Bonferroni correction.

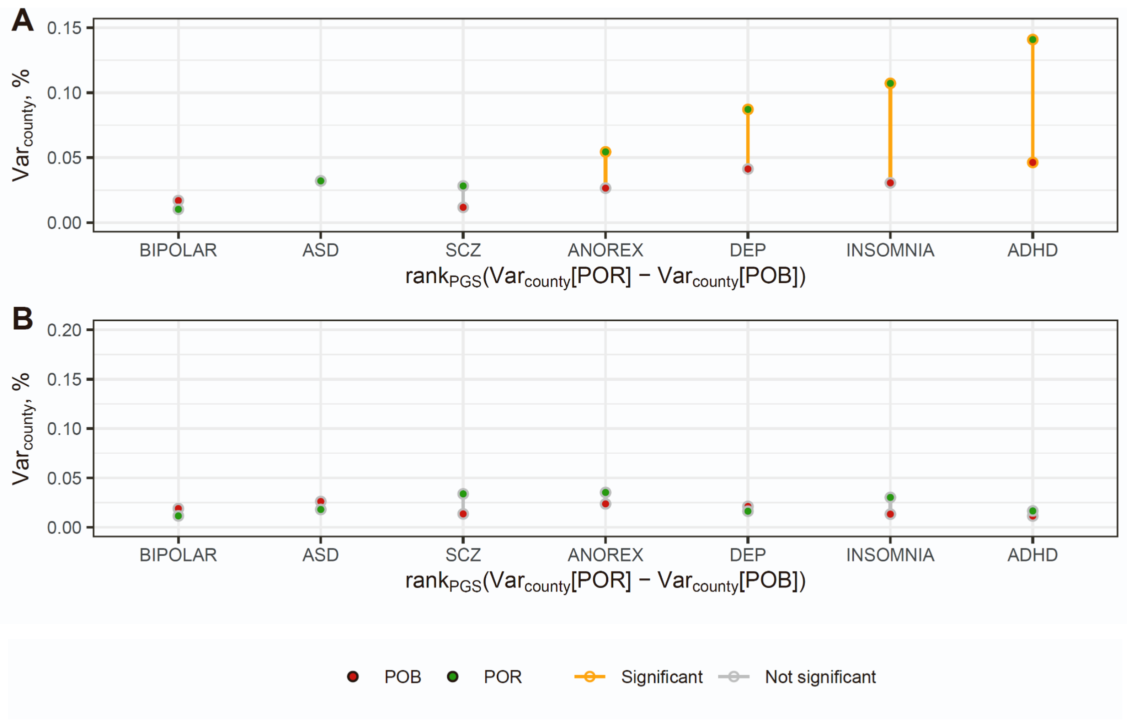

Figure S30. Fraction of the inter-individual variance of (A) PGSs for psychiatric disorders and (B) those PGSs additionally adjusted for PGS_{EA4}, explained by county of birth (POB) and county of residence (POR) among unrelated Estonian participants. "ANOREX" - anorexia nervosa, "ADHD" - attention deficit hyperactivity disorder, "ASD" - autism spectrum disorder, "BIPOLAR" - bipolar disorder, "DEP" - depressive symptoms, "SCZ" - schizophrenia. PGSs are preliminary adjusted for the top 100 PCs and demographic covariates. In panel A the PGSs on the x-axis are ordered according to the difference between POR and POB. In panel B the order is the same as in panel A but without PGS_{EA4}.Var_{county} for PGSs adjusted for the top 100 PCs and demographic covariates. Red and green dots refer to the POB and POR, correspondingly. Estimates significantly different from zero are outlined in yellow. The line connecting the two points is yellow when the variance explained jointly by POB and POR is significantly larger than the variance explained by only the weaker predictor (if significant) or when the stronger predictor is significant. The significance level is 0.05, after Bonferroni correction.

Selective migration and correlations between mate-pair PGSs (Supplemental Note 8)

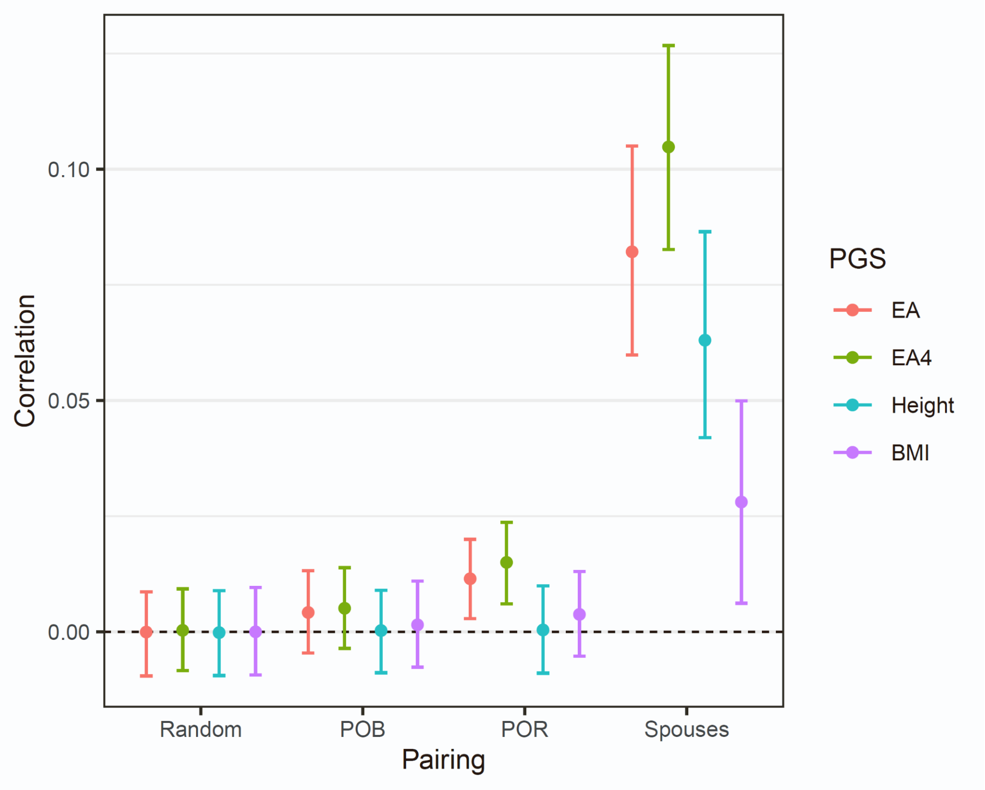

Figure S31. Mating by proximity and assortative mating in Estonia. Correlation between (1) random pairs of individuals from the entire EstBB; (2) random paris of individulas with the same county-level place of birth (POB); (3) random paris of individulas with the same county-level place of residence (POR) and (4) spouses defined as pairs of individuals sharing a child in the EstBB. Error bars correspond to 95% confidence intervals (CIs). The estimates and CIs for the points 1-3 were derived from 1000 random sets of pairs picked according to the described rules. The CIs for spouses were calculated using bootstrap procedure with 1000 replicates.

How large are the regional differences in PGS_{EA}? (Supplemental Note 9)

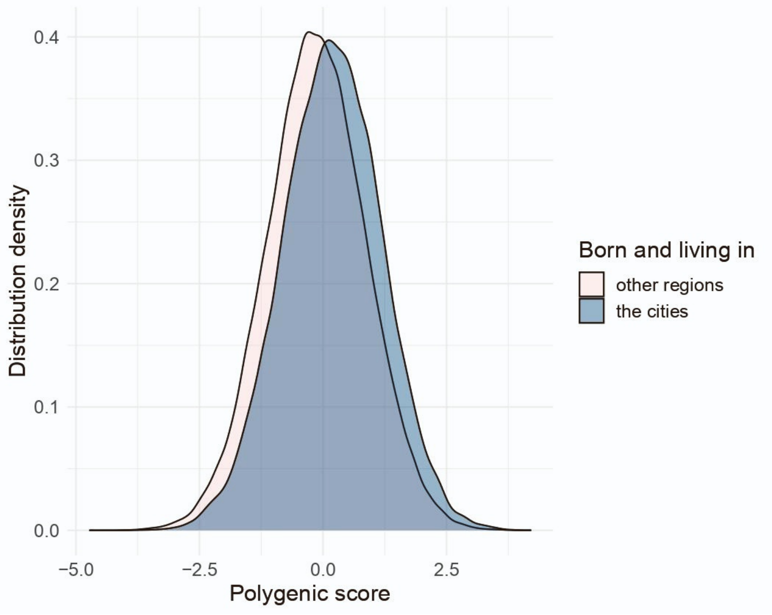

Figure S32. Comparison of the distribution of the polygenic score for educational attainment for individuals born and living in Tallinn and Tartu City versus those born and living in other regions of Estonia (ORE).

Genetic predictors of ORE-to-cities migration

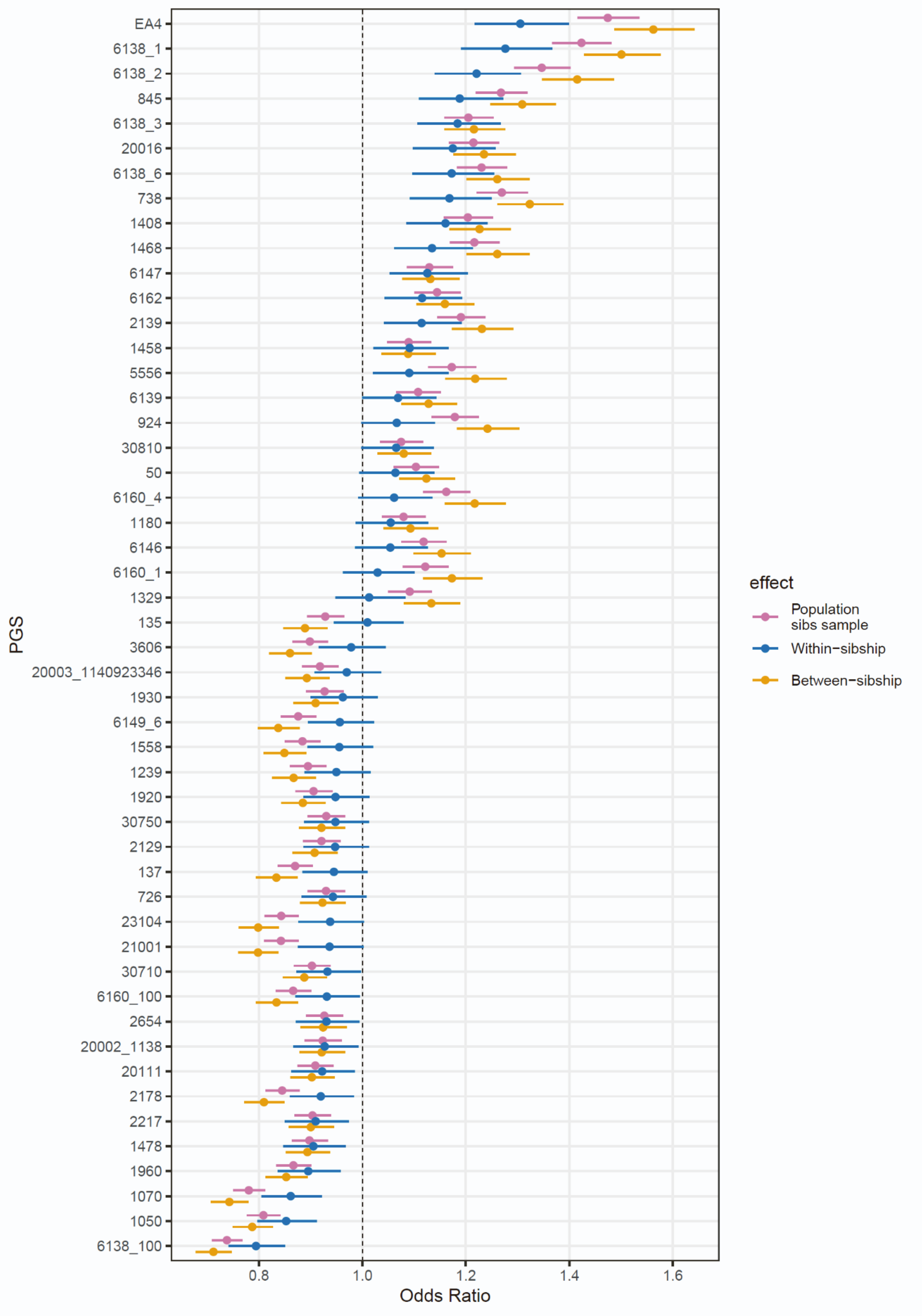

Figure S33. Mixed effects logistic regression results for all the PGSs tested as predictors of migration from ORE to the major cities (Tallinn or Tartu). All the effects are estimated in a sample of

siblings with only siblings born in the same county being included. The estimates are obtained using mixed effects logistic regression with a random intercept for sibship. All PGSs are preliminary adjusted for the top 100 PCs and the demographic covariates. Results are shown for PGSs with a significant population effect after the Bonferroni correction. Vertical dashed line indicates Odds Ratio equal to 1. Error bars correspond to 95% confidence intervals.

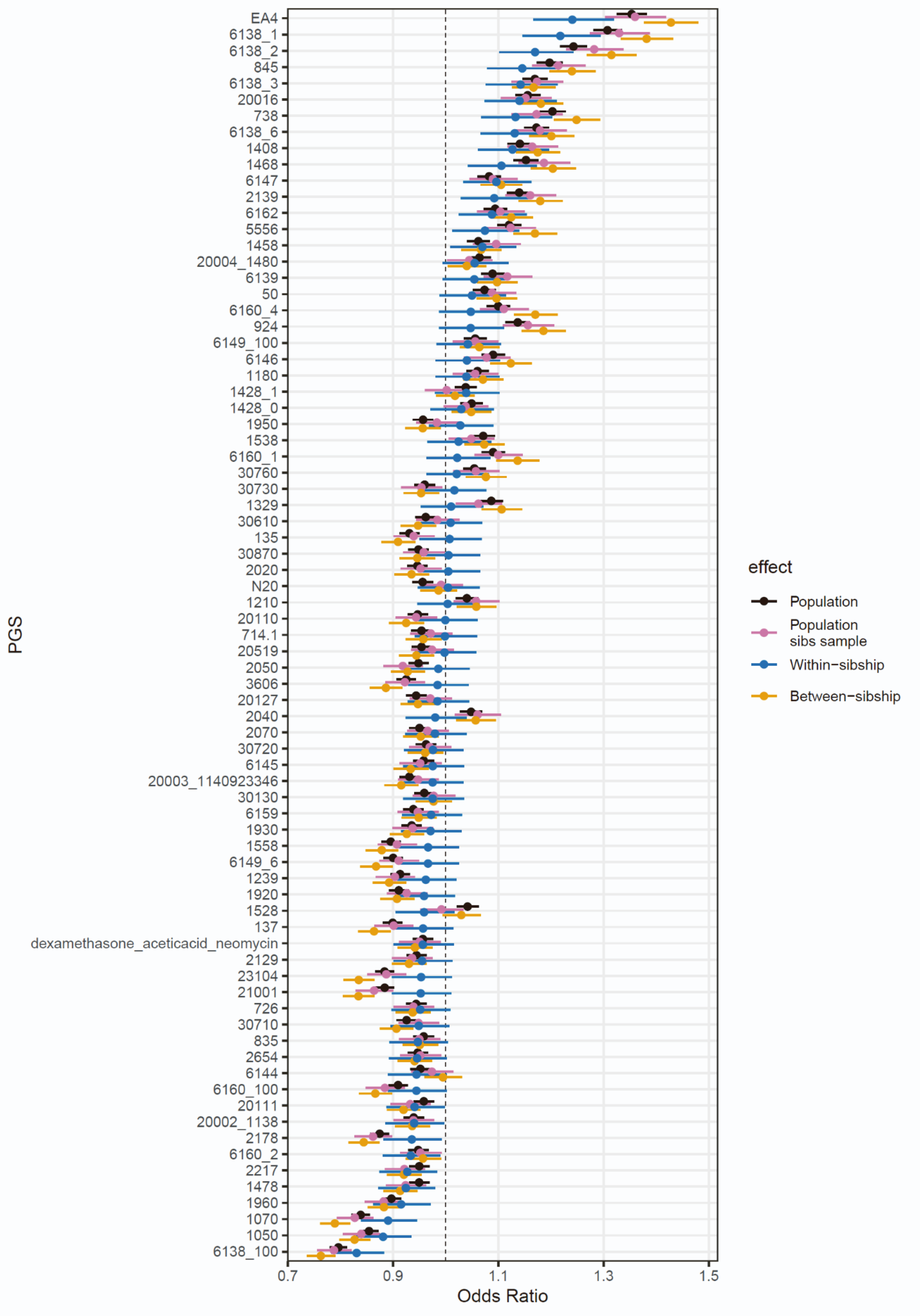

PGS
EA4
6138_1
6138_2
845
6138_3
20016
738
6138_6
1408
1468
6147
2139
6162
5556
1458
20004_1480
6139
50
6160_4
924
6149_100
6146
1180
1428_1
1428_0
1950
1538
6160_1
30760
30730
1329
30610
135
30870
2020
N20
1210
20110
714.1
20519
2050
3606
20127
2040
2070
30720
6145
20003_1140923346
30130
6159
1930
1558
6149_6
1239
1920
1528
137
dexamethasone_aceticacid_neomycin
2129
23104
21001
726
30710
835
2654
6144
6160_100
20111
20002_1138
2178
6160_2
2217
1478
1960
1070
1050
6138_100
0.7
0.9
1.1
1.3
1.5
Odds Ratio
effect
Population
Population sibs sample
Within−sibship
Between−sibship

Figure S34. A fixed effects logistic regression results for all the PGSs tested as predictors of migration from ORE to the major cities (Tallinn or Tartu). Population effects are estimated in the subsample of unrelated Estonians. The other effects are estimated in a sample of siblings with only siblings born in the same county being included. The estimates are obtained using fixed effects logistic regression. All PGSs are preliminary adjusted for the top 100 PCs and the demographic covariates. Results are shown for PGSs with a significant population effect estimated in the subsample of unrelated Estonians after the Bonferroni correction. Vertical dashed line indicates Odds Ratio equal to 1. Error bars correspond to 95% confidence intervals.

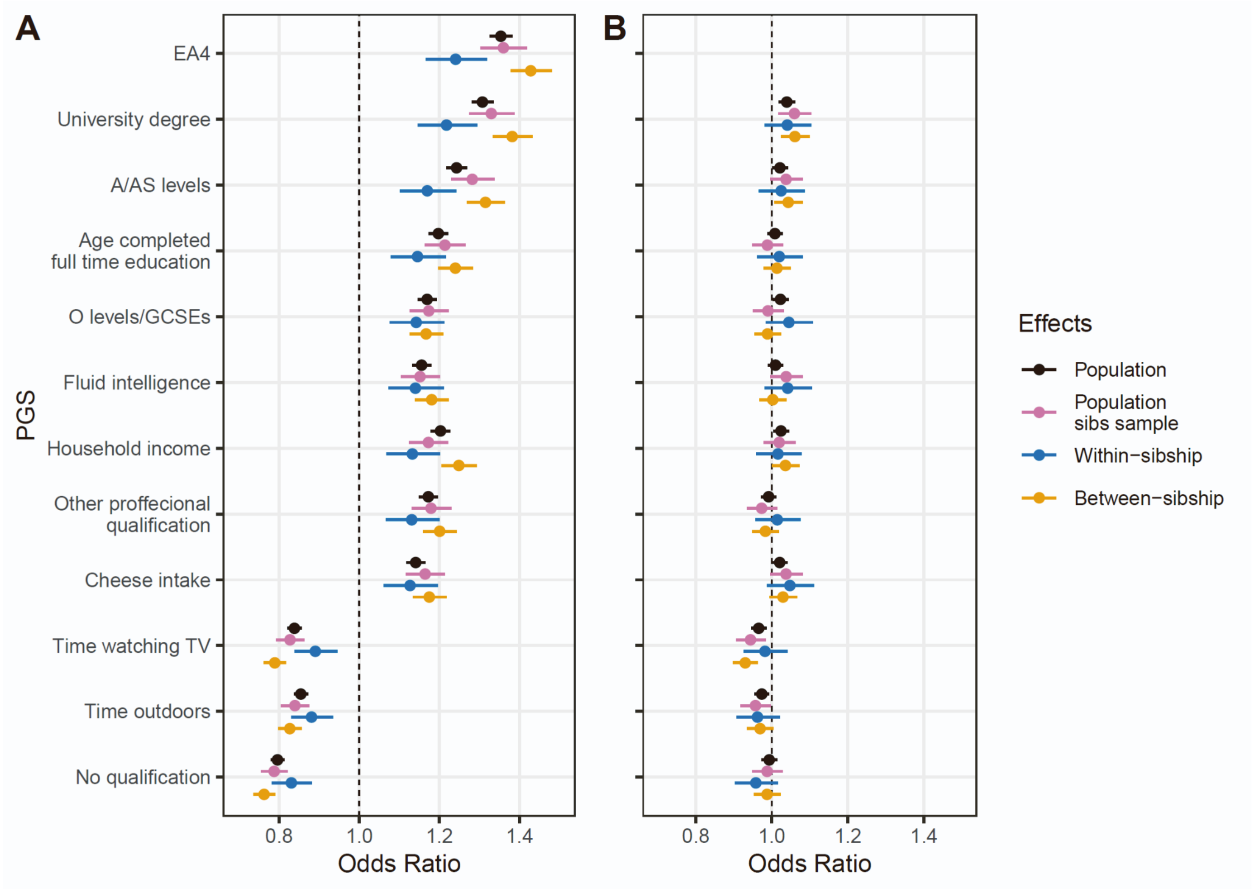

Figure S35. Fixed effects logistic regression results for PGSs with a significant within-sibship effect after the Bonferroni correction in Figure S32, as predictors of migration from ORE to the major cities (Tallinn or Tartu). Population effects are estimated in the subsample of unrelated Estonians. The other effects are estimated in a sample of siblings with only siblings born in the same county being included. (A) Effect sizes for PGSs; (B) Effect sizes of PGSs additionally adjusted for PGS_{EA4}. All PGSs are preliminary adjusted for top 100 PCs and the demographic covariates. Vertical dashed line indicates Odds Ratio equal to 1. Error bars correspond to 95% confidence intervals.

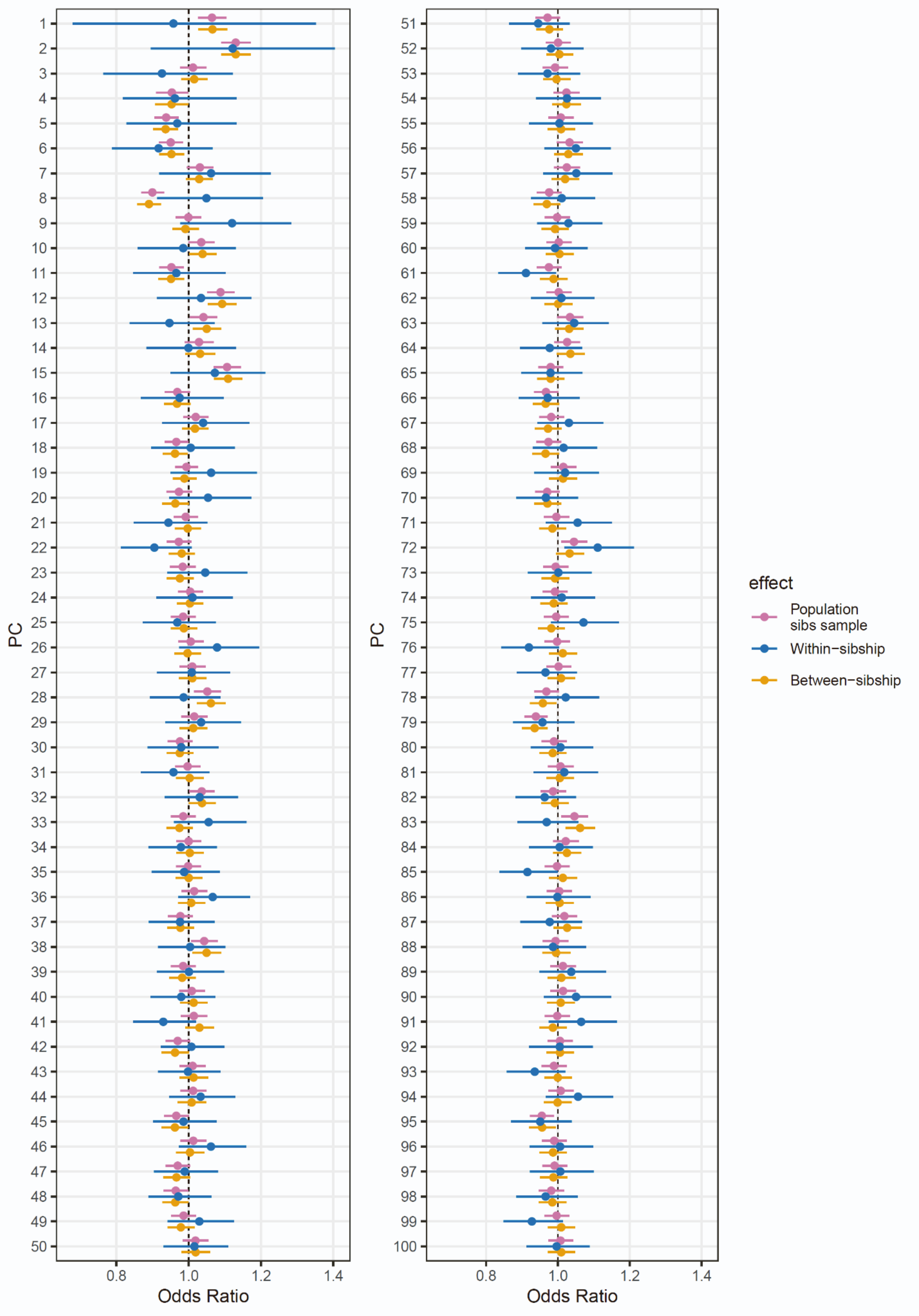
PC
Odds Ratio
0.8
1.0
1.2
1.4
effect
Population sibs sample
Within−sibship
Between−sibship

Figure S36. Mixed effects logistic regression results for the top 100 PCs as predictors of migration from ORE to the major cities (Tallinn or Tartu). All the effects are estimated in a sample of siblings with only siblings born in the same county being included. The estimates are obtained using mixed effects logistic regression with a random intercept for sibship. Vertical dashed line indicates Odds Ratio equal to 1. Error bars correspond to 95% confidence intervals.

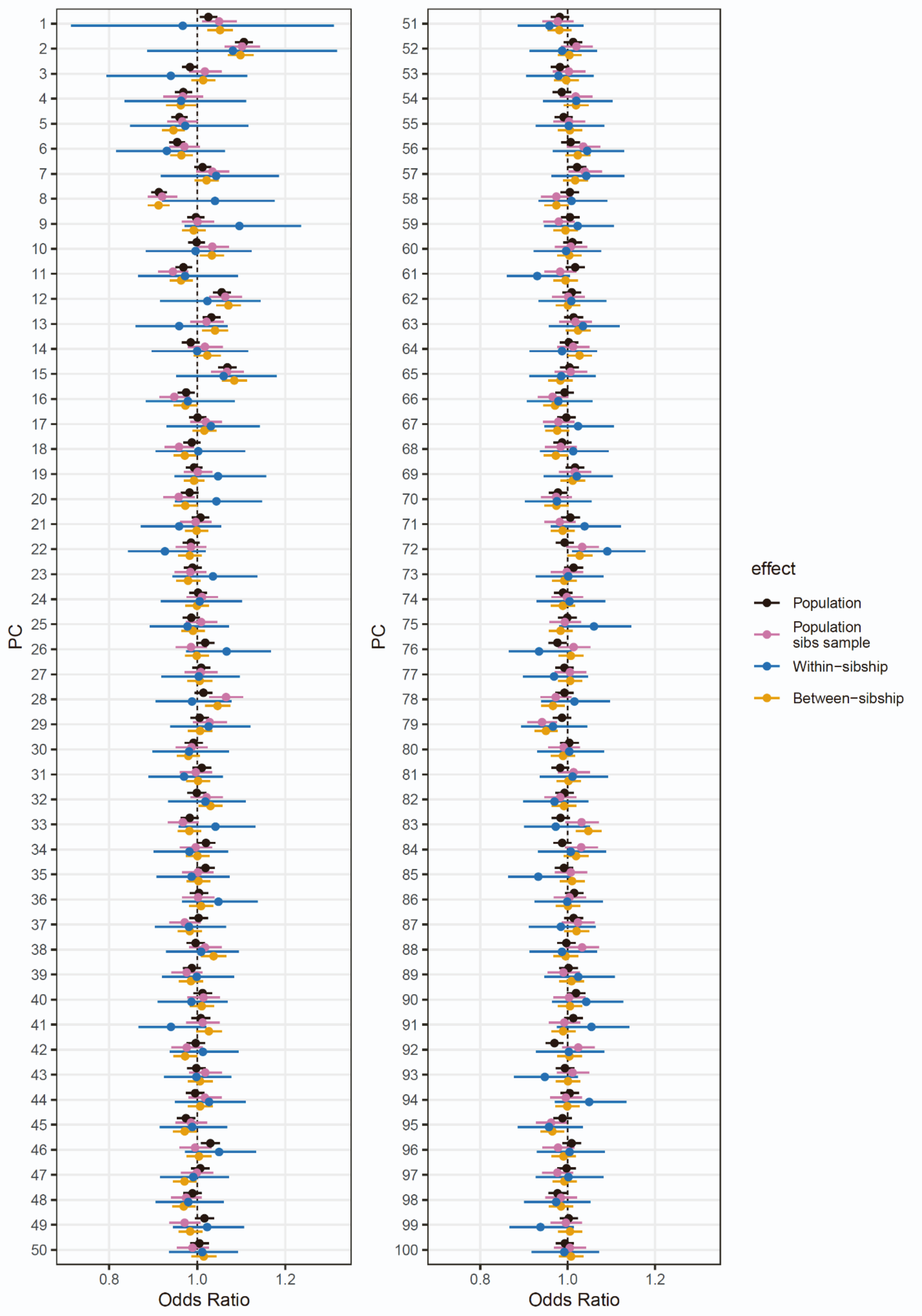
PC
Odds Ratio
0.8
1.0
1.2
effect
Population
Population sibs sample
Within−sibship
Between−sibship

Figure S37. Fixed effects logistic regression results for the top 100 PCs as predictors of migration from ORE to the major cities (Tallinn or Tartu). Population effects are estimated in the subsample of unrelated Estonians. The other effects are estimated in a sample of siblings with only siblings born in the same county being included. The estimates are obtained using fixed effects logistic regression. Vertical dashed line indicates Odds Ratio equal to 1. Error bars correspond to 95% confidence intervals.

Geographical distribution of (s)PGS_{EA}

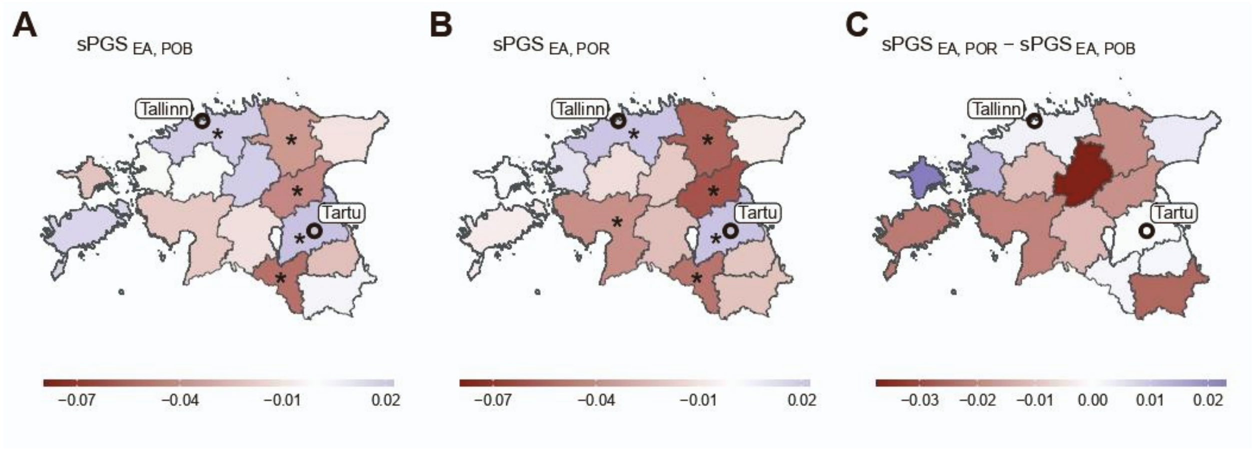

Figure S38. $sPGS_{EA}$ landscape in Estonia among Estonian participants. Mean $sPGS_{EA}$ of individuals (A) born or (B) residing in each county. (C) Differences between values in "B" and "A" panels. PGS_{EA} is adjusted for demographic and genetic ancestry covariates. Counties with sample mean values significantly different from zero after FDR correction at the 0.05 level are marked with an asterisk (*).

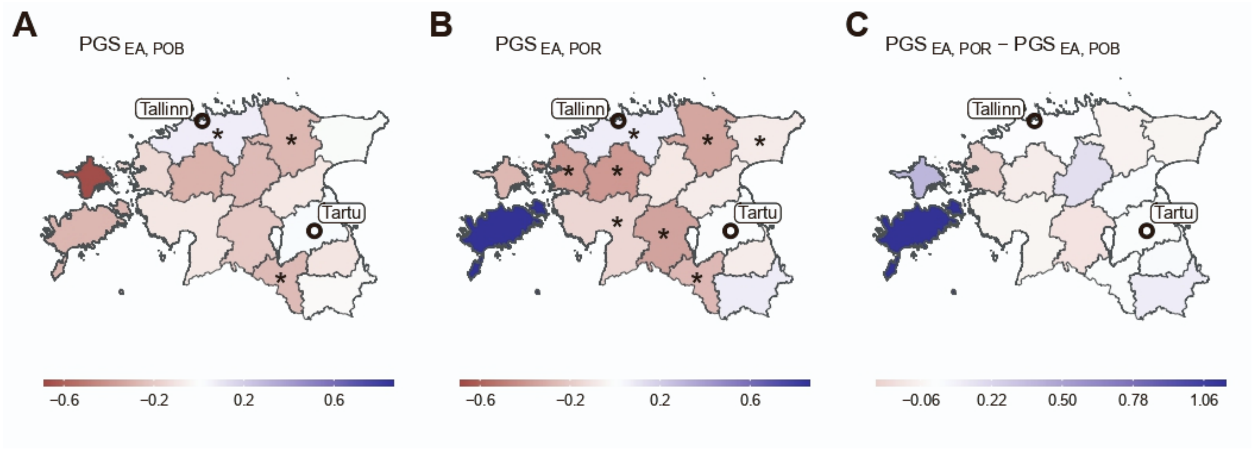

Figure S39. PGS_{EA} landscape in Estonia among Russian participants. Mean PGS_{EA} of individuals (A) born or (B) residing in each county. (C) Differences between values in "B" and "A" panels. PGS_{EA} is adjusted for demographic and genetic ancestry covariates. Counties with sample mean values significantly different from zero after FDR correction at the 0.05 level are marked with an asterisk (*).

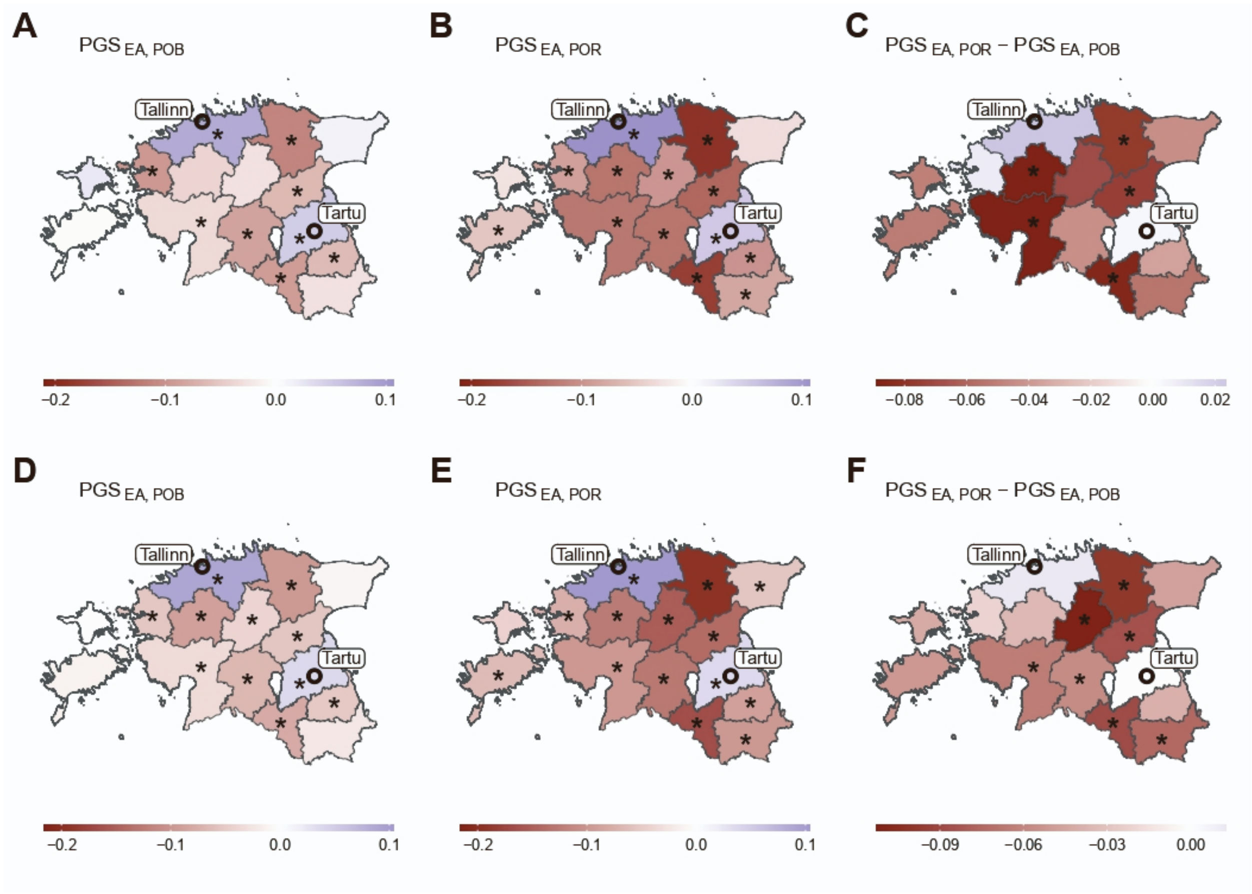

Figure S40. PGS_{EA} landscape in Estonia among (A-C) male and (D-F) female Estonian participants. Mean PGS_{EA} of individuals (A, D) born or (B, E) residing in each county. (C, F) Differences between values in "B" and "A" panels ("E" and "D", correspondingly). PGS_{EA} is adjusted for demographic and genetic ancestry covariates. Counties with sample mean values significantly different from zero after FDR correction at the 0.05 level are marked with an asterisk (*).

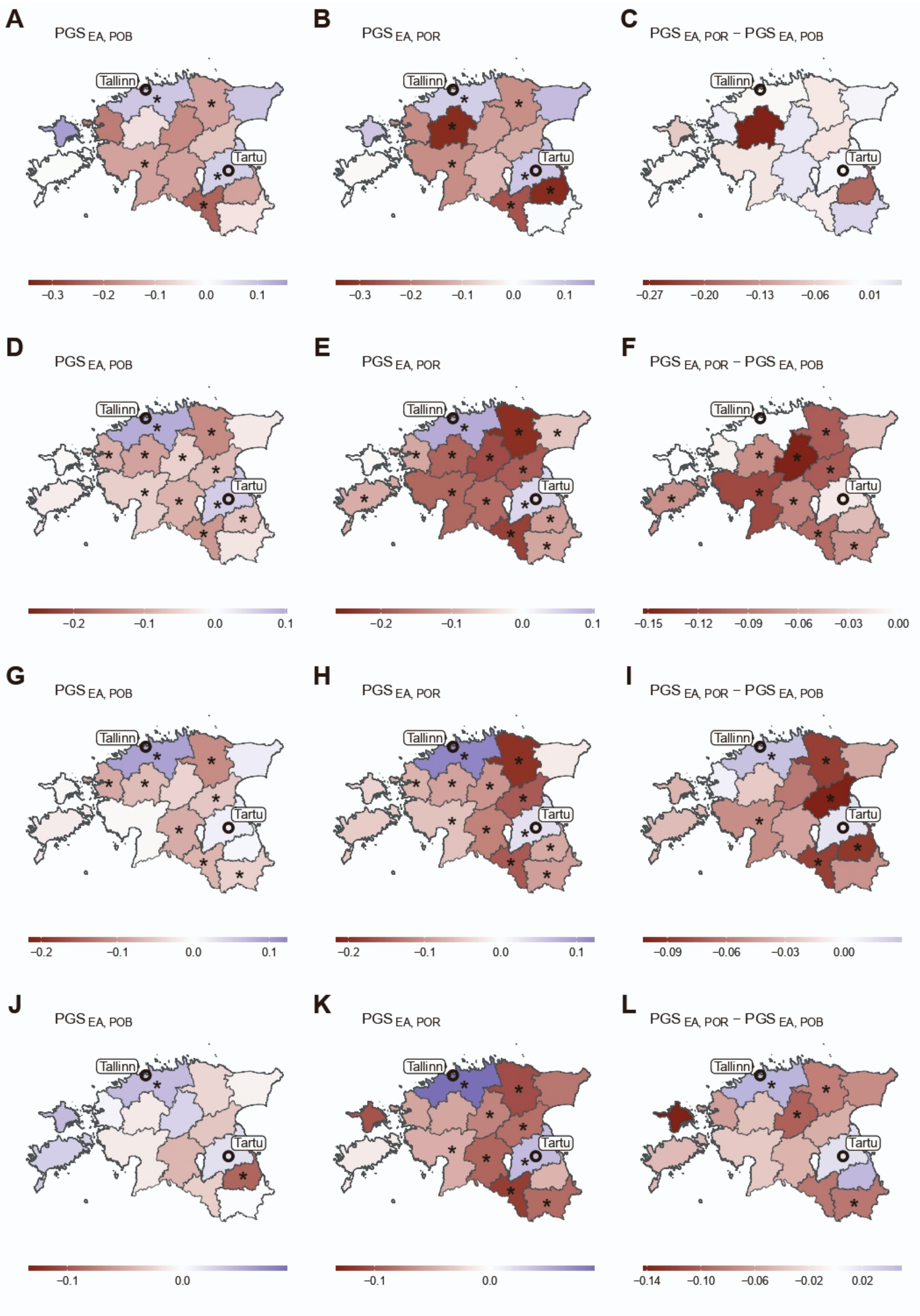

A
PGS EA, POB
Tallinn
Tartu
−0.3 −0.2 −0.1 0.0 0.1
B
PGS EA, POR
Tallinn
Tartu
−0.3 −0.2 −0.1 0.0 0.1
C
PGS EA, POR − PGS EA, POB
Tallinn
Tartu
−0.27 −0.20 −0.13 −0.06 0.01
D
PGS EA, POB
Tallinn
Tartu
−0.2 −0.1 0.0 0.1
E
PGS EA, POR
Tallinn
Tartu
−0.2 −0.1 0.0 0.1
F
PGS EA, POR − PGS EA, POB
Tallinn
Tartu
−0.15 −0.12 −0.09 −0.06 −0.03 0.00
G
PGS EA, POB
Tallinn
Tartu
−0.2 −0.1 0.0 0.1
H
PGS EA, POR
Tallinn
Tartu
−0.2 −0.1 0.0 0.1
I
PGS EA, POR − PGS EA, POB
Tallinn
Tartu
−0.09 −0.06 −0.03 0.00
J
PGS EA, POB
Tallinn
Tartu
−0.1 0.0
K
PGS EA, POR
Tallinn
Tartu
−0.1 0.0
L
PGS EA, POR − PGS EA, POB
Tallinn
Tartu
−0.14 −0.10 −0.06 −0.02 0.02

Figure S41. PGS_{EA} landscape in Estonia among Estonian participants stratified by age. Age groups were defined as (A-C) 18-24, (D-F) 25-48, (G-I) 49-64, (J-L) 65+. Mean PGS_{EA} of individuals (A, D, G, J) born or (B, E, H, K) residing in each county. (C, F, I, L) Differences between values in "B" and "A" panels ("E"-"D", "H"-"G", "K"-"J" correspondingly). PGS_{EA} is adjusted for demographic and genetic ancestry covariates. Counties with sample mean values significantly different from zero after FDR correction at the 0.05 level are marked with an asterisk (*).

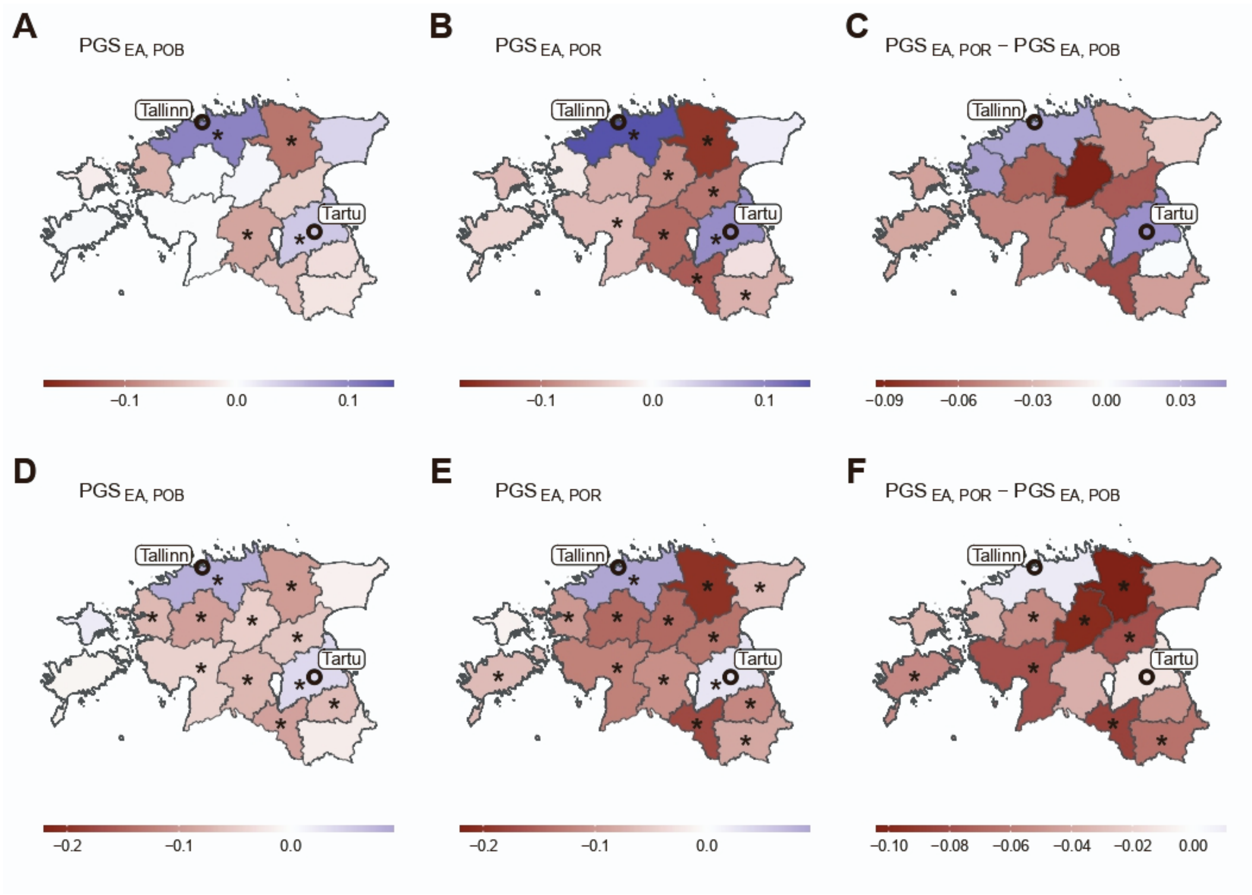

Figure S42. PGS_{EA} landscape in Estonia among Estonian participants stratified by year of joining the biobank. The periods of joining are (A) 2001-2016 and (B) 2017-2021. Mean PGS_{EA} of individuals (A, D) born or (B, E) residing in each county. (C, F) Differences between values in "B" and "A" panels ("E" and "D", correspondingly). PGS_{EA} is adjusted for demographic and genetic ancestry covariates. Counties with sample mean values significantly different from zero after FDR correction at the 0.05 level are marked with an asterisk (*).

Geographical distribution of educational attainment phenotype

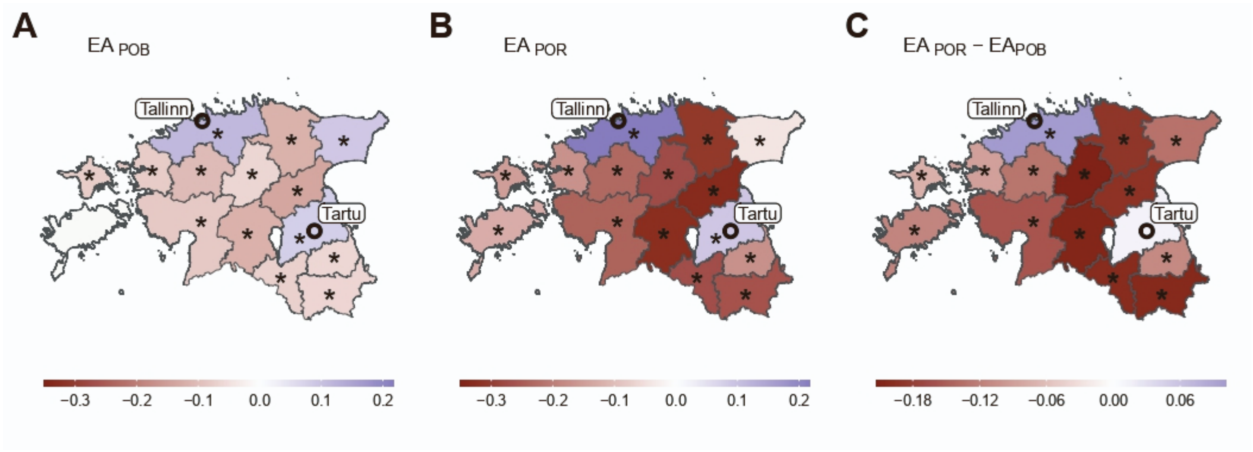

Figure S43. EA (years of education) landscape in Estonia among Estonian participants. Mean EA of individuals (A) born or (B) residing in each county. (C) Differences between values in "B" and "A" panels. EA is adjusted for demographic and genetic ancestry covariates. Counties with sample mean values significantly different from zero after FDR correction at the 0.05 level are marked with an asterisk (*).

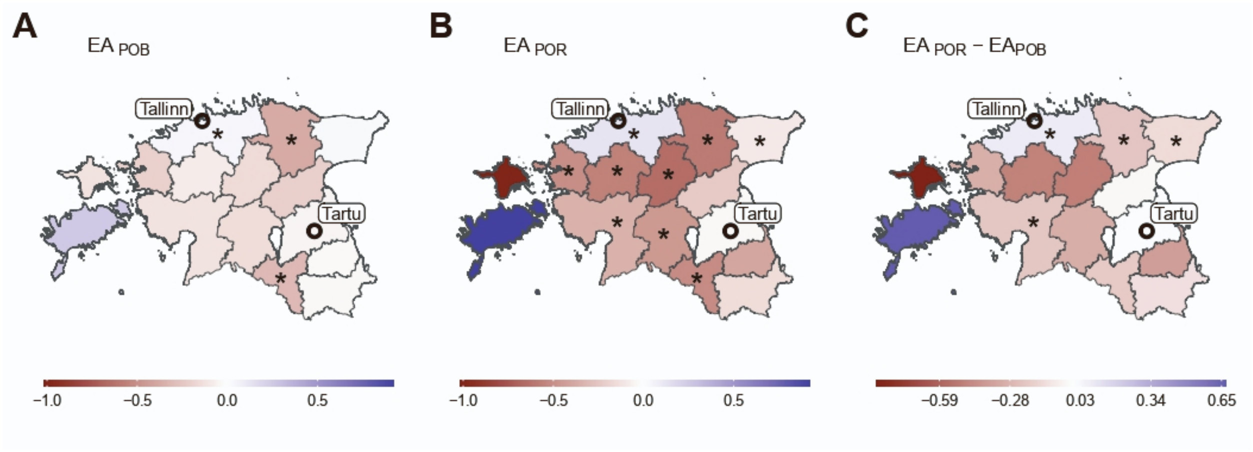

Figure S44. EA (years of education) landscape in Estonia among Russian participants. Mean EA of individuals (A) born or (B) residing in each county. (C) Differences between values in "B" and "A" panels. EA is adjusted for demographic and genetic ancestry covariates. Counties with sample mean values significantly different from zero after FDR correction at the 0.05 level are marked with an asterisk (*).

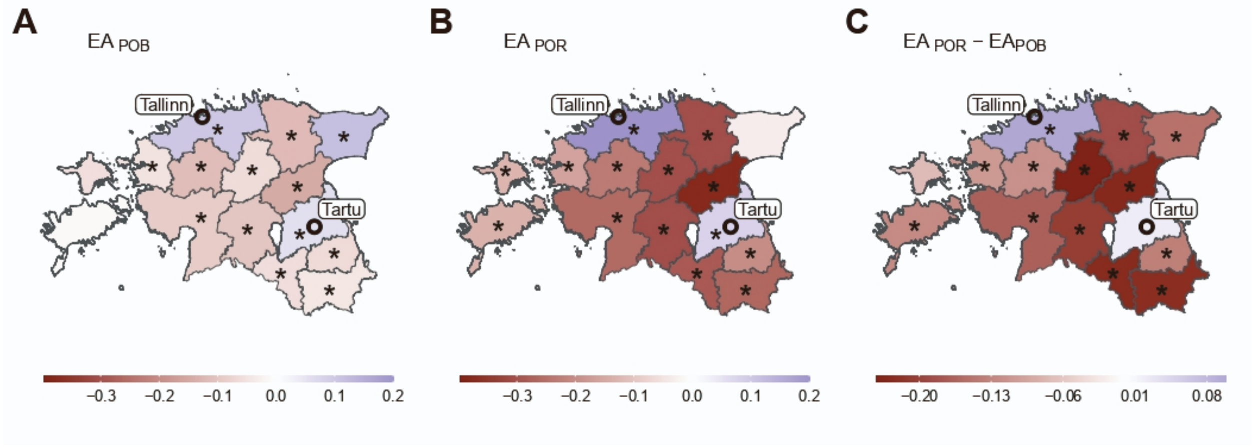

Figure S45. EA (years of education) landscape in Estonia among unrelated Estonian participants. Mean EA of individuals (A) born or (B) residing in each county. (C) Differences between values in "B" and "A" panels. EA is adjusted for demographic and genetic ancestry covariates. Counties with sample mean values significantly different from zero after FDR correction at the 0.05 level are marked with an asterisk (*).

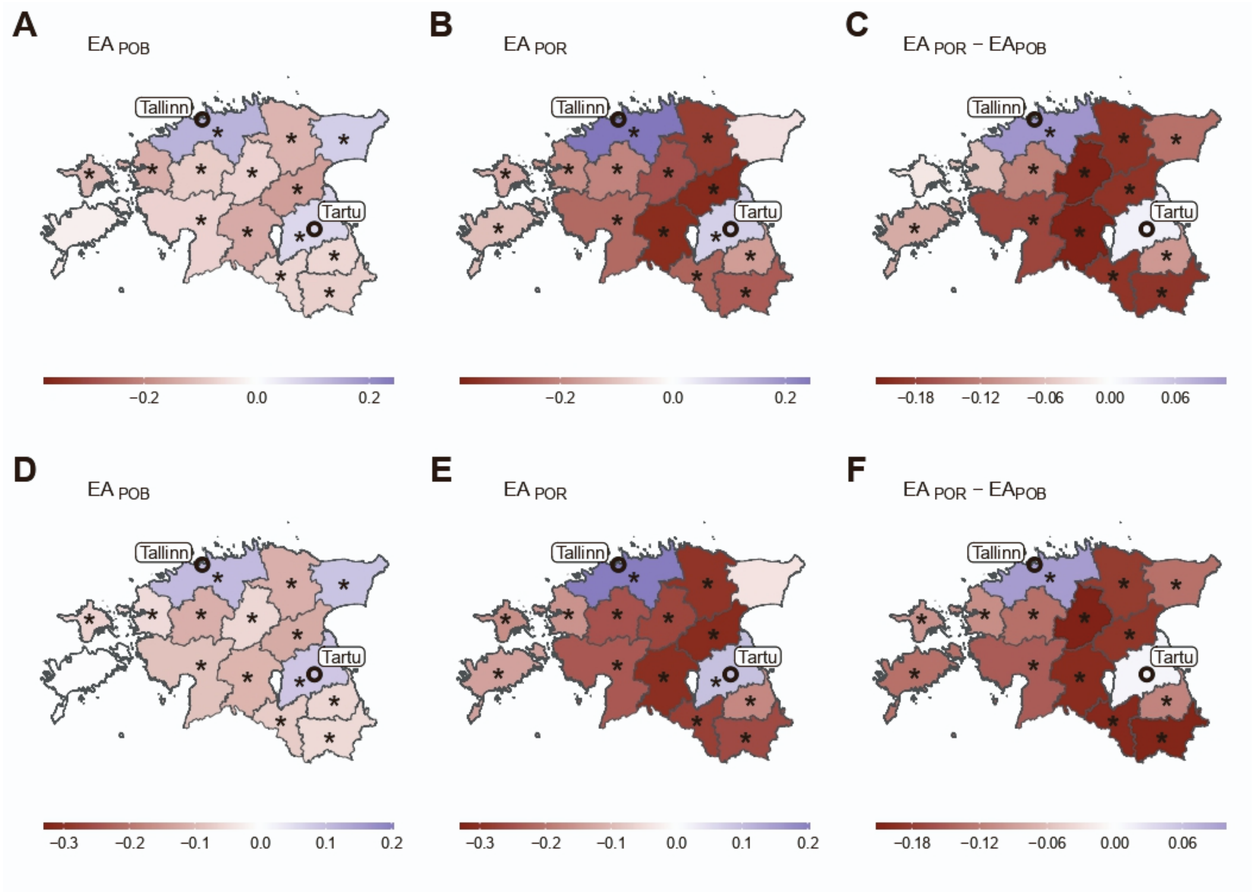

Figure S46. EA (years of education) landscape in Estonia among (A-C) male and (D-F) female Estonian participants. Mean EA of individuals (A, D) born or (B, E) residing in each county. (C, F) Differences between values in "B" and "A" panels ("E" and "D", correspondingly). EA is adjusted for demographic and genetic ancestry covariates. Counties with sample mean values significantly different from zero after FDR correction at the 0.05 level are marked with an asterisk (*).

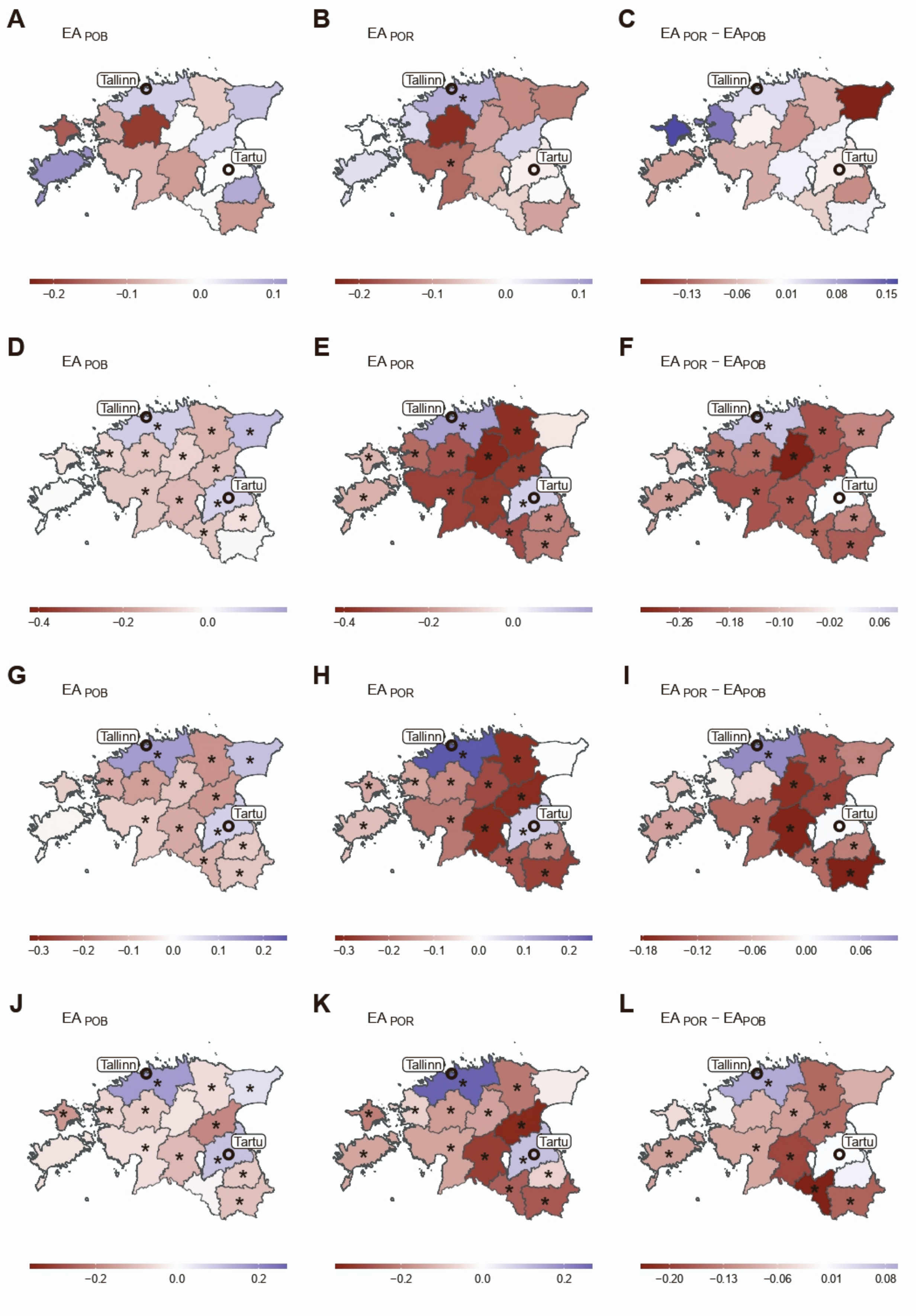
A
EA POB
Tallinn
Tartu
−0.2 −0.1 0.0 0.1
B
EA POR
Tallinn
Tartu
−0.2 −0.1 0.0 0.1
C
EA POR − EAPOB
Tallinn
Tartu
−0.13 −0.06 0.01 0.08 0.15
D
EA POB
Tallinn
Tartu
−0.4 −0.2 0.0
E
EA POR
Tallinn
Tartu
−0.4 −0.2 0.0
F
EA POR − EAPOB
Tallinn
Tartu
−0.26 −0.18 −0.10 −0.02 0.06
G
EA POB
Tallinn
Tartu
−0.3 −0.2 −0.1 0.0 0.1 0.2
H
EA POR
Tallinn
Tartu
−0.3 −0.2 −0.1 0.0 0.1 0.2
I
EA POR − EAPOB
Tallinn
Tartu
−0.18 −0.12 −0.06 0.00 0.06
J
EA POB
Tallinn
Tartu
−0.2 0.0 0.2
K
EA POR
Tallinn
Tartu
−0.2 0.0 0.2
L
EA POR − EAPOB
Tallinn
Tartu
−0.20 −0.13 −0.06 0.01 0.08

Figure S47. EA (years of education) landscape in Estonia among Estonian participants stratified by age. Age groups were defined as (A-C) 18-24, (D-F) 25-48, (G-I) 49-64, (J-L) 65+. Mean EA of individuals (A, D, G, J) born or (B, E, H, K) residing in each county. (C, F, I, L) Differences between values in "B" and "A" panels ("E"-"D", "H"-"G", "K"-"J" correspondingly). EA is adjusted for demographic and genetic ancestry covariates. Counties with sample mean values significantly different from zero after FDR correction at the 0.05 level are marked with an asterisk (*).

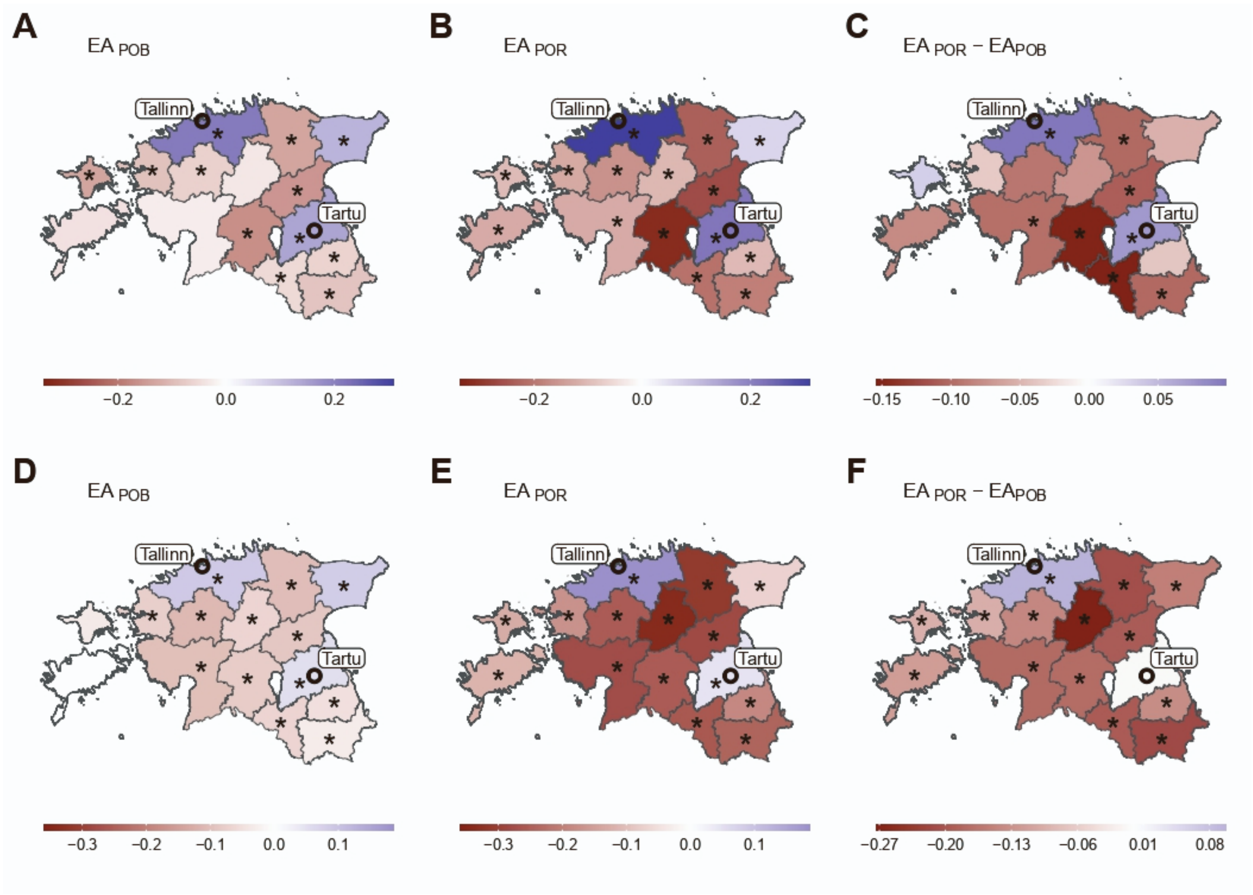

Figure S48. EA (years of education) landscape in Estonia among Estonian participants stratified by year of joining the biobank. The periods of joining are (A-C) 2001-2016 and (D-F) 2017-2021. Mean EA of individuals (A, D) born or (B, E) residing in each county. (C, F) Differences between values in "B" and "A" panels ("E" and "D", correspondingly). EA is adjusted for demographic and genetic ancestry covariates. Counties with sample mean values significantly different from zero after FDR correction at the 0.05 level are marked with an asterisk (*).

(s)PGS$_{EA}$ values in groups with different migration profiles

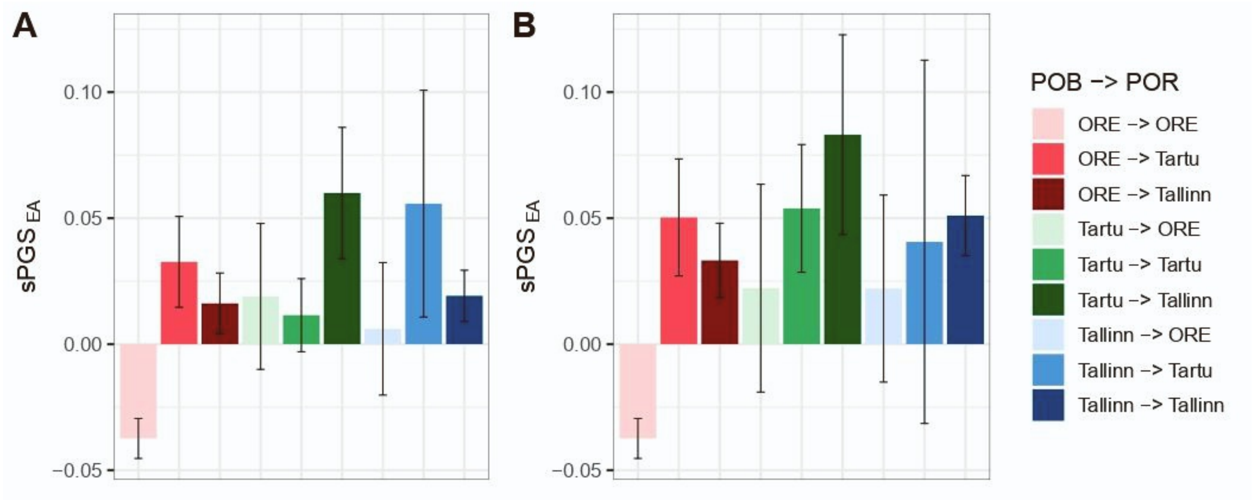

Figure S49. sPGS$_{EA}$ in migration groups among Estonian participants by region of birth (POB) and residence (POR). (A) County-based analysis where POB and POR refer to Tartu County ("Tartu"), Harju County ("Tallinn") and other counties ("ORE"). (B) City-based analysis, where POB and POR refer to Tartu City ("Tartu"), Tallinn ("Tallinn") and other counties ("ORE"). sPGS$_{EA}$ is adjusted for demographic and genetic ancestry covariates. Error bars correspond to 95% confidence intervals.

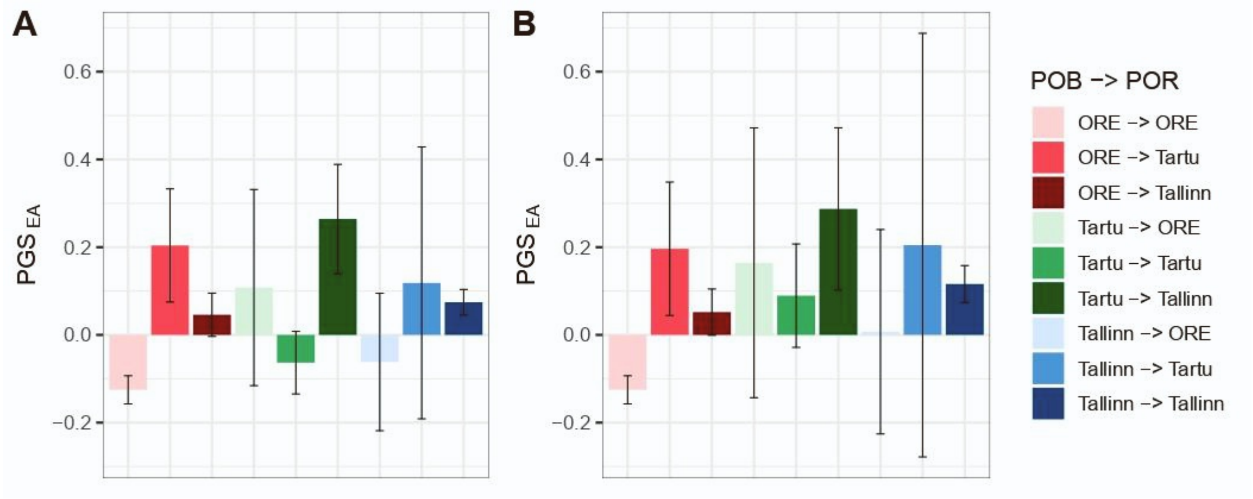

Figure S50. PGS_{EA} in migration groups among Russian participants by region of birth (POB) and residence (POR). (A) County-based analysis where POB and POR refer to Tartu County ("Tartu"), Harju County ("Tallinn") and other counties ("ORE"). (B) City-based analysis, where POB and POR refer to Tartu City ("Tartu"), Tallinn ("Tallinn") and other counties ("ORE"). PGS_{EA} is adjusted for demographic and genetic ancestry covariates. Error bars correspond to 95% confidence intervals.

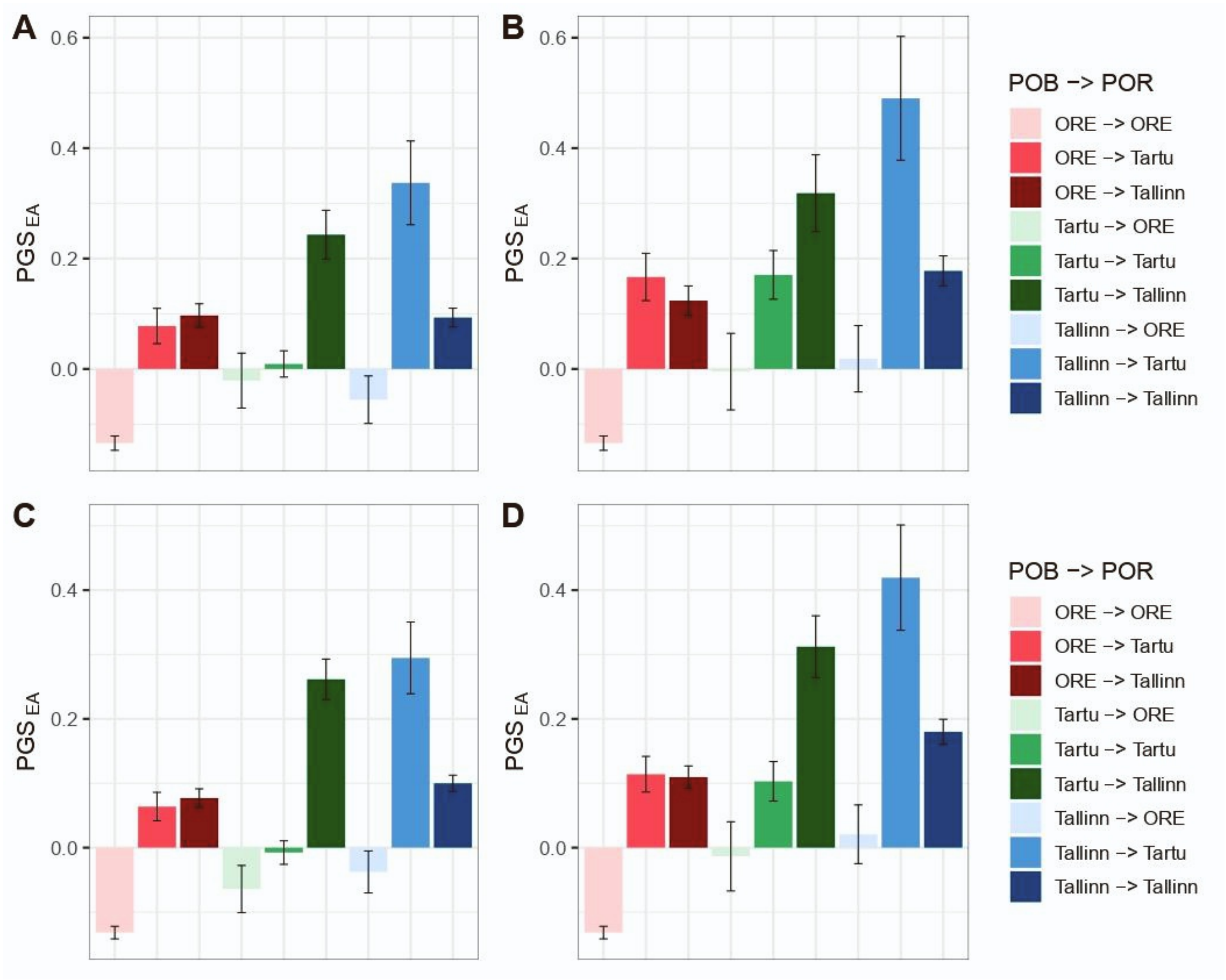

Figure S51. PGS_{EA} in migration groups among (A-B) male and (C-D) female Estonian participants by region of birth (POB) and residence (POR). (A, C) County-based analysis where POB and POR refer to Tartu County ("Tartu"), Harju County ("Tallinn") and other counties ("ORE"). (B, D) City-based analysis, where POB and POR refer to Tartu City ("Tartu"), Tallinn ("Tallinn") and other counties ("ORE"). PGS_{EA} is adjusted for demographic and genetic ancestry covariates. Error bars correspond to 95% confidence intervals.

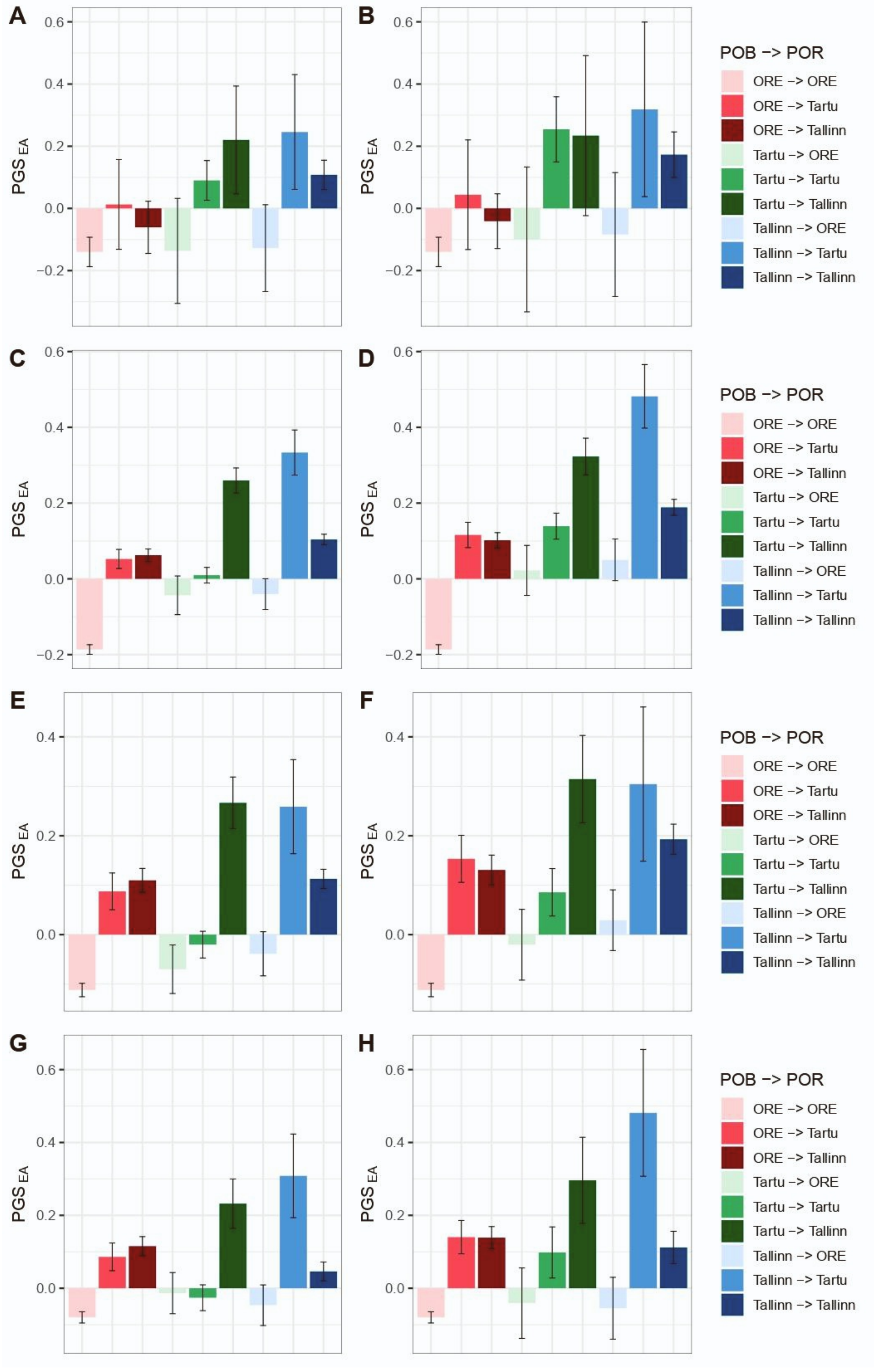
A
B
C
D
E
F
G
H
PGS EA
0.6
0.4
0.2
0.0
−0.2
POB −> POR
ORE −> ORE
ORE −> Tartu
ORE −> Tallinn
Tartu −> ORE
Tartu −> Tartu
Tartu −> Tallinn
Tallinn −> ORE
Tallinn −> Tartu
Tallinn −> Tallinn

Figure S52. PGS_{EA} in migration groups among Estonian participants stratified by age by region of birth (POB) and residence (POR). Age groups were defined as (A-B) 18-24, (C-D) 25-48, (E-F) 49-64, (G-H) 65+. (A, C, E, G) County-based analysis where POB and POR refer to Tartu County ("Tartu"), Harju County ("Tallinn") and other counties ("ORE"). (B, D, F, H) City-based analysis, where POB and POR refer to Tartu City ("Tartu"), Tallinn ("Tallinn") and other counties ("ORE"). PGS_{EA} is adjusted for demographic and genetic ancestry covariates. Error bars correspond to 95% confidence intervals.

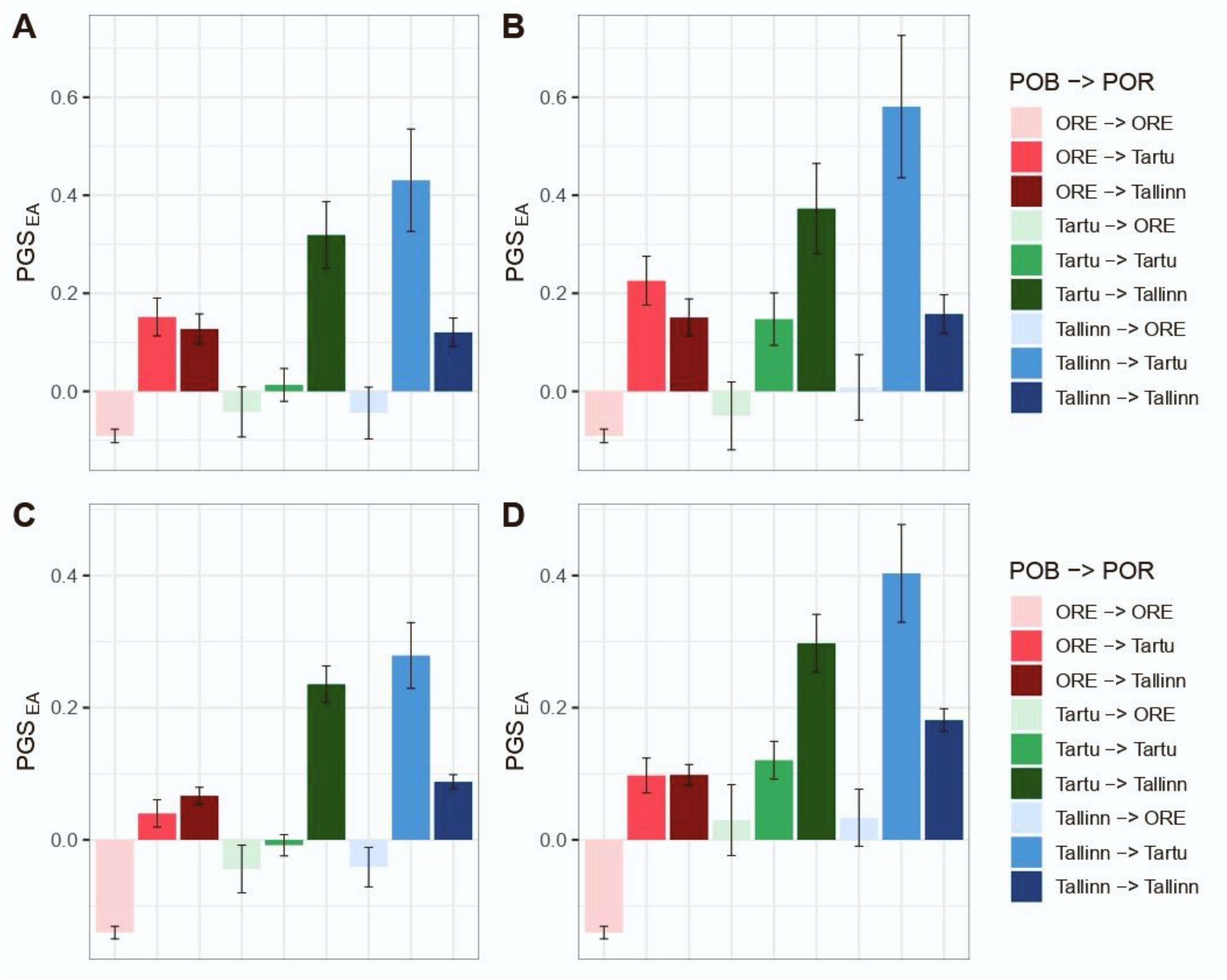

Figure S53. PGS_{EA} in migration groups among Estonian participants stratified by year of joining the biobank by region of birth (POB) and residence (POR). The periods of joining are (A-B) 2001-2016 and (C-D) 2017-2021. (A, C) County-based analysis where POB and POR refer to Tartu County ("Tartu"), Harju County ("Tallinn") and other counties ("ORE"). (B, D) City-based analysis, where POB and POR refer to Tartu City ("Tartu"), Tallinn ("Tallinn") and other counties ("ORE"). PGS_{EA} is adjusted for demographic and genetic ancestry covariates. Error bars correspond to 95% confidence intervals.

Migration direction and (s)PGS_{EA}

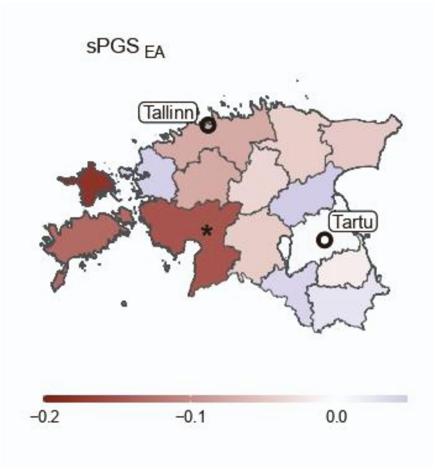

Figure S54. The contrast in mean $sPGS_{EA}$ between residents of Tallinn and Tartu City among Estonian participants by county of birth. The value for each county corresponds to the mean $sPGS_{EA}$ of individuals born in that county and living in Tartu City subtracted from the mean $sPGS_{EA}$ of individuals born in the same county and living in Tallinn. Individuals born in Tallinn or Tartu City are excluded from the analysis. Counties with significant differences between the migrant groups after FDR correction at level 0.05 are marked with an asterisk (*).

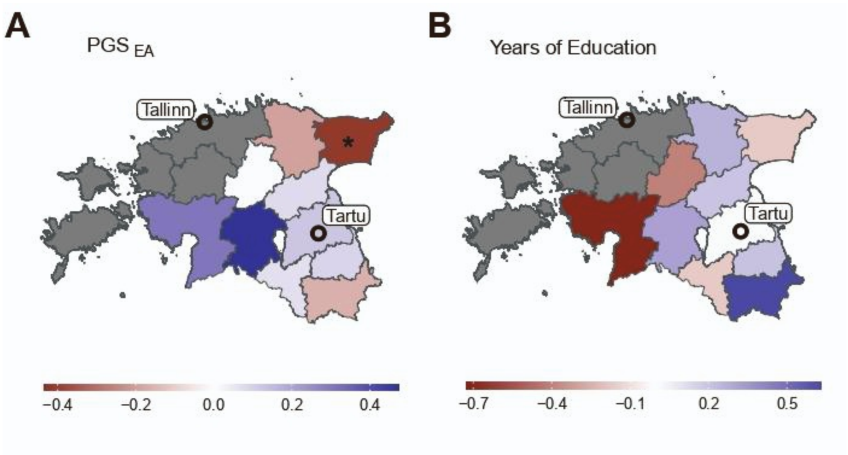

Figure S55. The contrast in mean PGS_{EA} and EA (years of education) between residents of Tallinn and Tartu City among Russian participants by county of birth. (A) The value for each county corresponds to the mean PGS_{EA} of individuals born in that county and living in Tartu City subtracted from the mean PGS_{EA} of individuals born in the same county and living in Tallinn. Individuals born in Tallinn or Tartu City are excluded from the analysis. (B) The same but for the "years of education" phenotype. Counties with significant differences between the migrant groups after FDR correction at level 0.05 are marked with an asterisk (*). The grey colour means there are fewer than two migrants to Tallinn or Tartu City from the corresponding region in the dataset.

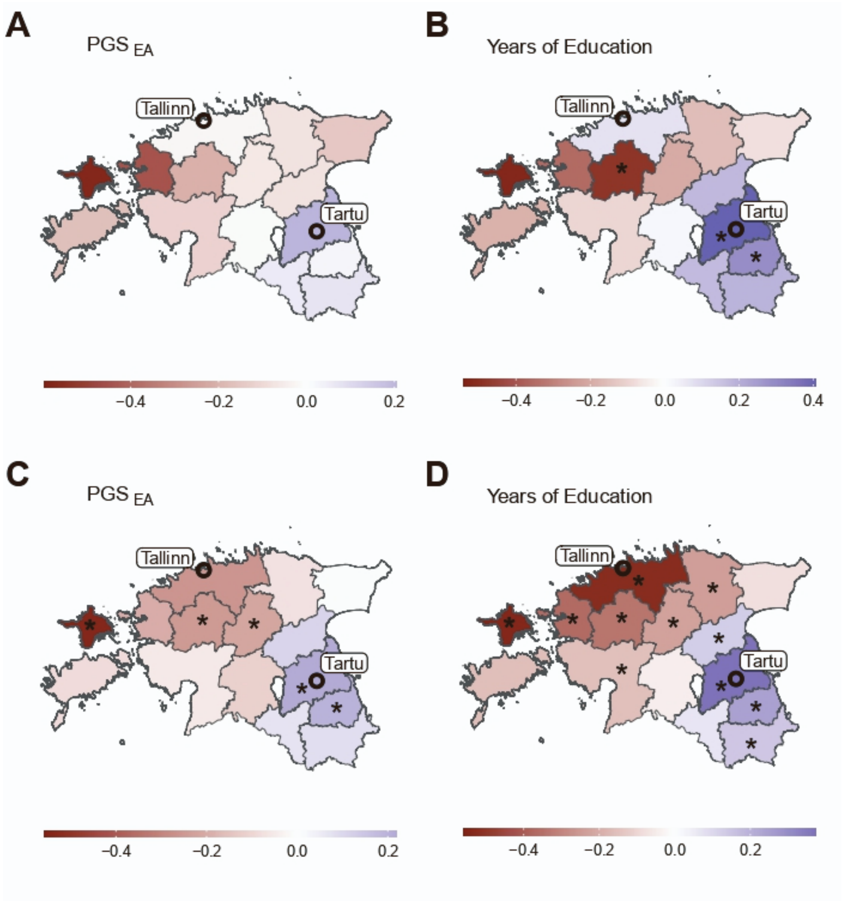

Figure S56. The contrast in mean PGS_{EA} and EA (years of education) between residents of Tallinn and Tartu City among (A-B) male and (C-D) female Estonian participants by county of birth. (A, C) The value for each county corresponds to the mean PGS_{EA} of individuals born in that county and living in Tartu City subtracted from the mean PGS_{EA} of individuals born in the same county and living in Tallinn. Individuals born in Tallinn or Tartu City are excluded from the analysis. (B, D) The same but for the "years of education" phenotype. Counties with significant differences between the migrant groups after FDR correction at level 0.05 are marked with an asterisk (*).

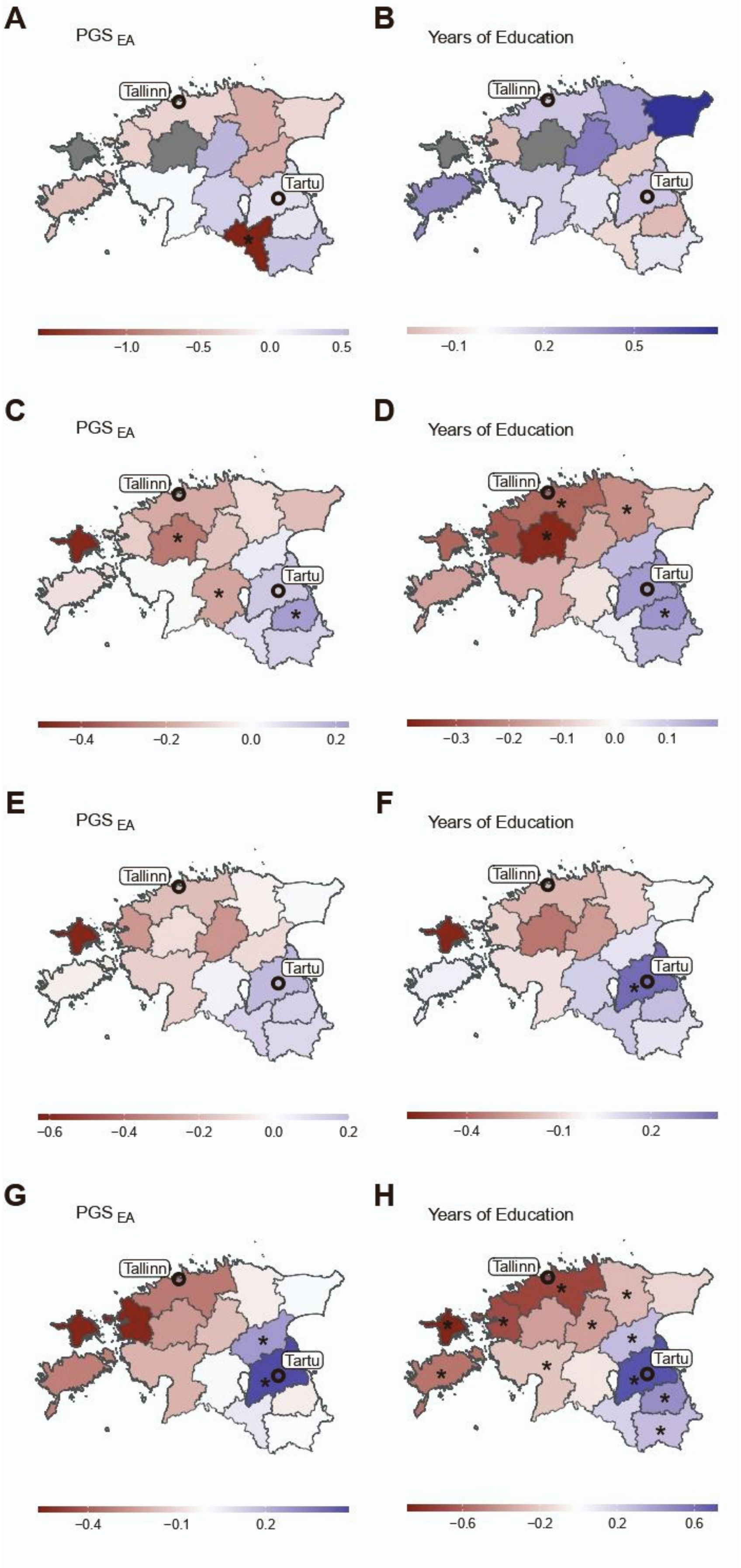
A
PGS EA
Tallinn
Tartu
−1.0
−0.5
0.0
0.5
B
Years of Education
Tallinn
Tartu
−0.1
0.2
0.5
C
PGS EA
Tallinn
Tartu
−0.4
−0.2
0.0
0.2
D
Years of Education
Tallinn
Tartu
−0.3
−0.2
−0.1
0.0
0.1
E
PGS EA
Tallinn
Tartu
−0.6
−0.4
−0.2
0.0
0.2
F
Years of Education
Tallinn
Tartu
−0.4
−0.1
0.2
G
PGS EA
Tallinn
Tartu
−0.4
−0.1
0.2
H
Years of Education
Tallinn
Tartu
−0.6
−0.2
0.2
0.6

Figure S57. The contrast in mean PGS_{EA} and EA (years of education) between residents of Tallinn and Tartu City among Estonian participants stratified by age by county of birth. Age groups were defined as (A-B) 18-24, (C-D) 25-48, (E-F) 49-64, (G-H) 65+. (A, C, E, G) The value for each county corresponds to the mean PGS_{EA} of individuals born in that county and living in Tartu City subtracted from the mean PGS_{EA} of individuals born in the same county and living in Tallinn. Individuals born in Tallinn or Tartu City are excluded from the analysis. (B, D, F, H) The same but for the "years of education" phenotype. Counties with significant differences between the migrant groups after FDR correction at level 0.05 are marked with an asterisk (*). The grey colour means there are fewer than two migrants to Tartu City or Tallinn from the corresponding region in the dataset.

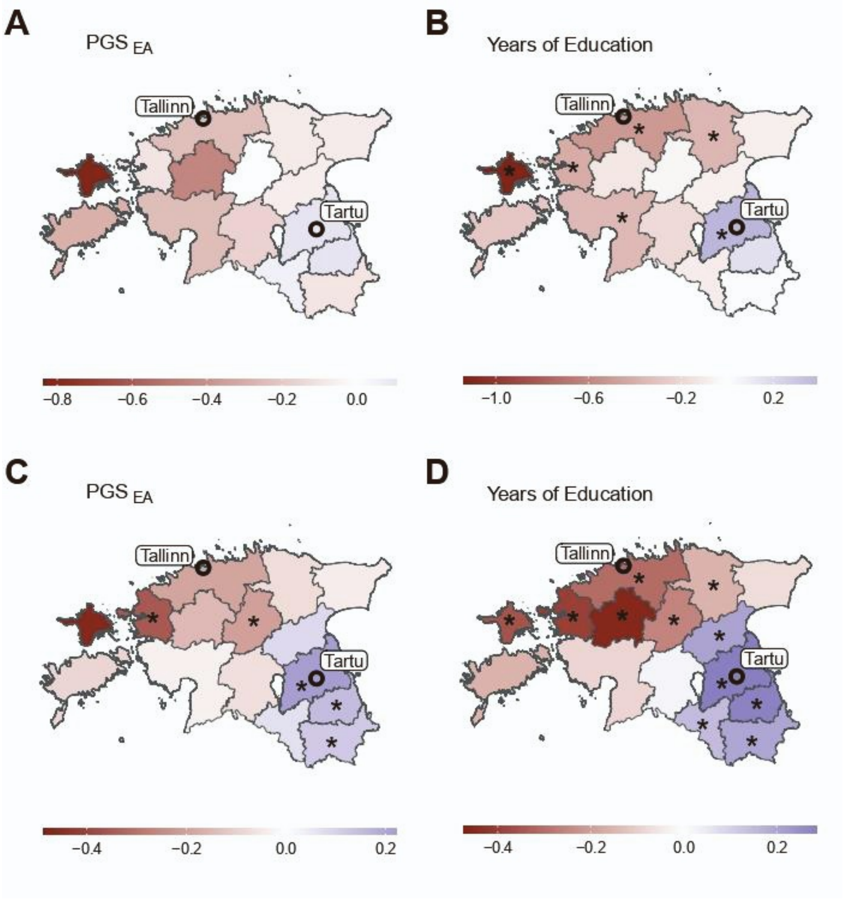

Figure S58. The contrast in mean PGS_{EA} and EA (years of education) between residents of Tallinn and Tartu among Estonian participants stratified by year of joining the biobank by county of birth. The periods of joining are (A-B) 2001-2016 and (C-D) 2017-2021. (A, C) The value for each county corresponds to the mean PGS_{EA} of individuals born in that county and living in Tartu City subtracted from the mean PGS_{EA} of individuals born in the same county and living in Tallinn. Individuals born in Tallinn or Tartu City are excluded from the analysis. (B, D) The same but for the "years of education" phenotype. Counties with significant differences between the migrant groups after FDR correction at level 0.05 are marked with an asterisk (*).

Educational attainment phenotype in groups with different migration profiles

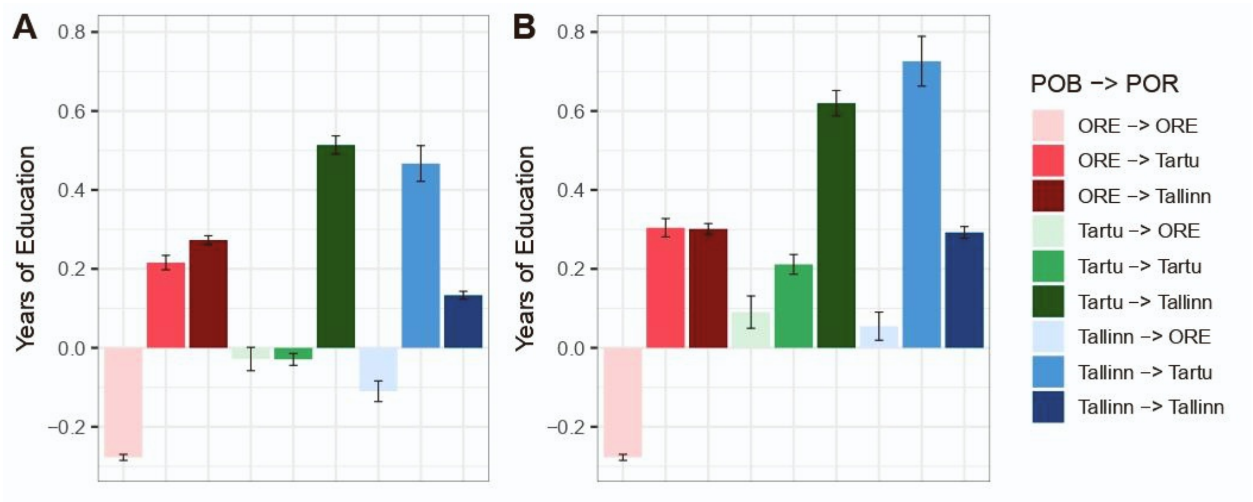

Figure S59. EA (years of education) in migration groups among Estonian participants by region of birth (POB) and residence (POR). (A) County-based analysis where POB and POR refer to Tartu County ("Tartu"), Harju County ("Tallinn") and other counties ("ORE"). (B) City-based analysis, where POB and POR refer to Tartu City ("Tartu"), Tallinn ("Tallinn") and other counties ("ORE"). EA is adjusted for demographic and genetic ancestry covariates. Error bars correspond to 95% confidence intervals.

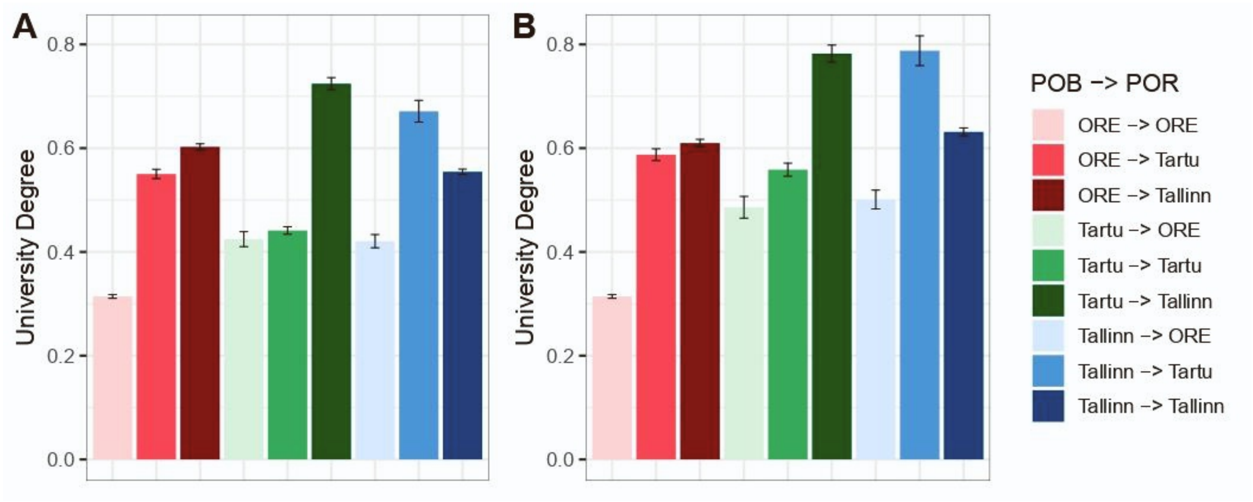

Figure S60. EA (university degree) in migration groups among Estonian participants by region of birth (POB) and residence (POR). (A) County-based analysis where POB and POR refer to Tartu County ("Tartu"), Harju County ("Tallinn") and other counties ("ORE"). (B) City-based analysis, where POB and POR refer to Tartu City ("Tartu"), Tallinn ("Tallinn") and other counties ("ORE"). Error bars correspond to 95% confidence intervals.

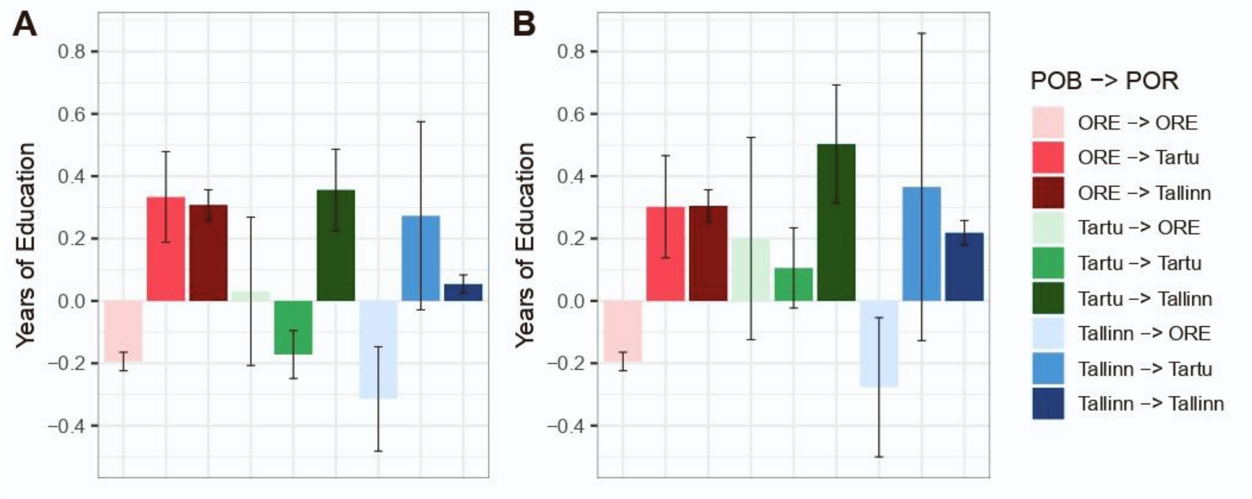

Figure S61. EA (years of education) in migration groups among Russian participants by region of birth (POB) and residence (POR). (A) County-based analysis where POB and POR refer to Tartu County ("Tartu"), Harju County ("Tallinn") and other counties ("ORE"). (B) City-based analysis, where POB and POR refer to Tartu City ("Tartu"), Tallinn ("Tallinn") and other counties ("ORE"). EA is adjusted for demographic and genetic ancestry covariates. Error bars correspond to 95% confidence intervals.

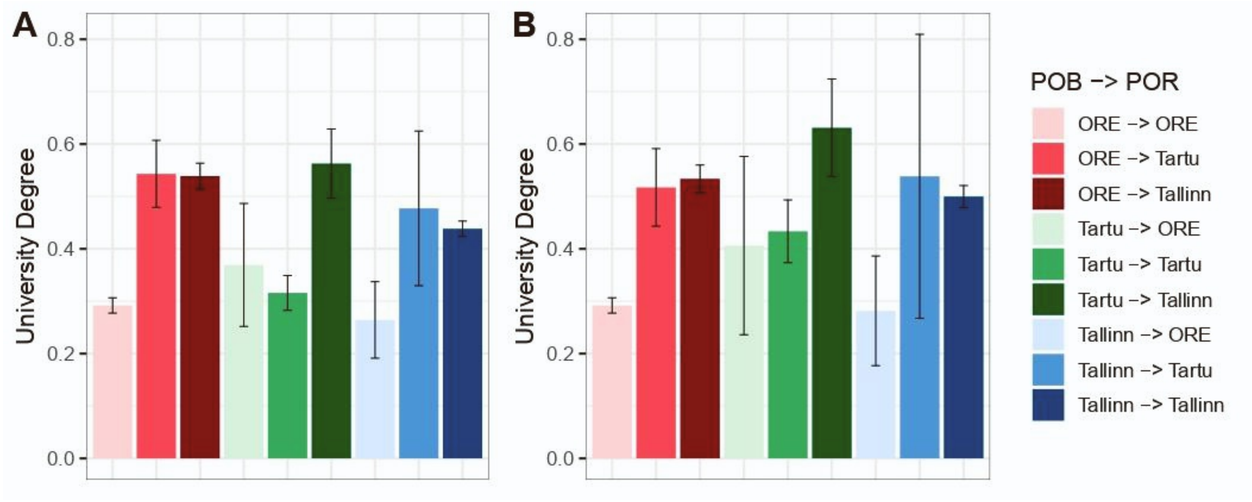

Figure S62. EA (university degree) in migration groups among Russian participants by region of birth (POB) and residence (POR). (A) County-based analysis where POB and POR refer to Tartu County ("Tartu"), Harju County ("Tallinn") and other counties ("ORE"). (B) City-based analysis, where POB and POR refer to Tartu City ("Tartu"), Tallinn ("Tallinn") and other counties ("ORE"). Error bars correspond to 95% confidence intervals.

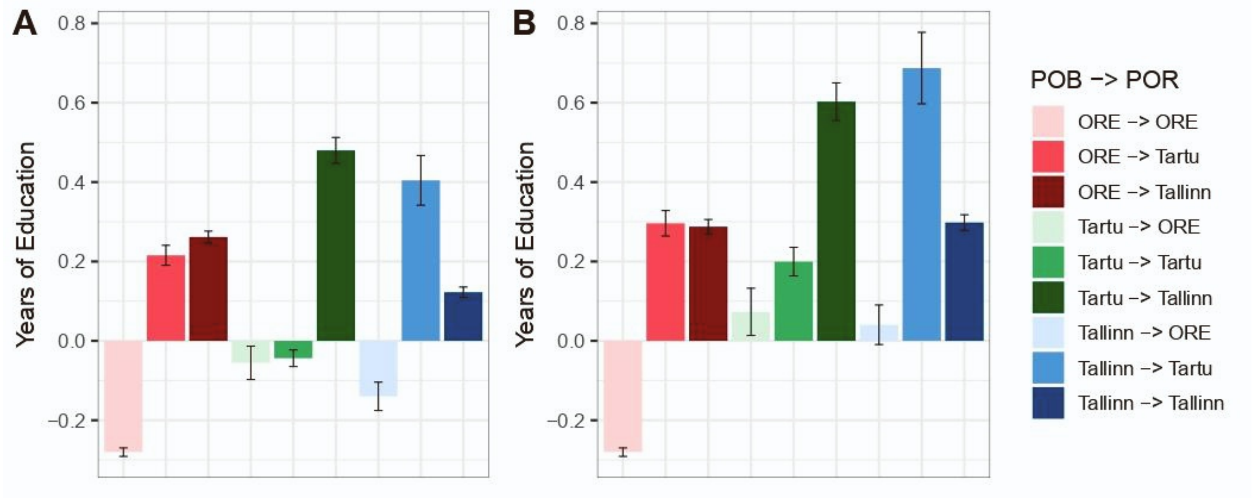

Figure S63. EA (years of education) in migration groups among unrelated Estonian participants by region of birth (POB) and residence (POR). (A) County-based analysis where POB and POR refer to Tartu County ("Tartu"), Harju County ("Tallinn") and other counties ("ORE"). (B) City-based analysis, where POB and POR refer to Tartu City ("Tartu"), Tallinn ("Tallinn") and other counties ("ORE"). EA is adjusted for demographic and genetic ancestry covariates. Error bars correspond to 95% confidence intervals.

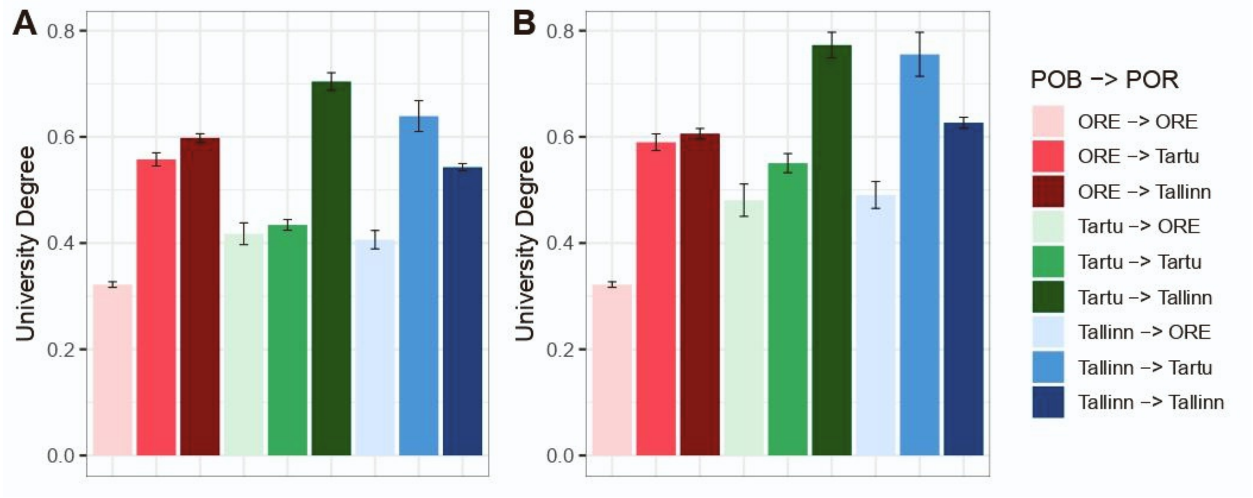

Figure S64. EA (university degree) in migration groups among unrelated Estonian participants by region of birth (POB) and residence (POR). (A) County-based analysis where POB and POR refer to Tartu County ("Tartu"), Harju County ("Tallinn") and other counties ("ORE"). (B) City-based analysis, where POB and POR refer to Tartu City ("Tartu"), Tallinn ("Tallinn") and other counties ("ORE"). Error bars correspond to 95% confidence intervals.

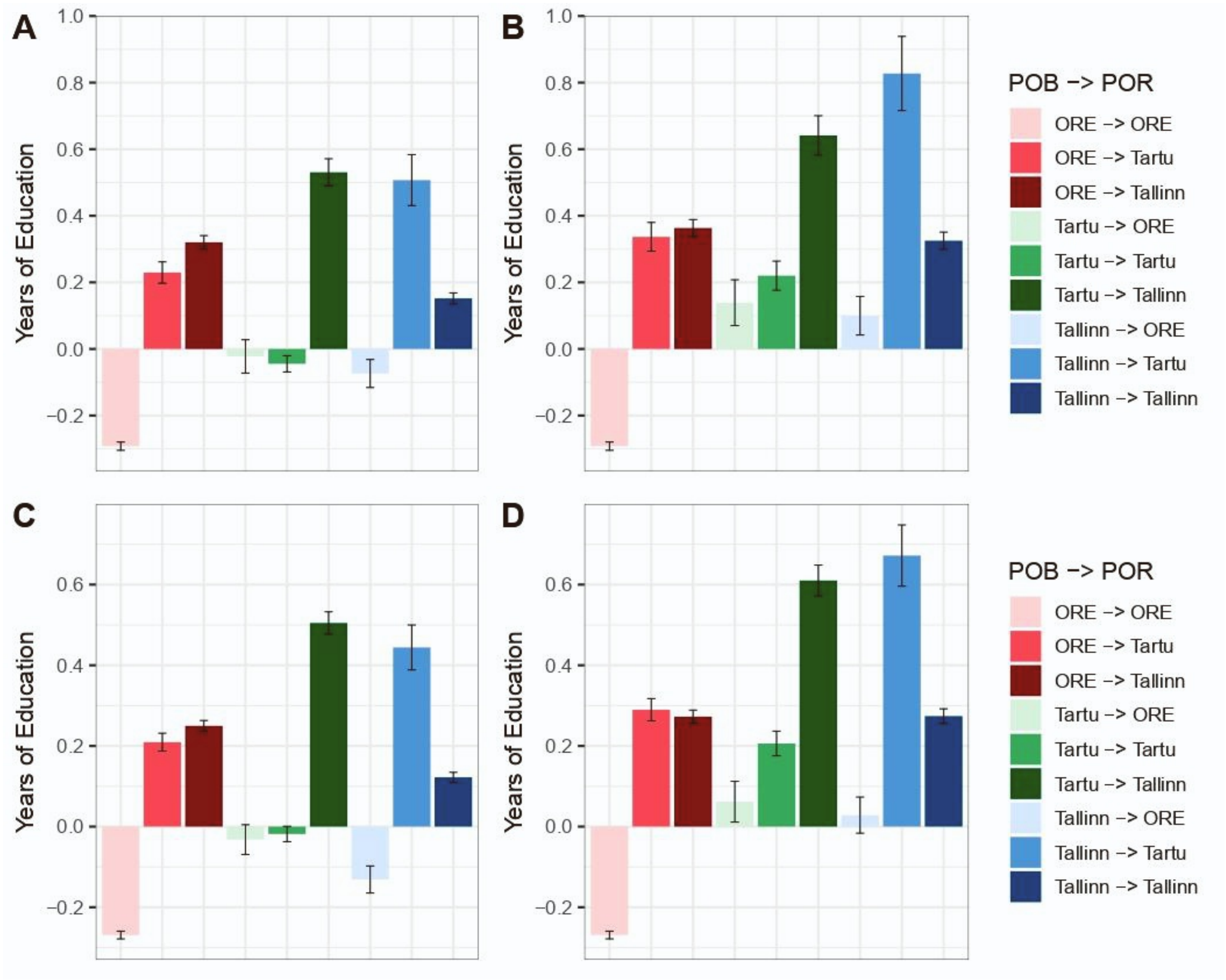

Figure S65. EA (years of education) in migration groups among (A-B) male and (C-D) female Estonian participants by region of birth (POB) and residence (POR). (A, C) County-based analysis where POB and POR refer to Tartu County ("Tartu"), Harju County ("Tallinn") and other counties ("ORE"). (B, D) City-based analysis, where POB and POR refer to Tartu City ("Tartu"), Tallinn ("Tallinn") and other counties ("ORE"). EA is adjusted for demographic and genetic ancestry covariates. Error bars correspond to 95% confidence intervals.

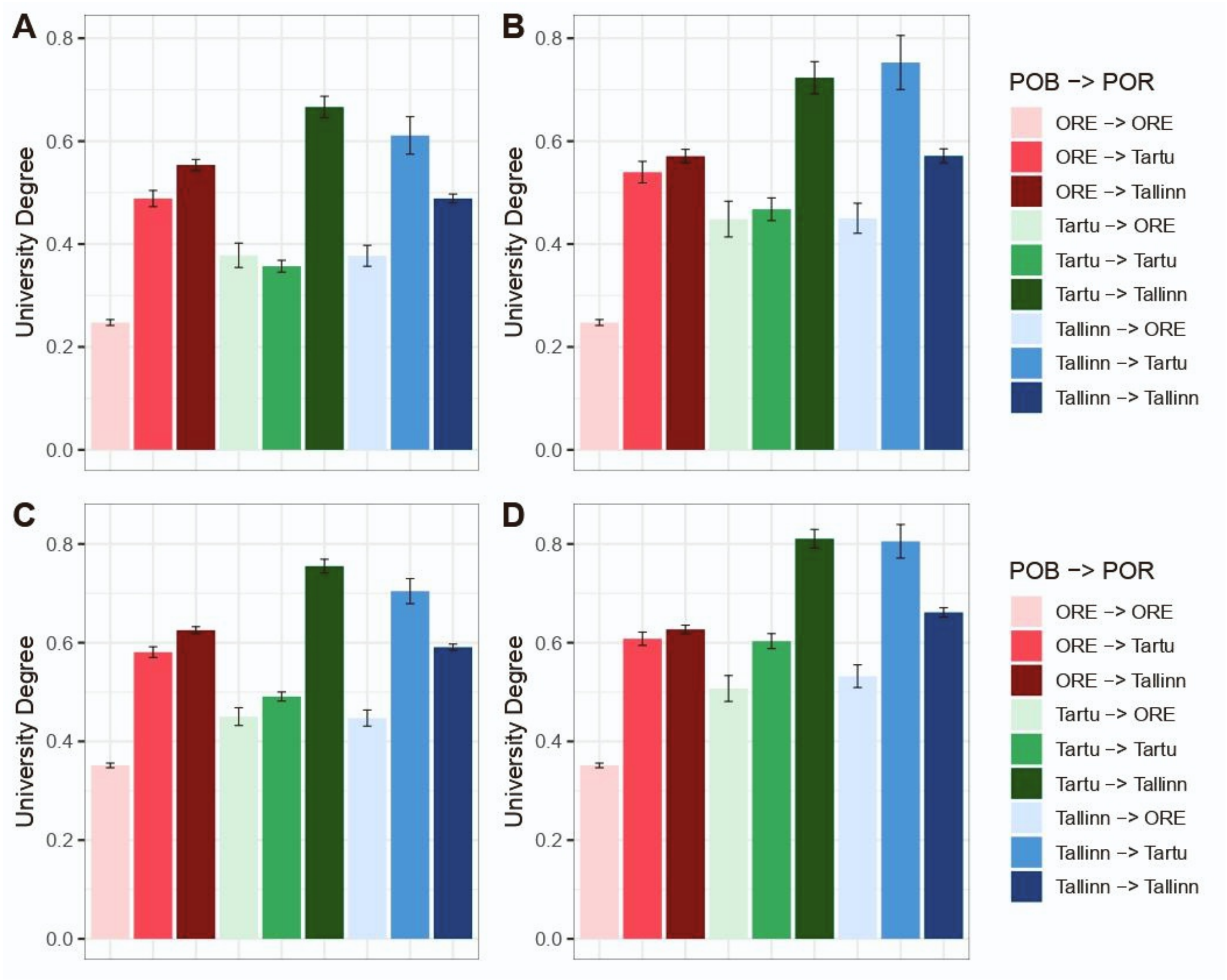

Figure S66. EA (university degree) in migration groups among (A-B) male and (C-D) female Estonian participants by region of birth (POB) and residence (POR).(A, C) County-based analysis where POB and POR refer to Tartu County ("Tartu"), Harju County ("Tallinn") and other counties ("ORE"). (B, D) City-based analysis, where POB and POR refer to Tartu City ("Tartu"), Tallinn ("Tallinn") and other counties ("ORE"). Error bars correspond to 95% confidence intervals.

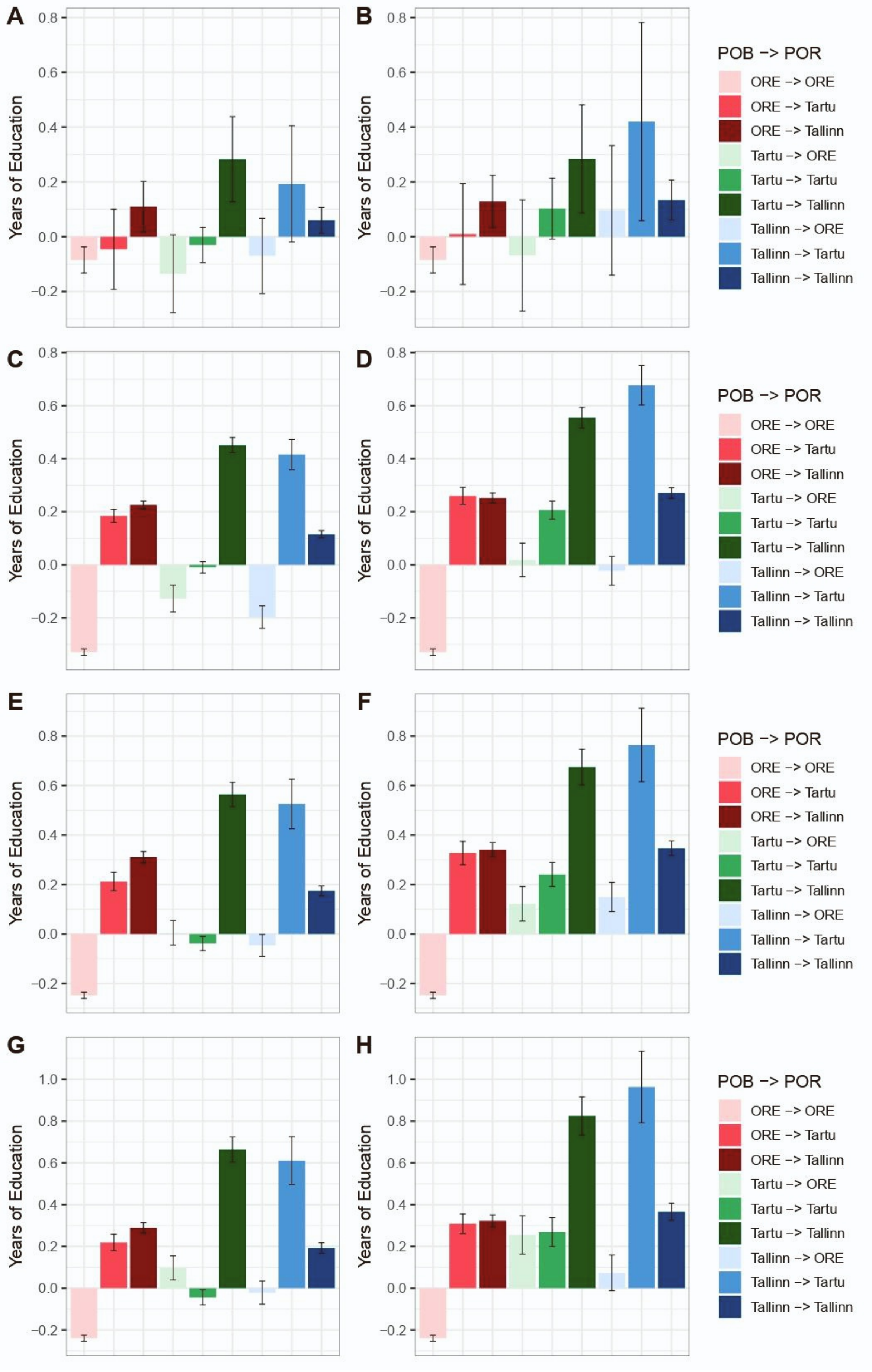
A
B
C
D
E
F
G
H
Years of Education
POB -> POR
ORE -> ORE
ORE -> Tartu
ORE -> Tallinn
Tartu -> ORE
Tartu -> Tartu
Tartu -> Tallinn
Tallinn -> ORE
Tallinn -> Tartu
Tallinn -> Tallinn

Figure S67. EA (years of education) in migration groups among Estonian participants stratified by age by region of birth (POB) and residence (POR). Age groups were defined as (A-B) 18-24, (C-D) 25-48, (E-F) 49-64, (G-H) 65+. (A, C, E, G) County-based analysis where POB and POR refer to Tartu County ("Tartu"), Harju County ("Tallinn") and other counties ("ORE"). (B, D, F, H) City-based analysis, where POB and POR refer to Tartu City ("Tartu"), Tallinn ("Tallinn") and other counties ("ORE"). EA is adjusted for demographic and genetic ancestry covariates. Error bars correspond to 95% confidence intervals.

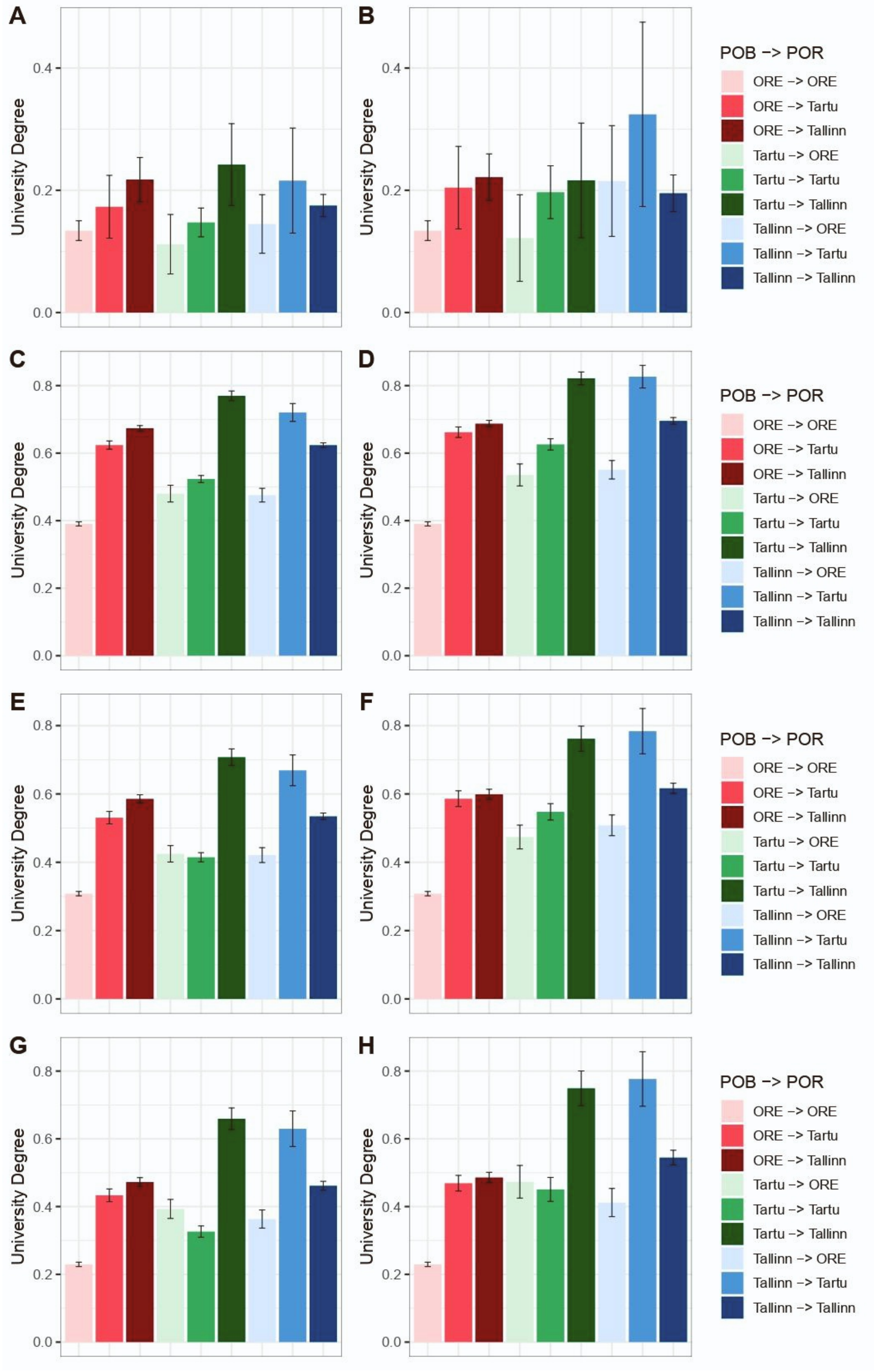
A
B
C
D
E
F
G
H
University Degree
POB -> POR
ORE -> ORE
ORE -> Tartu
ORE -> Tallinn
Tartu -> ORE
Tartu -> Tartu
Tartu -> Tallinn
Tallinn -> ORE
Tallinn -> Tartu
Tallinn -> Tallinn

Figure S68. EA (university degree) in migration groups among Estonian participants stratified by age by region of birth (POB) and residence (POR). Age groups were defined as (A-B) 18-24, (C-D) 25-48, (E-F) 49-64, (G-H) 65+. (A, C, E, G) County-based analysis where POB and POR refer to Tartu County ("Tartu"), Harju County ("Tallinn") and other counties ("ORE"). (B, D, F, H) City-based analysis, where POB and POR refer to Tartu City ("Tartu"), Tallinn ("Tallinn") and other counties ("ORE"). Error bars correspond to 95% confidence intervals.

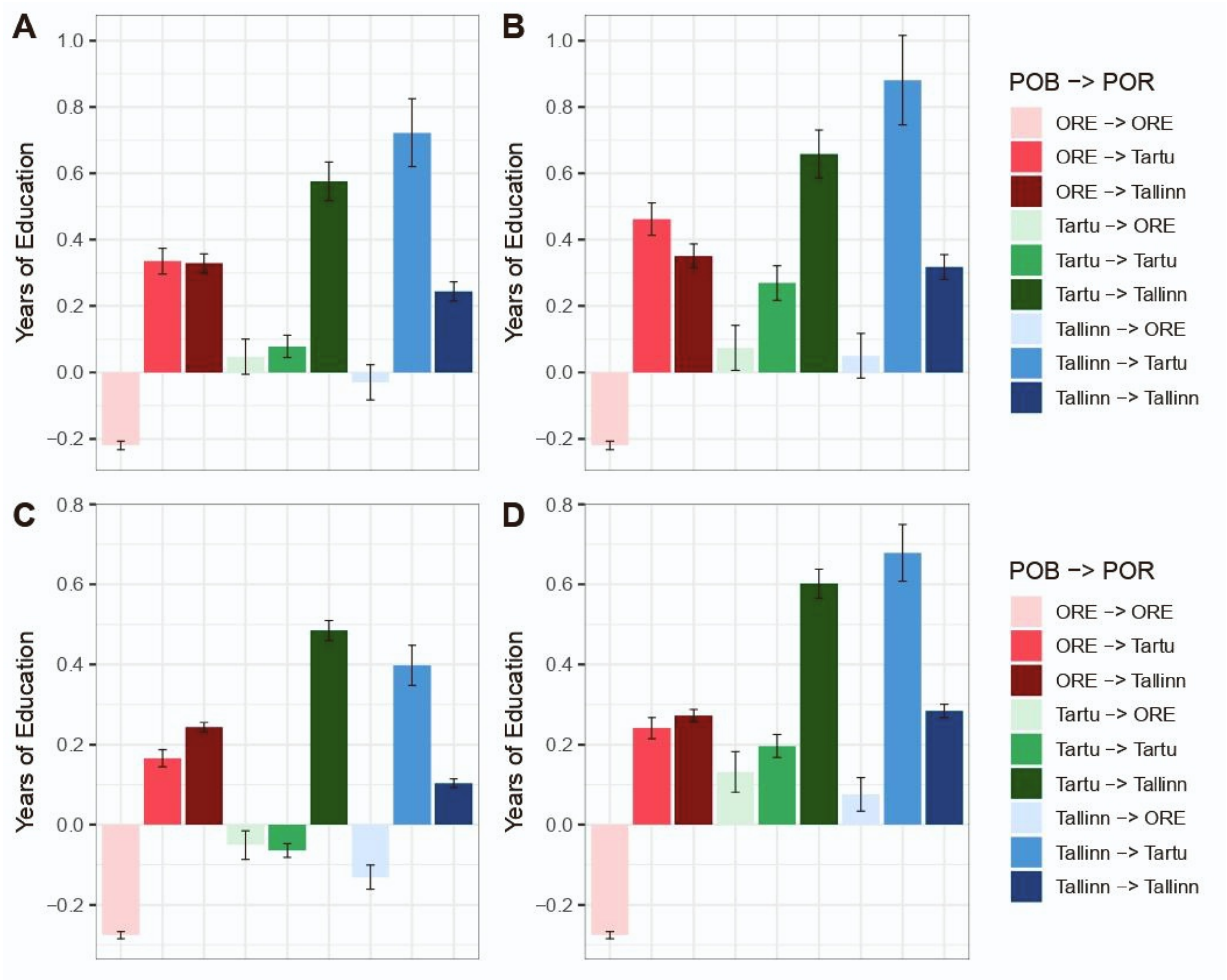

Figure S69. EA (years of education) in migration groups among Estonian participants stratified by year of joining the biobank by region of birth (POB) and residence (POR). The periods of joining are (A-B) 2001-2016 and (C-D) 2017-2021. (A, C) County-based analysis where POB and POR refer to Tartu County ("Tartu"), Harju County ("Tallinn") and other counties ("ORE"). (B, D) City-based analysis, where POB and POR refer to Tartu City ("Tartu"), Tallinn ("Tallinn") and other counties ("ORE"). EA is adjusted for demographic and genetic ancestry covariates. Error bars correspond to 95% confidence intervals.

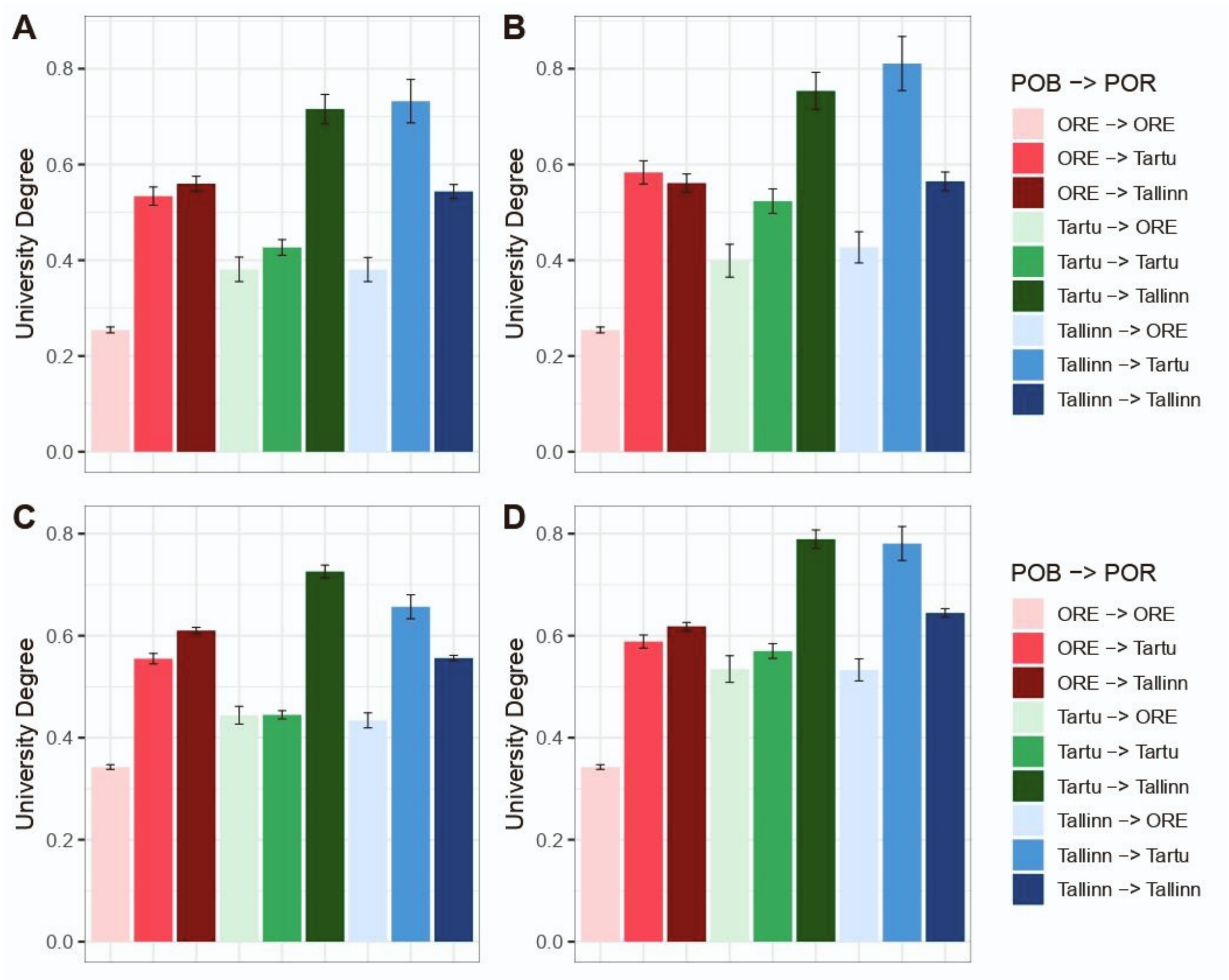

Figure S70. EA (university degree) in migration groups among Estonian participants stratified by year of joining the biobank by region of birth (POB) and residence (POR). The periods of joining are (A-B) 2001-2016 and (C-D) 2017-2021. (A, C) County-based analysis where POB and POR refer to Tartu County ("Tartu"), Harju County ("Tallinn") and other counties ("ORE"). (B, D) City-based analysis, where POB and POR refer to Tartu City ("Tartu"), Tallinn ("Tallinn") and other counties ("ORE"). Error bars correspond to 95% confidence intervals.

(s)PGS_{EA} with EA regressed out in groups with different migration profiles

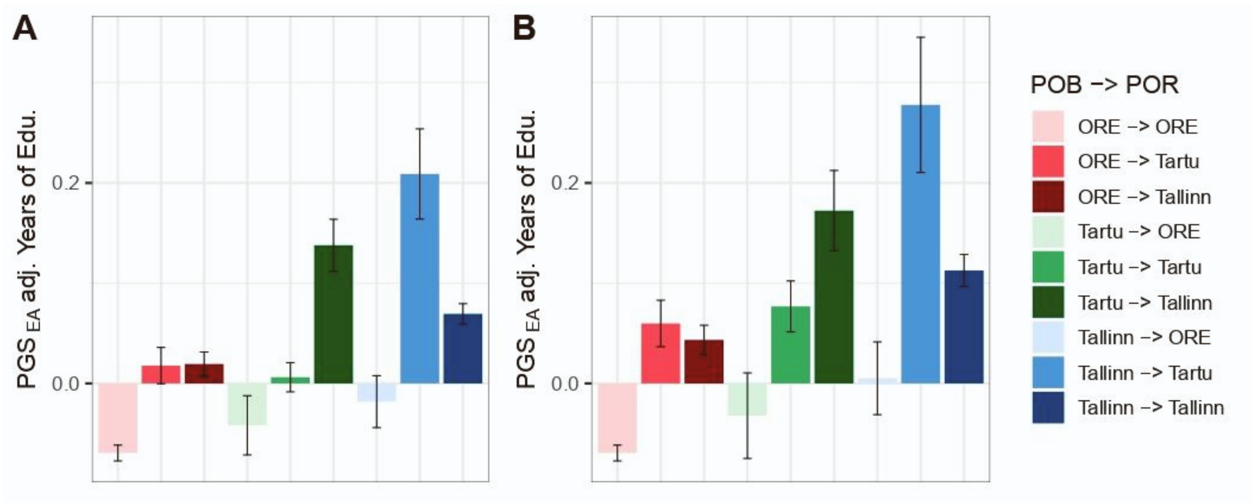

Figure S71. PGS_{EA} with EA (years of education) regressed out in migration groups among Estonian participants by region of birth (POB) and residence (POR). (A) County-based analysis where POB and POR refer to Tartu County ("Tartu"), Harju County ("Tallinn") and other counties ("ORE"). (B) City-based analysis, where POB and POR refer to Tartu City ("Tartu"), Tallinn ("Tallinn") and other counties ("ORE"). PGS_{EA} is adjusted also for demographic and genetic ancestry covariates. Error bars correspond to 95% confidence intervals.

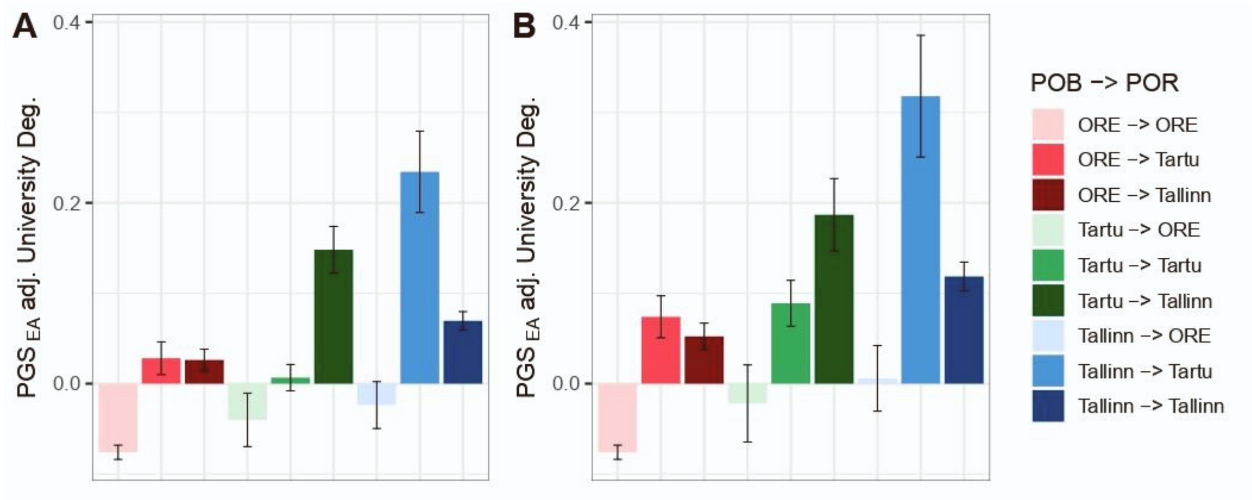

Figure S72. PGS_{EA} with EA (university degree) regressed out in migration groups among Estonian participants by region of birth (POB) and residence (POR). (A) County-based analysis where POB and POR refer to Tartu County ("Tartu"), Harju County ("Tallinn") and other counties ("ORE"). (B) City-based analysis, where POB and POR refer to Tartu City ("Tartu"), Tallinn ("Tallinn") and other counties ("ORE"). PGS_{EA} is adjusted also for demographic and genetic ancestry covariates. Error bars correspond to 95% confidence intervals.

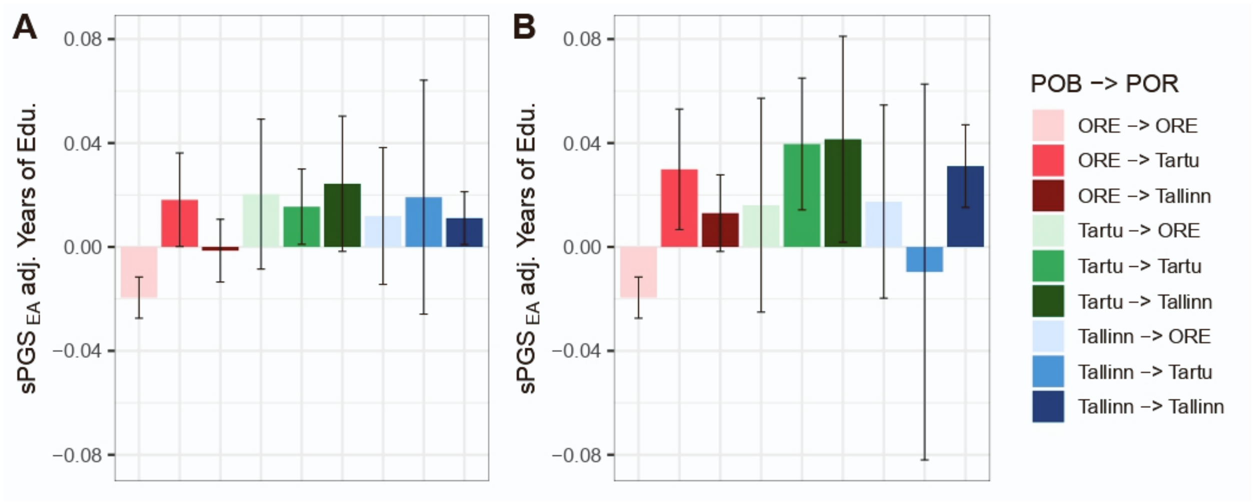

Figure S73. sPGS$_{EA}$ with EA (years of education) regressed out in migration groups among Estonian participants by region of birth (POB) and residence (POR). (A) County-based analysis where POB and POR refer to Tartu County (“Tartu”), Harju County (“Tallinn”) and other counties (“ORE”). (B) City-based analysis, where POB and POR refer to Tartu City (“Tartu”), Tallinn (“Tallinn”) and other counties (“ORE”). sPGS$_{EA}$ is adjusted also for demographic and genetic ancestry covariates. Error bars correspond to 95% confidence intervals.

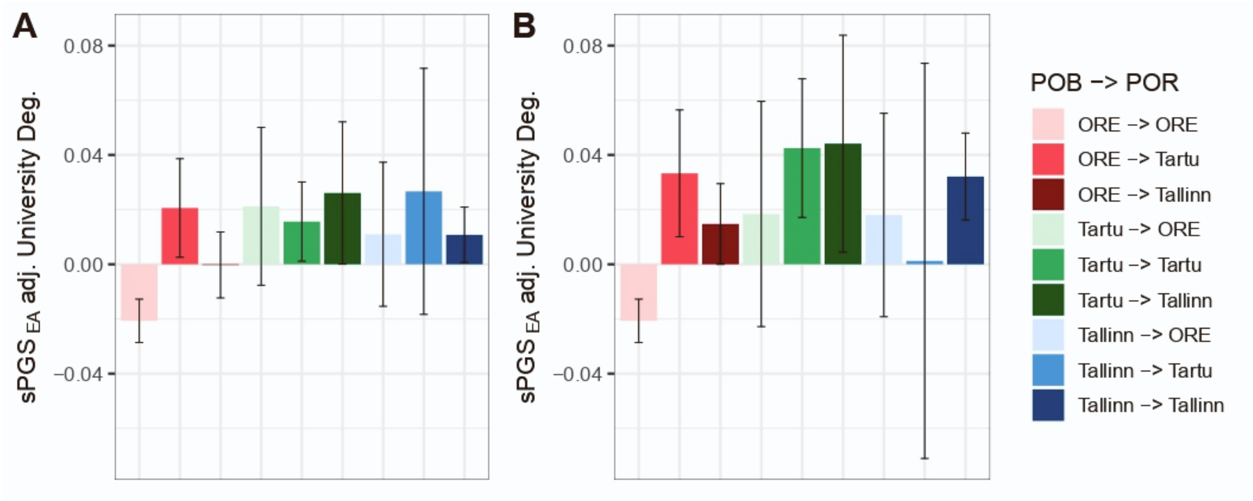

Figure S74. sPGS$_{EA}$ with EA (university degree) regressed out in migration groups among Estonian participants by region of birth (POB) and residence (POR). (A) County-based analysis where POB and POR refer to Tartu County (“Tartu”), Harju County (“Tallinn”) and other counties (“ORE”). (B) City-based analysis, where POB and POR refer to Tartu City (“Tartu”), Tallinn (“Tallinn”) and other counties (“ORE”). sPGS$_{EA}$ is adjusted also for demographic and genetic ancestry covariates. Error bars correspond to 95% confidence intervals.

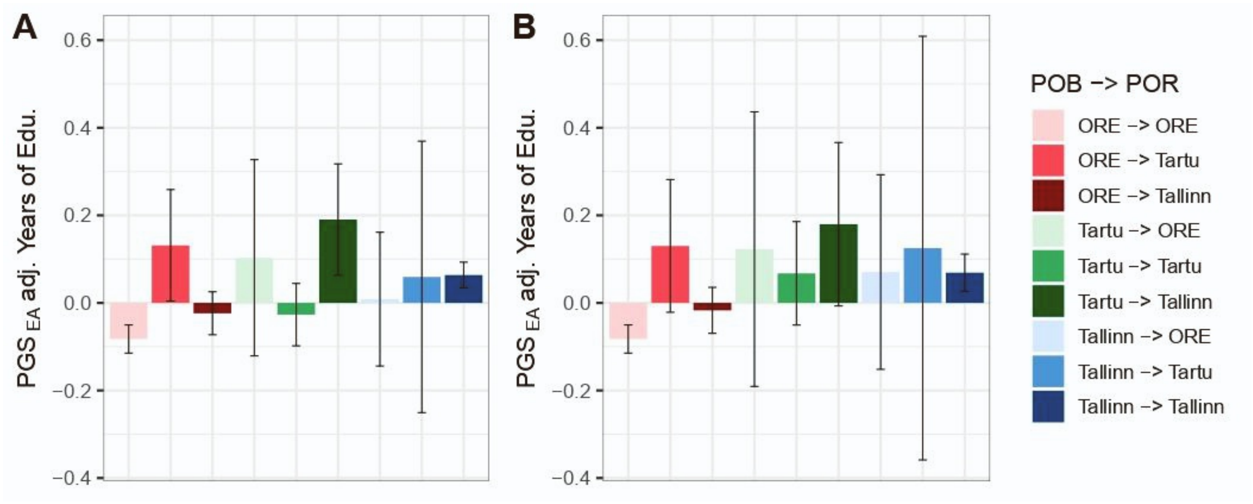

Figure S75. PGS_{EA} with EA (years of education) regressed out in migration groups among Russian participants by region of birth (POB) and residence (POR). (A) County-based analysis where POB and POR refer to Tartu County ("Tartu"), Harju County ("Tallinn") and other counties ("ORE"). (B) City-based analysis, where POB and POR refer to Tartu City ("Tartu"), Tallinn ("Tallinn") and other counties ("ORE"). PGS_{EA} is adjusted also for demographic and genetic ancestry covariates. Error bars correspond to 95% confidence intervals.

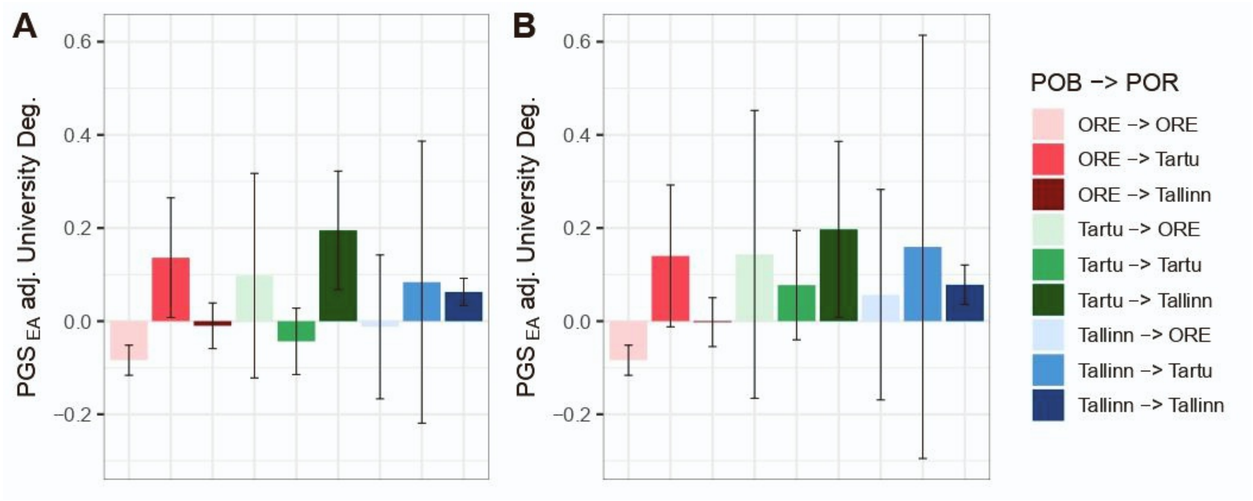

Figure S76. PGS_{EA} with EA (university degree) regressed out in migration groups among Russian participants by region of birth (POB) and residence (POR). (A) County-based analysis where POB and POR refer to Tartu County ("Tartu"), Harju County ("Tallinn") and other counties ("ORE"). (B) City-based analysis, where POB and POR refer to Tartu City ("Tartu"), Tallinn ("Tallinn") and other counties ("ORE"). PGS_{EA} is adjusted also for demographic and genetic ancestry covariates. Error bars correspond to 95% confidence intervals.

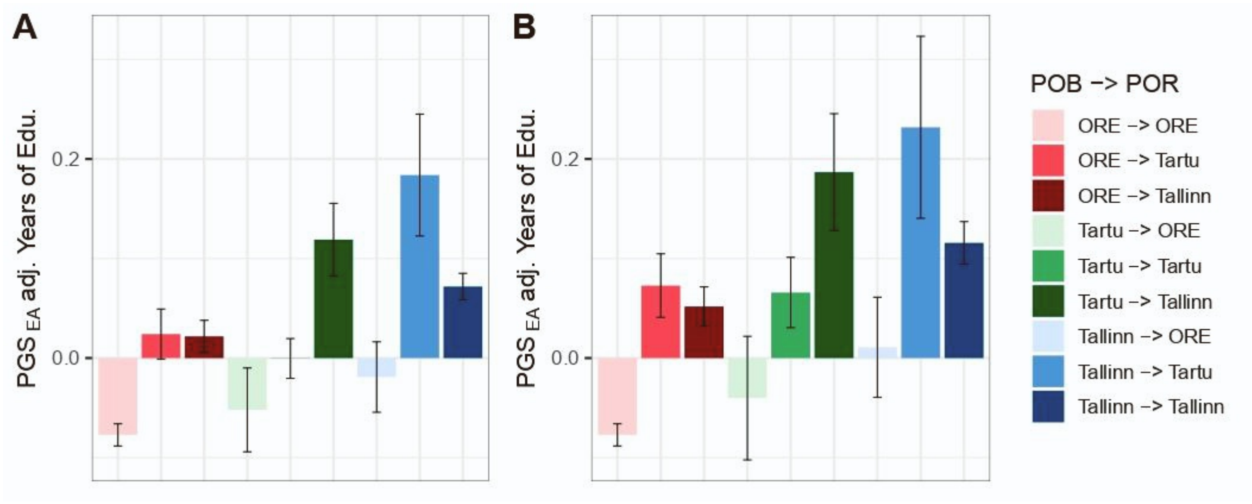

Figure S77. PGS_{EA} with EA (years of education) regressed out in migration groups among unrelated Estonian participants by region of birth (POB) and residence (POR). (A) County-based analysis where POB and POR refer to Tartu County ("Tartu"), Harju County ("Tallinn") and other counties ("ORE"). (B) City-based analysis, where POB and POR refer to Tartu City ("Tartu"), Tallinn ("Tallinn") and other counties ("ORE"). PGS_{EA} is adjusted also for demographic and genetic ancestry covariates. Error bars correspond to 95% confidence intervals.

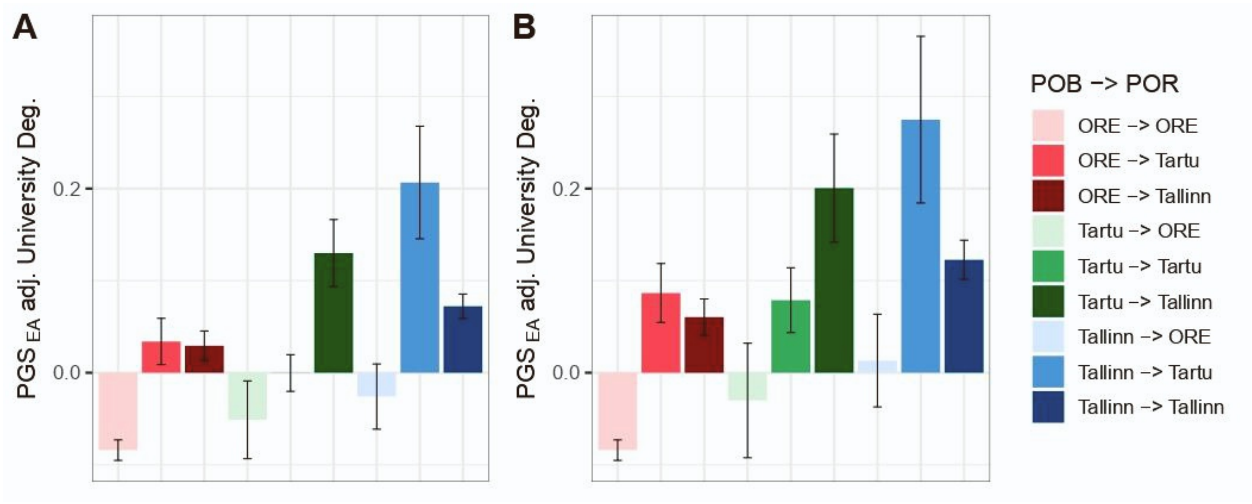

Figure S78. PGS_{EA} with EA (university degree) regressed out in migration groups among unrelated Estonian participants by region of birth (POB) and residence (POR). (A) County-based analysis where POB and POR refer to Tartu County ("Tartu"), Harju County ("Tallinn") and other counties ("ORE"). (B) City-based analysis, where POB and POR refer to Tartu City ("Tartu"), Tallinn ("Tallinn") and other counties ("ORE"). PGS_{EA} is adjusted also for demographic and genetic ancestry covariates. Error bars correspond to 95% confidence intervals.

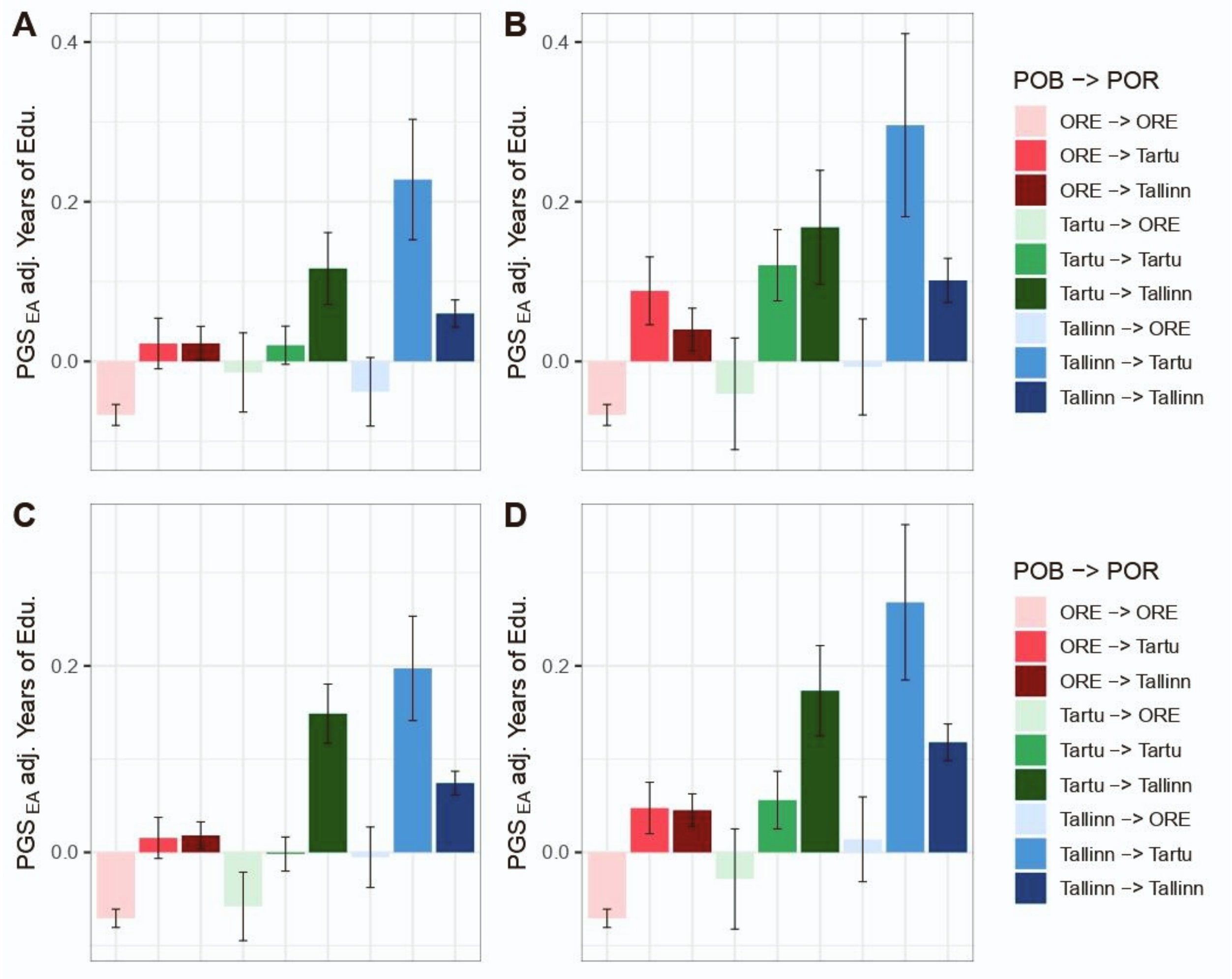

Figure S79. PGS_{EA} with EA (years of education) regressed out in migration groups among (A-B) male and (C-D) female Estonian participants by region of birth (POB) and residence (POR). (A, C) County-based analysis where POB and POR refer to Tartu County ("Tartu"), Harju County ("Tallinn") and other counties ("ORE"). (B, D) City-based analysis, where POB and POR refer to Tartu City ("Tartu"), Tallinn ("Tallinn") and other counties ("ORE"). PGS_{EA} is adjusted also for demographic and genetic ancestry covariates. Error bars correspond to 95% confidence intervals.

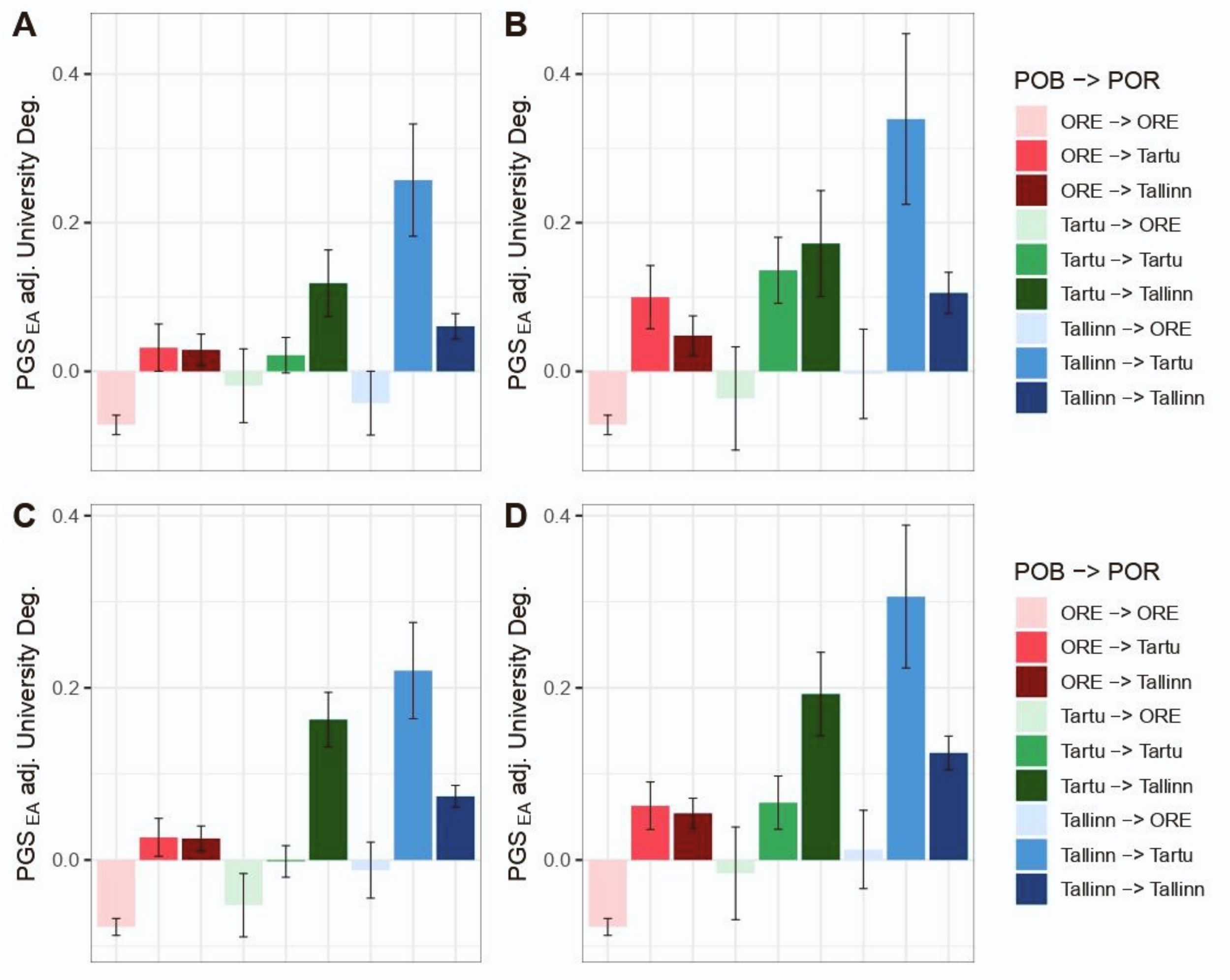

Figure S80. PGS$_{EA}$ with EA (university degree) regressed out in migration groups among (A-B) male and (C-D) female Estonian participants by region of birth (POB) and residence (POR). (A, C) County-based analysis where POB and POR refer to Tartu County ("Tartu"), Harju County ("Tallinn") and other counties ("ORE"). (B, D) City-based analysis, where POB and POR refer to Tartu City ("Tartu"), Tallinn ("Tallinn") and other counties ("ORE"). PGS$_{EA}$ is adjusted also for demographic and genetic ancestry covariates. Error bars correspond to 95% confidence intervals.

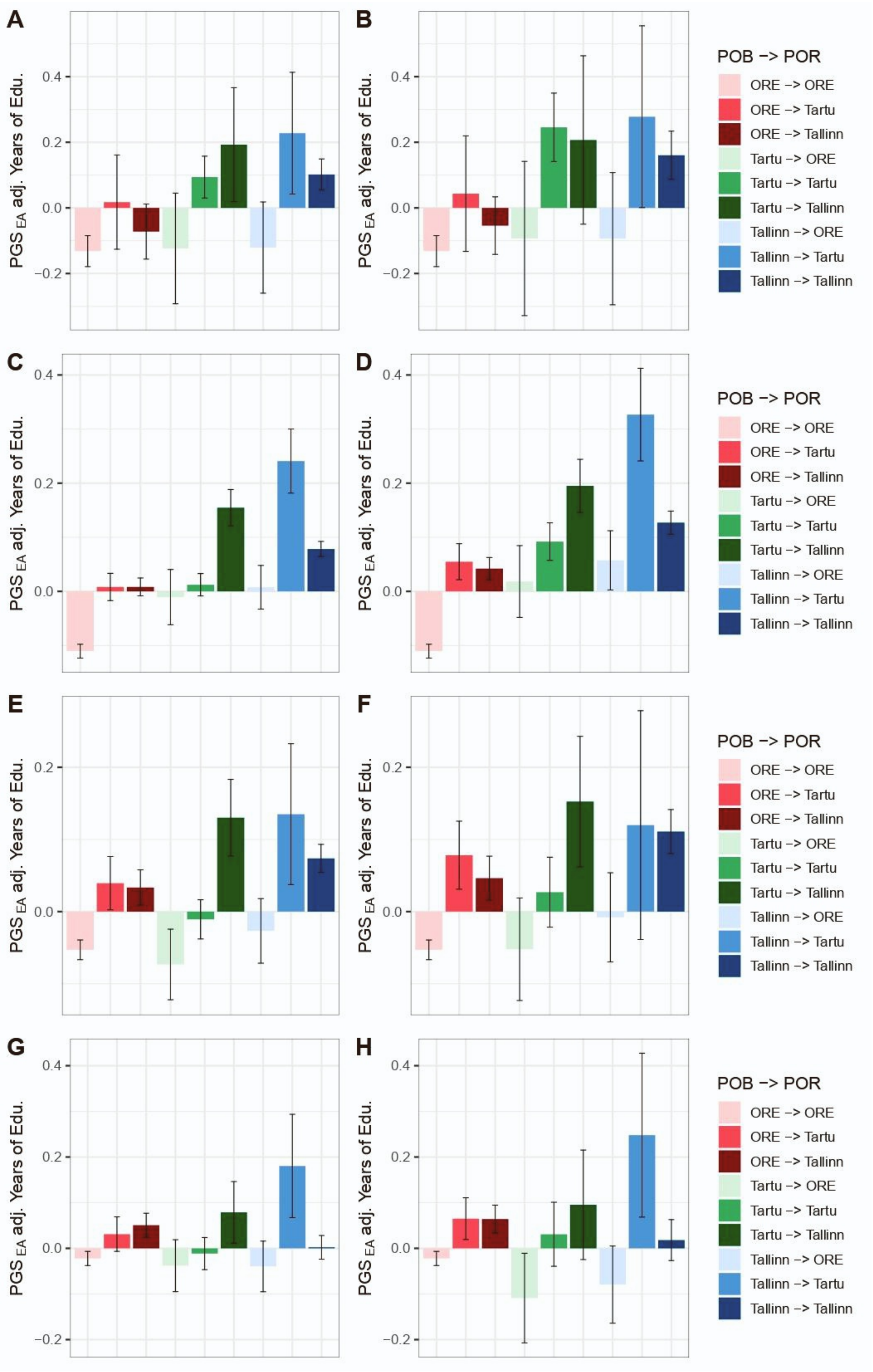
A
B
C
D
E
F
G
H
PGS EA adj. Years of Edu.
0.4
0.2
0.0
−0.2
POB −> POR
ORE −> ORE
ORE −> Tartu
ORE −> Tallinn
Tartu −> ORE
Tartu −> Tartu
Tartu −> Tallinn
Tallinn −> ORE
Tallinn −> Tartu
Tallinn −> Tallinn

Figure S81. PGS_{EA} with EA (years of education) regressed out in migration groups among Estonian participants stratified by age by region of birth (POB) and residence (POR). Age groups were defined as (A-B) 18-24, (C-D) 25-48, (E-F) 49-64, (G-H) 65+. (A, C, E, G) County-based analysis where POB and POR refer to Tartu County ("Tartu"), Harju County ("Tallinn") and other counties ("ORE"). (B, D, F, H) City-based analysis, where POB and POR refer to Tartu City ("Tartu"), Tallinn ("Tallinn") and other counties ("ORE"). PGS_{EA} is adjusted also for demographic and genetic ancestry covariates. Error bars correspond to 95% confidence intervals.

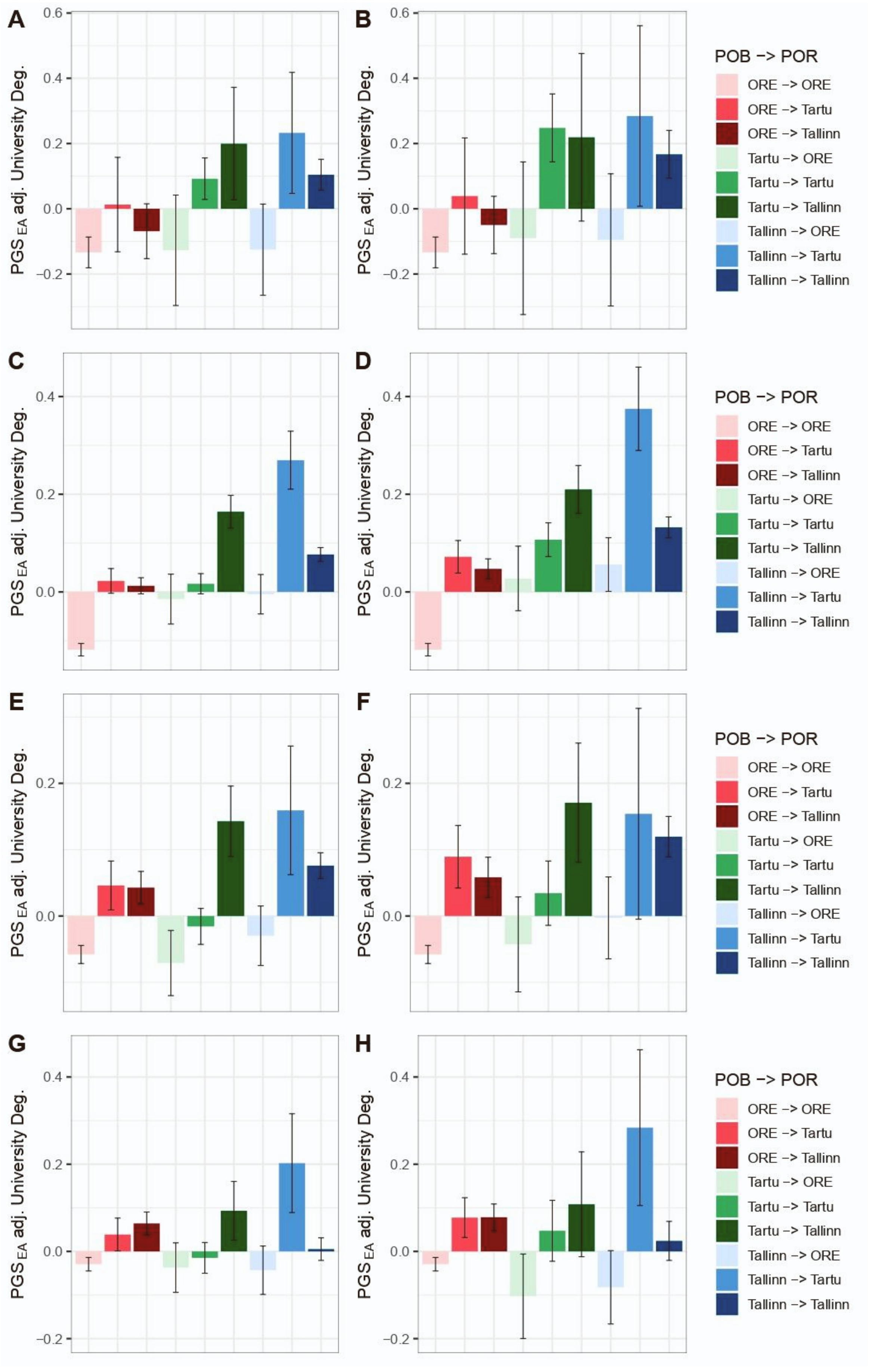
A
B
C
D
E
F
G
H
PGS EA adj. University Deg.
POB -> POR
ORE -> ORE
ORE -> Tartu
ORE -> Tallinn
Tartu -> ORE
Tartu -> Tartu
Tartu -> Tallinn
Tallinn -> ORE
Tallinn -> Tartu
Tallinn -> Tallinn

Figure S82. PGS$_{EA}$ with EA (university degree) regressed out in migration groups among Estonian participants stratified by age by region of birth (POB) and residence (POR). Age groups were defined as (A-B) 18-24, (C-D) 25-48, (E-F) 49-64, (G-H) 65+. (A, C, E, G) County-based analysis where POB and POR refer to Tartu County ("Tartu"), Harju County ("Tallinn") and other counties ("ORE"). (B, D, F, H) City-based analysis, where POB and POR refer to Tartu City ("Tartu"), Tallinn ("Tallinn") and other counties ("ORE"). PGS$_{EA}$ is adjusted also for demographic and genetic ancestry covariates. Error bars correspond to 95% confidence intervals.

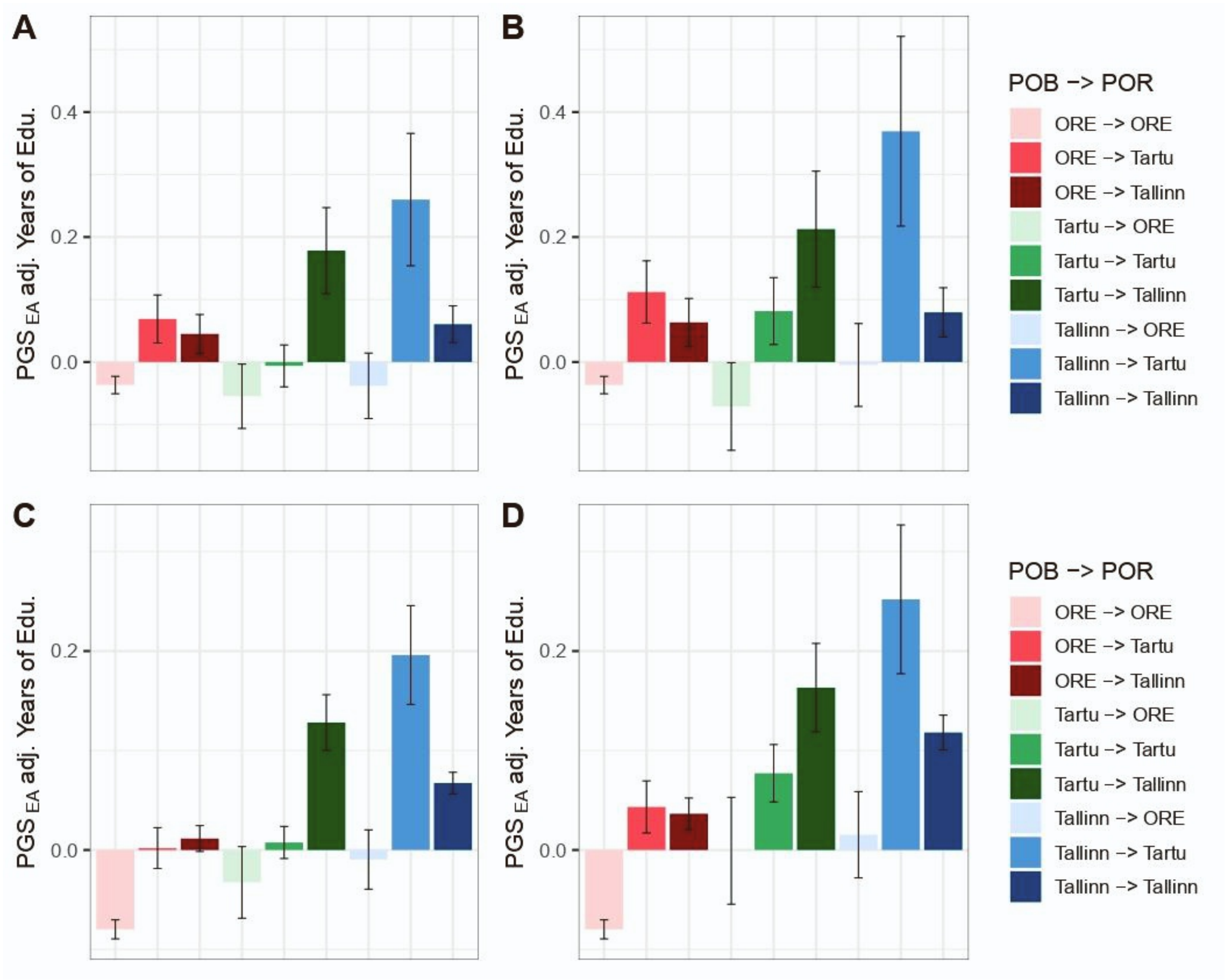

Figure S83. PGS_{EA} with EA (university degree) regressed out in migration groups among Estonian participants stratified by year of joining the biobank by region of birth (POB) and residence (POR). The periods of joining are (A-B) 2001-2016 and (C-D) 2017-2021. (A, C) County-based analysis where POB and POR refer to Tartu County ("Tartu"), Harju County ("Tallinn") and other counties ("ORE"). (B, D) City-based analysis, where POB and POR refer to Tartu City ("Tartu"), Tallinn ("Tallinn") and other counties ("ORE"). PGS_{EA} is adjusted also for demographic and genetic ancestry covariates. Error bars correspond to 95% confidence intervals.

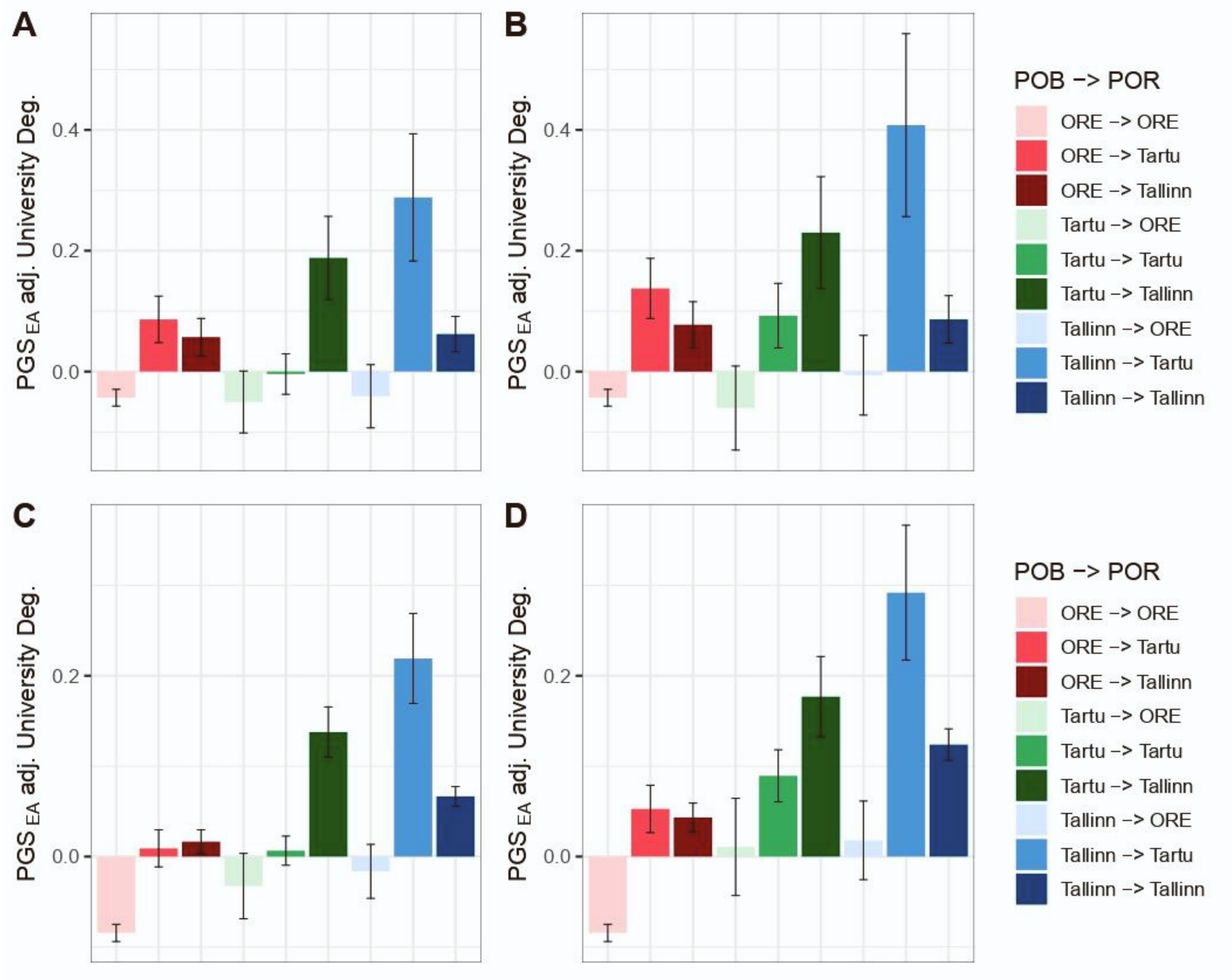

Figure S84. PGS$_{EA}$ with EA (years of education) regressed out in migration groups among Estonian participants stratified by year of joining the biobank by region of birth (POB) and residence (POR). The periods of joining are (A-B) 2001-2016 and (C-D) 2017-2021. (A, C) County-based analysis where POB and POR refer to Tartu County ("Tartu"), Harju County ("Tallinn") and other counties ("ORE"). (B, D) City-based analysis, where POB and POR refer to Tartu City ("Tartu"), Tallinn ("Tallinn") and other counties ("ORE"). PGS$_{EA}$ is adjusted also for demographic and genetic ancestry covariates. Error bars correspond to 95% confidence intervals.

Supplemental Tables

Table S3. Mapping from Education level to Years of education and University degree.

Education level	ISCED 2011 mapping	Years of education	University degree
Early childhood education	0	1	0
Primary education	1	7	0
Lower secondary education	2	10	0
Upper secondary education	2	10	0
Post-secondary non-tertiary education	3	13	0
Short-cycle tertiary education	4	15	0
Bachelor's or equivalent level	6	18	1
Master's or equivalent level	7	20	1
Doctoral or equivalent level	8	22	1
