# Supplementary material for: Genetic effects on migration behavior contribute to increasing spatial differentiation at trait-associated loci in Estonia
Source: iScience. 2025 Nov 12;28(12):114013. doi: 10.1016/j.isci.2025.114013 (PMC12702256; doi:10.1016/j.isci.2025.114013)
Supplement: Table S11. Fraction of the inter-individual variance of psychiatric trait PGSs explained by county of birth (POB) and county of residence (POR) [file mmc12.pdf]

Content

Supplemental Note 1. Estonian Biobank cohort overview **2**
Supplemental Note 2. Intensity of the internal migration in Estonia **4**
Supplemental Note 3. Polygenic scores based on within-sibship GWAS summary statistics (sPGS) **5**
Supplemental Note 4. Robustness of Var_{county} estimates **6**
Analysis of principal components and polygenic scores in subgroups 6
Cumulative adjustment of PGS_{EA} for the top 100 PCs 7
Adjustment of PGS_{EA} for the complete genetic relatedness matrix (GRM) in a leave one chromosome out (LOCO) approach 7
Supplemental Note 5. Replication of the main results in unrelated Estonian individuals **9**
Supplemental Note 6. Replication of the main results with PGS_{EA4} **10**
Supplemental Note 7. Analysis of psychiatric trait PGSs **11**
Supplemental Note 8. Selective migration and correlations between mate-pair PGSs **12**
Supplemental Note 9. How large are the regional differences in PGS_{EA}? **14**
Supplemental References **15**

Supplemental Note 1. Estonian Biobank cohort overview

The Estonian Biobank (EstBB) cohort is a volunteer-based sample of the Estonian resident adult population (aged ≥18 years)[1,2]. EstBB participants are genotyped and deeply phenotyped. The phenotype information includes medical records, as well as self-reported medical, lifestyle, demographic, socio-economic, and geographical data. The current number of participants exceeds 210,000 which corresponds to approximately 20% of the contemporary adult population of Estonia. Compared to the UK Biobank[3] it has a more even representation of different age groups and different regions of the country. All of this makes EstBB a good dataset for independent replication of previous findings and novel analyses.

The EstBB Project was initiated in 1999. Since then two main waves of recruitment were conducted (Figure S1A). Approximately 52,000 individuals were recruited until 2016[1] and three times more after 2016[2]. These two periods substantially differed in the recruitment strategy. During the first stage, the participating proposals were mainly spread by general practitioners to their patients. The second stage of recruitment was conducted through a wide-scale advertising campaign.

The data in the biobank is being regularly updated. Information on the level of education and the place of residence is synchronised with the population register database, therefore we used current age or age of death in this study which is almost equal to using a year of birth for separate-generation analysis. The proportion of dead participants is 2.3%. For only 0.2% of the participants, the age group ("18-24", "25-48", "49-64", "65 and older") mismatches with the corresponding birth year group ("1998-2004", "1974-1997", "1958-1973", "1957 and earlier"), calculated as the difference between 2022 and corresponding boundaries of the age bins. Thus, the effects of such mismatches are expected to be negligible. There are no age restrictions in the recruitment process besides being 18 or older. As a consequence, a full range of ages are covered by the cohort with the oldest participant being 105 (Figure S1B).

There are two major ethnic groups (defined based on self-reported ethnicity) currently living in Estonia: Etonians (920,000; 69% of all the people residing in Estonia) and Russians (315,000; 24%), based on the 2021 Population census[4]. The EstBB cohort covers both groups, although Russians are underrepresented in comparison to Estonians. However, the administrative regions are covered relatively uniformly by participants from these groups with 13-31% of Estonians sampled and 1-7% of Russians (Figure S2) from each county. Here we use the data only on the participants of self-reported Estonian or Russian ethnicity, excluding individuals who indicated both. Throughout the study, we focused mainly on the Estonian group because of the statistical power available and replicated some analyses in the Russian group.

For the sake of the robustness of the findings as well as for analysis of differences between subgroups, we divided the overall sample into two cohorts based on self-reported ethnicity: Estonians and Russians. The Estonian cohort was further divided into several partially overlapping groups: unrelated individuals (excluding one individual from each pair of closer than 2nd-degree relationship), males, females, Estonians of age 18 - 24, 25 - 48, 48 - 64, 65 and older, and two groups by the period of recruitment (agreement): 2001-2016 and 2017-2021 (Figure S3).

We repeated most of our analyses in the above-mentioned subgroups of the Estonian cohort and in the Russian cohort as well as on the entire Estonian cohort but using polygenic scores based on summary statistics from a within-sibship GWAS (sPGS, Supplemental Note 3)[5]. We also conducted analyses of the distribution of EA phenotypes across regions and migration directions for subgroups within the Estonian cohort, as well as for the Russian cohort. The order of these results coincides with the structure of the main text. Detailed descriptions of the analyses can be found in the Results and Methods section of the main text. For each type of analysis where all the subsample categories are presented, we followed the order: "entire Estonian cohort", "Russian participants", "unrelated Estonian participants", "Estonian subgroups by sex", "Estonian subgroups by age", "Estonian subgroups by year of joining the biobank". Where applicable, results of the same analysis with $sPGS_{EA}$ are presented.

Supplemental Note 2. Intensity of the internal migration in Estonia

The migration flows between the counties of Estonia are not in equilibrium (Figure S4). The highest net migration is to Tallinn, the capital and largest city of Estonia. The second highest net migration is to Tartu, the major city in Southern Estonia and the largest educational centre in the country.

To access migration flows between counties we utilised the information from both the 2021 Census and the EstBB. Mainly, we used the EstBB as a source of information on the migration flows between the counties. However, the sampling density is not uniform across the country, with Tartu County being the most overrepresented region (Figure S2). Without adjustment for the differences in the sampling density, we expect an overestimation of such migration directions as Tartu County. To correct for this effect, we introduced county-specific weights for the study participants:

$$w_i = \frac{n_i}{N} \cdot \frac{N^{EBB}}{n_i^{EBB}},$$

Where w_i is the weight for a study participant residing in a county i, n_i is the number of individuals residing in the county i according to the 2021 Census, N is the number of individuals residing in Estonia according to the 2021 Census, n_i^{EBB} is the number of the study participants residing in the county i, N^{EBB} is the overall number of the study participants. We calculated these weights separately for self-reported Estonian and Russian adults. The resulting weighted migration matrices show the fractions of individuals born in each of the counties who stayed in the county of birth or moved to any other county (Figure S5).

Supplemental Note 3. Polygenic scores based on within-sibship GWAS summary statistics (sPGS)

Besides the 169 UKB-based PGSs and PGS_{EA4}, we calculated polygenic scores for 24 phenotypes using within-sibship GWAS summary statistics (sPGSs)[5]. The sPGSs were calculated for 24 out of 25 phenotypes analysed in the original study presenting a set of within-sibship GWAS results estimating direct genetic effects. The sPGS for C-reactive protein was excluded from the analysis due to an issue with the MCMC procedure in SBayesR when working with the respective summary statistics . Table S2 lists 24 traits with corresponding sample sizes. The methodology for calculating sPGS was the same as for the population-based PGSs (see Methods for details on PGS calculation).

sPGSs are less prone to population confounding, however, not necessarily represent direct genetic effects[7,8]. sPGSs are also substantially less powerful than population PGSs. For example, sPGS explains only 0.438% of EA variance in comparison with 5.4% for PGS_{EA} and 7.1% PGS_{EA4} in the set of unrelated Estonians. We conducted the analyses of Var_{county}, geographic distribution (for all the subsamples) and comparison of the migration groups (for the main Estonian subsample) with sPGSs. We note again that the sPGSs are not the estimates of the direct genetic effects on the respective traits. Thus, these analyses are meant to be the sensitivity tests as the underlying summary statistics come from a different source, contain more statistical noise but are less confounded.

Within-sibship GWAS summary statistics are available on OpenGWAS (https://gwas.mrcieu.ac.uk/), see the original study[5] for details.

Supplemental Note 4. Robustness of Var_{county} estimates

The main analysis of the Var_{county} of the PCs in the full Estonian sample shows that a significant fraction of the variance of all the top 100 PCs can be explained by POB and it is always higher than the fraction explained by POR. The same analysis for the PGSs shows that a significant fraction of the variance of most of the tested PGSs can be explained by POR. The fraction of variance explained by POR is higher than the fraction explained by POB for most of the cases when there is a significant difference between them. The highest Var_{county} for POB and POR is demonstrated by PGS_{EA}.

In this note, we demonstrate that neither participation bias nor the residual population structure are likely sources of the observed patterns. First, we present the analysis of Var_{county} for PCs, PGSs and sPGSs (Supplemental Note 3) in the subgroups described in Supplemental Note 1. Second, we explore how the number of PCs used for the PGS_{EA} adjustment affects the results. Third, we analyse the adjustment of PGS_{EA} for the complete genetic relatedness matrix (GRM) in a leave-one-chromosome-out (LOCO) approach.

Analysis of principal components and polygenic scores in subgroups

It has been shown that population-based cohorts tend to be biased, which can make generalization to the entire population uncertain. Among the characteristics most prone to bias are sex, age, SES[9,10]. Here, we present the analysis of Var_{county} for PCs, PGSs and sPGSs in the subgroups described in Supplemental Note 1. Briefly, we split the overall sample into subsamples by sex, age, year of recruitment (the corresponding groups differ in their average EA of the participants). We also conduct the analysis for the subcohort of self-reported Russian participants, which can be considered as an independent replication as the geographic distribution as well as historical background of this group is substantially different in comparison with the self-reported Estonian group. The analyses conducted on the subcohort of unrelated Estonians is presented in Supplemental Note 5, Figure S17. The results for the subsamples and for the Russian subsample are consistent in general. The comparison of the Estonian subcohorts shows that an increased rate of participation in the EstBB among certain subgroups in the population is unlikely to be the reason of the observed patterns, as this pattern is reproduced in the different groups. Despite the relatively small sample size of the Russian subsample, it also demonstrates patterns in their main aspects replicating the observations in the main Estonian sample. The analysis of sPGS is less stable due to the high proportion of statistical noise in the sPGS which is especially notable in small samples. The Var_{county} values presented in the plots can be found in Table S10.

Cumulative adjustment of PGS_{EA} for the top 100 PCs

Across the work, we adjust the PGSs for the top 100 PCs of the genetic relatedness matrix built on common SNPs to mitigate the effects of population genetic structure. Adjustment for 100 PCs is substantially more than usually used in the PGS analyses[11–14]. To demonstrate how adjustment for different numbers of PCs affects the Var_{county} of PGS_{EA} we calculated it for POB and POR with the PGS_{EA} adjusted for the top N principal components, where N ranges from 0 to 100 (Figure S15). PGS_{EA} was also adjusted for demographic covariates (sex, age, sex×age and age^2) in all the models. The set of 92,110 unrelated self-reported Estonian individuals was used. The greatest change in Var_{county} is observed within N < 5 while more PCs added have only minor effects, especially when N > 40. The difference between Var_{county} for POR and POB increases within N <= 10. After a moderate decrease from N = 11 to N = 15, it is rather stable and fluctuates between 0.75 and 0.76% which is more than 0.05% higher than before the adjustment. Note, that with 92,110 PCs (the number equal to the number of individuals), PGS_{EA} can be explained completely. Thus a gradual decline of Var_{county} for POB and POR as well as of the difference between them is expected. To conclude, the adjustment for the PCs decreases the Var_{county} of PGS_{EA} but increases the difference between PGS_{EA} Var_{county} for POR and POB. The most substantial effect is observed with adding roughly the first 10-15 PCs while adding further PCs has a minor effect.

Adjustment of PGS_{EA} for the complete genetic relatedness matrix (GRM) in a leave one chromosome out (LOCO) approach

Top 100 principal components (PCs) capture only a fraction of the population structure captured by the Genetic Relationship Matrix (GRM). An alternative approach to correcting a PGS for population structure is to use a Linear Mixed Model (LMM), which incorporates the entire Genetic Relationship Matrix (GRM). This approach has the potential to mitigate the effects of population structure on the PGS to a greater extent than using only the top PCs. However, regressing out too many PCs in a fixed-effect framework can lead to overcorrection[15]. In the limit, when the number of PCs equals the number of individuals, all the variance in the PGS can be explained.

To investigate how adjusting PGS_{EA} for different proxies of population genetic structure influences its Var_{county}, we compared the Var_{county} of PGS_{EA} corrected for the top 10 or 100 PCs, the GRM, or a combination of both in the set of unrelated individuals adjusted. To minimize proximal contamination, we employed the leave-one-chromosome-out (LOCO) approach.

GRMs were calculated on imputed genotypes using LDAK software version 5.1[16]. The imputed genotypes (as described in Methods) were filtered using PLINK2 to retain only biallelic single nucleotide polymorphisms (SNPs) with a minor allele frequency (MAF) >0.01 and a

Hardy–Weinberg equilibrium (HWE) p-value $>10^{-5}$. Next, SNPs were thinned using a squared correlation (r^2) threshold of 0.98 within a 100-kb window. Per-chromosome GRMs were calculated assuming LDAK model, assuming equal weights and scaling parameter (--power) of -0.25. These per-chromosome GRMs were then merged into 22 LOCO-GRMs.

Using LDAK, we fit the Restricted Maximum Likelihood (REML) models for per-chromosome PGS_{EA}. In these models, a respective LOCO-GRM was included as a random effect predictor, while fixed-effect covariates included demographic variables (sex, age, sex×age, age^2) and 10 genetic PCs. The independent residuals from these models were combined into a single PGS. This final PGS was either used as is ("demography, 10 PCs, GRM" in Figure S16) or further adjusted for 100 PCs ("demography, 100 PCs, GRM" in Figure S16).

We compared the Var_{county} of PGS_{EA} for POB and POR adjusted only for the demographic factors, adjusted for demographic variables and 10 or 100 PCs, and adjusted for demographic variables, 10 or 100 PCs, and the GRM (Figure S16). In both cases, adjustment for genetic PCs makes Var_{county} for POB and POR lower. Additional adjustment of PGS_{EA} for GRM makes Var_{county} even lower, however, this decrease is minor: the difference in Var_{county} with and without adjustment for the GRM ranges from 0.01% to 0.03%. It likely reflects the fact that the top 10 PCs already capture most of the structure affecting the Var_{county} of PGS_{EA}. Adjustment for the top 100 PCs in addition to the top 10 PCs in the presence or absence of the GRM has a larger effect on the Var_{county} (0.05-0.08%). It might show that adjustment for PCs is more effective than for GRM in the LOCO approach.

Regardless of the mechanisms, different approaches to adjustment of the PGS_{EA} for the genetic structure lead to only minor changes in Var_{county}. The adjustments decrease Var_{county} for both POB and POR and have almost no effect on the difference between them. Thus, neither PC adjustment nor GRM adjustment substantially impacts the signal of non-random geographic distribution of PGS_{EA} or the amplification of this signal resulting from contemporary migrations.

Both the PCA and GRM approach we applied here relied on the information on the common SNPs. It was shown that the recent population structure may be better captured by rare polymorphisms[17]. As the samples we work with here are only genotyped for common SNPs and using imputed genotypes for rare variants can potentially suffer from low accuracy, we abstained from using this approach. Using IBD segments can aid in capturing recent population structure. However, no single correction method fully eliminates residual population structure. Instead, to exclude all the potential effects of the population genetic structure and also of the parental (dynastic) effects, we used a within-sibling design (see the Main text).

Supplemental Note 5. Replication of the main results in unrelated Estonian individuals

Most of the statistical tests we use in this study require the independence of data points in the sample. In the main analyses, we assume that genetic relatedness in the sample does not affect the results substantially. This assumption is based on the population-based recruitment strategy of the EstBB and a substantial fraction of the overall population presented in the analysed sample (Supplemental Note 1). The former fact makes our estimates close to a description of the population. However, we note that for robust statistical inferences a sample with independent observations should be used. For this, we repeat all the main analyses in the sample of Estonian individuals with relatedness more distant than the 2nd-degree ("unrelated Estonian subsample/individuals"), assuming their independence.

All the results from the analyses with the subset of unrelated Estonians are consistent with the inferences from the overall Estonian sample. The key findings remain statistically significant.

Supplemental Note 6. Replication of the main results with PGS_{EA4}

The analysis of Var_{county} showed that SES-related PGSs demonstrate the highest increase in inter-regional variance with PGS_{EA} being the top signal. Most of the inter-regional variance of other PGSs can be explained through their correlation with PGS_{EA}. Thus, in the main text, we provide results of the population-level analyses with PGS_{EA}.

However, if the Var_{county} patterns for the tested PGSs are mostly linked to the EA-associated loci, a more powerful PGS for EA is expected to demonstrate an even stronger increase in Var_{county} due to migration. Also, correcting for a more powerful PGS for EA is expected to reduce the signal for other PGSs even further. To test this, we used the summary statistics from the EA4 GWAS[13] which were the most powerful summary statistics for EA available to us. Indeed, PGS_{EA4} demonstrates the highest Var_{county} for both POB and POR with a particular increase in Var_{county} for POR in comparison with that of PGS_{EA} (Figure S23A). In comparison with the adjustment for PGS_{EA}, the adjustment for PGS_{EA4} stronger decreases Var_{county} for other PGSs following the expectation (Figure S23B, Main Text).

As the EA4 GWAS is a meta-analysis of a large number of cohorts with various sample sizes, it is more prone to confounding due to residual population stratification. PGS_{EA} is based on the UK Biobank cohort GWAS and thus is less vulnerable to this effect[18,19]. Sibship-based analyses are robust towards residual confounding in PGSs thus we use PGS_{EA4} there for more statistical power. Other analyses that are described for PGS_{EA} are conducted also for PGS_{EA4}. We present their results in this Supplemental note.

Briefly, all the original results are reproduced with PGS_{EA4}. Moreover, absolute differences between groups by POB, POR, or migration profile for PGS_{EA4} are in general larger than for PGS_{EA} (Supplemental Figures 24 and 27). These differences also reach the threshold of significance more often (Supplemental Figures 24 and 26).

Supplemental Note 7. Analysis of psychiatric trait PGSs

The UK Biobank summary statistics used in this study include GWAS results for a few psychiatry-related traits, such as “Anxiety disorders”, “Alcohol-related disorders”, “Mental health problems ever diagnosed by a professional”, “Seen a psychiatrist for nerves, anxiety, tension or depression”. However, polygenic scores for some other conditions, such as schizophrenia[20] and attention deficit hyperactivity disorder (ADHD)[21], have been reported to be geographically clustered and associated with migration. To fill this gap, we conducted an additional analysis of variance explained by POB and POR for seven psychiatric conditions: anorexia nervosa, attention deficit hyperactivity disorder (ADHD), autism spectrum disorder (ASD), bipolar disorder, depressive symptoms, insomnia, and schizophrenia. We used polygenic scores from Polygenic Index Repository, calculated with SBayesR using GWAS meta-analysis summary statistics with the EstBB cohort excluded[22].

First, we estimated Var_{county} for the PGSs adjusted for demographic covariates and the first 100 PCs in the sample of self-reported Estonians (Figure S29A; Table S11) and in the subsample of unrelated Estonians (Figure S30A; Table S11). Four out of seven PGSs (for ADHD, insomnia, depressive symptoms, and anorexia nervosa) show a significantly higher Var_{county} for POR than for POB in both the entire sample and the sample of unrelated individuals. Bonferroni correction was done as for 170 tests for consistency with the main analysis including PGS_{EA4}. The Var_{county} estimates and their differences for all the psychiatric trait PGS are low in comparison with PGS_{EA4} (0.54% and 1.50% for POB and POR, respectively). Adjustment of the PGSs for PGS_{EA4} largely eliminates Var_{county} for POB and POR (Supplemental Figures 29B, 30B; Table S11). In the subsample of unrelated individuals, it makes Var_{county} statistically indistinguishable from zero for all the PGSs for POB and POR. In the overall sample, only PGS for depressive symptoms demonstrates Var_{county} significantly higher than zero for POB (Var_{county} = 0.0027%, p-value$_{Bonf}$ = 2.4×10^{-31}) but not for POR.

These results demonstrate that the average PGS value for certain psychiatric disorders differs in counties and these differences increase due to current migration. Moreover, these differences can be to a large extent indexed by PGS_{EA4}.

Supplemental Note 8. Selective migration and correlations between mate-pair PGSs

Assortative mating (AM) refers to genetic similarity between partners resulting from mate choice based on phenotype. It has been shown that for educational attainment (EA), the correlation between genetic predictors in partners is higher than expected based on phenotypic similarity[13,23]. Potential explanations for this include shared genetic ancestry between partners or assortment based on traits genetically correlated with EA.

Another factor contributing to genetic similarity between partners may be geographic clustering due to selective migration, as documented in this study and by Abdellaoui et al.[21]. Selective migration can lead to individuals with lower or higher PGSs for certain traits (particularly EA) being more prevalent in specific regions. Since individuals typically find partners relatively close to their place of residence, we expect a correlation between partners' PGSs even in the absence of AM, driven by mating by proximity. However, the consequences of this process should be the same as those of AM: genetic similarity between partners at loci associated with specific traits.

Since the strongest observed differentiation between geographic regions in Estonia occurs in the PGSs for EA (PGS_{EA} and PGS_{EA4}), we test our hypothesis using these PGS. Height and BMI are also well-known traits for which AM is observed in humans[23]. However, the observed differentiation due to contemporary migration is weaker for their corresponding PGSs (PGS_{height} and PGS_{BMI}). Therefore, we do not expect a strong effect of mating by proximity on partner similarity for these PGSs. For each of the four tested PGSs, we calculated correlations between randomly selected pairs of individuals from: (1) the overall Estonian population, (2) individuals born in the same county (based on place of birth, POB), and (3) individuals residing in the same county (based on place of residence, POR). We then compared these correlations with those of actual spouses, defined as pairs of individuals sharing a child in the EstBB (Figure S31). In both randomly selected pairs and spouses, the sex of individuals was not taken into account.

Correlations between pairs matched by POB as well as completely random pairs are not significantly different from zero for all the PGSs. However, the mean correlations for PGS_{EA} and PGS_{EA4} in pairs matched by POB are shifted towards positive values which is not observed for PGS_{height} and PGS_{BMI}. While correlations for PGS_{height} and PGS_{BMI} in pairs matched by POR remain statistically indistinguishable from zero, correlations for PGS_{EA} and PGS_{EA4} are significantly higher than zero. The absence of correlations among pairs matched by POB suggests that the correlations for pairs matched by POR are not the consequence of residual population structure. At the same time, the slight positive shift in average estimates for PGS_{EA} and PGS_{EA4} are likely caused by clustering due to migration in previous generations.

These results suggest that mating by proximity is a likely factor inflating estimates of the strength of AM for EA and potentially for other traits with a high genetic correlation with EA. Here, we selected pairs of individuals based on county-level geographic information. However, mating by proximity likely has a stronger influence on partner similarity at a finer scale, for example, due to residential segregation in cities.

Supplemental Note 9. How large are the regional differences in PGS_{EA}?

We can see that the regional differences in polygenic scores are statistically significant and are increasing due to migrations. However, if we compare polygenic scores for education attainment among people born and living in Tallinn and Tartu City versus those born and living in other regions (ORE) we see only a subtle difference in their distributions (Figure S32). Although those distributions are shifted, they overlap vastly. Practically, it means that a random individual born and residing in the city will have a polygenic score lower than that of a random individual born and residing outside the cities in close to 50% of the cases.

We would also like to stress that differences in PGS_{EA} between EstBB cohorts cannot be directly interpreted as regional differences in genetic predisposition to educational attainment in the general Estonian populations. First, although the Estonian Biobank includes approximately 20% of the country's adult population from a wide range of socioeconomic backgrounds and localities, the data set is not entirely representative of the Estonian population. Second, a polygenic score is a correlate of a trait and its genetic basis, not an exact genetic value. For any trait but especially for behavioural characteristics like educational attainment it accumulates a complex combination of direct and indirect effects as well as non-causal correlates. However, the polygenic scores based on the population-based genetic association study used in our analyses capture from 5 to 7% of individual differences in educational attainment (depending on underlying GWAS and analysed cohort). This is only a fraction of the total estimated genetic effect on educational attainment. Thus, PGS_{EA} from a population-based GWAS should be seen as a relatively weak, noisy and confounded proxy for the genetic predisposition of an individual that affects their EA.

Supplemental References

[S1]. Leitsalu, L., Haller, T., Esko, T., Tammesoo, M.-L., Alavere, H., Snieder, H., Perola, M., Ng, P.C., Mägi, R., Milani, L., et al. (2015). Cohort Profile: Estonian Biobank of the Estonian Genome Center, University of Tartu. Int. J. Epidemiol. *44*, 1137–1147. https://doi.org/10.1093/ije/dyt268.

[S2]. Milani, L., Alver, M., Laur, S., Reisberg, S., Haller, T., Aasmets, O., Abner, E., Alavere, H., Allik, A., Annilo, T., et al. (2025). The Estonian Biobank's journey from biobanking to personalized medicine. Nat. Commun. *16*, 3270. https://doi.org/10.1038/s41467-025-58465-3.

[S3]. Bycroft, C., Freeman, C., Petkova, D., Band, G., Elliott, L.T., Sharp, K., Motyer, A., Vukcevic, D., Delaneau, O., O'Connell, J., et al. (2018). The UK Biobank resource with deep phenotyping and genomic data. Nature *562*, 203–209. https://doi.org/10.1038/s41586-018-0579-z.

[S4]. Population census 2021 https://www.stat.ee/en/statistics-estonia/population-census-2021.

[S5]. Howe, L.J., Nivard, M.G., Morris, T.T., Hansen, A.F., Rasheed, H., Cho, Y., Chittoor, G., Ahlskog, R., Lind, P.A., Palviainen, T., et al. (2022). Within-sibship genome-wide association analyses decrease bias in estimates of direct genetic effects. Nat. Genet. *54*, 581–592. https://doi.org/10.1038/s41588-022-01062-7.

[S6]. Migration https://www.stat.ee/en/find-statistics/statistics-theme/population/migration.

[S7]. Veller, C., and Coop, G.M. (2024). Interpreting population- and family-based genome-wide association studies in the presence of confounding. PLoS Biol. *22*, e3002511. https://doi.org/10.1371/journal.pbio.3002511.

[S8]. Veller, C., Przeworski, M., and Coop, G. (2024). Causal interpretations of family GWAS in the presence of heterogeneous effects. Proc. Natl. Acad. Sci. U. S. A. *121*, e2401379121. https://doi.org/10.1073/pnas.2401379121.

[S9]. Pirastu, N., Cordioli, M., Nandakumar, P., Mignogna, G., Abdellaoui, A., Hollis, B., Kanai, M., Rajagopal, V.M., Parolo, P.D.B., Baya, N., et al. (2021). Genetic analyses identify widespread sex-differential participation bias. Nat. Genet. *53*, 663–671. https://doi.org/10.1038/s41588-021-00846-7.

[S10]. Schoeler, T., Speed, D., Porcu, E., Pirastu, N., Pingault, J.-B., and Kutalik, Z. (2023). Participation bias in the UK Biobank distorts genetic associations and downstream analyses. Nat Hum Behav *7*, 1216–1227. https://doi.org/10.1038/s41562-023-01579-9.

[S11]. Ni, G., Zeng, J., Revez, J.A., Wang, Y., Zheng, Z., Ge, T., Restuadi, R., Kiewa, J., Nyholt, D.R., Coleman, J.R.I., et al. (2021). A comparison of ten polygenic score methods for psychiatric disorders applied across multiple cohorts. Biol. Psychiatry *90*, 611–620.

https://doi.org/10.1016/j.biopsych.2021.04.018.

[S12]. Yengo, L., Vedantam, S., Marouli, E., Sidorenko, J., Bartell, E., Sakaue, S., Graff, M., Eliasen, A.U., Jiang, Y., Raghavan, S., et al. (2022). A saturated map of common genetic variants associated with human height. Nature *610*, 704–712. https://doi.org/10.1038/s41586-022-05275-y.

[S13]. Okbay, A., Wu, Y., Wang, N., Jayashankar, H., Bennett, M., Nehzati, S.M., Sidorenko, J., Kweon, H., Goldman, G., Gjorgjieva, T., et al. (2022). Polygenic prediction of educational attainment within and between families from genome-wide association analyses in 3 million individuals. Nat. Genet. *54*, 437–449. https://doi.org/10.1038/s41588-022-01016-z.

[S14]. Marston, N.A., Pirruccello, J.P., Melloni, G.E.M., Koyama, S., Kamanu, F.K., Weng, L.-C., Roselli, C., Kamatani, Y., Komuro, I., Aragam, K.G., et al. (2023). Predictive utility of a coronary artery disease polygenic risk score in primary prevention. JAMA Cardiol. *8*, 130–137. https://doi.org/10.1001/jamacardio.2022.4466.

[S15]. Freedman, D.A., Professor, and Freedman, D.A., Professor (1983). A note on screening regression equations. Am. Stat. *37*, 152–155. https://doi.org/10.1080/00031305.1983.10482729.

[S16]. Zhang, Q., Privé, F., Vilhjálmsson, B., and Speed, D. (2021). Improved genetic prediction of complex traits from individual-level data or summary statistics. Nat. Commun. *12*, 4192. https://doi.org/10.1038/s41467-021-24485-y.

[S17]. Zaidi, A.A., and Mathieson, I. (2020). Demographic history mediates the effect of stratification on polygenic scores. Elife *9*. https://doi.org/10.7554/eLife.61548.

[S18]. Sohail, M., Maier, R.M., Ganna, A., Bloemendal, A., Martin, A.R., Turchin, M.C., Chiang, C.W., Hirschhorn, J., Daly, M.J., Patterson, N., et al. (2019). Polygenic adaptation on height is overestimated due to uncorrected stratification in genome-wide association studies. Elife *8*, e39702. https://doi.org/10.7554/eLife.39702.

[S19]. Berg, J.J., Harpak, A., Sinnott-Armstrong, N., Joergensen, A.M., Mostafavi, H., Field, Y., Boyle, E.A., Zhang, X., Racimo, F., Pritchard, J.K., et al. (2019). Reduced signal for polygenic adaptation of height in UK Biobank. Elife *8*. https://doi.org/10.7554/eLife.39725.

[S20]. Colodro-Conde, L., Couvy-Duchesne, B., Whitfield, J.B., Streit, F., Gordon, S., Kemper, K.E., Yengo, L., Zheng, Z., Trzaskowski, M., de Zeeuw, E.L., et al. (2018). Association between population density and genetic risk for schizophrenia. JAMA Psychiatry *75*, 901–910. https://doi.org/10.1001/jamapsychiatry.2018.1581.

[S21]. Abdellaoui, A., Hugh-Jones, D., Yengo, L., Kemper, K.E., Nivard, M.G., Veul, L., Holtz, Y., Zietsch, B.P., Frayling, T.M., Wray, N.R., et al. (2019). Genetic correlates of social stratification in Great Britain. Nat. Hum. Behav. *3*, 1332–1342. https://doi.org/10.1038/s41562-019-0757-5.

[S22]. Alemu, R., Terskaya, A., Howell, M., Guan, J., Sands, H., Kleinman, A., Bann, D.,

Morris, T., Ploubidis, G.B., Fitzsimons, E., et al. (2025). An Updated Polygenic Index Repository: Expanded Phenotypes, New Cohorts, and Improved Causal Inference. bioRxiv. https://doi.org/10.1101/2025.05.14.653986

[S23]. Robinson, M.R., Kleinman, A., Graff, M., Vinkhuyzen, A.A.E., Couper, D., Miller, M.B., Peyrot, W.J., Abdellaoui, A., Zietsch, B.P., Nolte, I.M., et al. (2017). Genetic evidence of assortative mating in humans. Nat. Hum. Behav. *1*, 0016. https://doi.org/10.1038/s41562-016-0016.
